# AhR‐Dependent Induction of β‐Defensin 1 in Colonic Epithelial Cells Regulates Cross‐Talk between Gut Microbiota and Immune Response Leading to Attenuation of Colitis

**DOI:** 10.1002/advs.202416324

**Published:** 2025-05-23

**Authors:** Manikandan Palrasu, Amarnath Marudamuthu, Khadija Kakar, Hamida Hamida, Shruthi Thada, Rohan Gupta, Kiesha Wilson, Taylor Carter, Yin Zhong, Archana Saxena, Xiaoming Yang, Narendra Singh, Philip Brandon Busbee, Jie Li, Monica Garcia‐Buitrago, Prakash Nagarkatti, Mitzi Nagarkatti

**Affiliations:** ^1^ Department of Pathology Microbiology and Immunology University of South Carolina School of Medicine Columbia SC 29208 USA; ^2^ Department of Chemistry and Biochemistry University of South Carolina Columbia SC 29208 USA; ^3^ Department of Pathology University of Miami Miller School of Medicine Miami FL 33136 USA

**Keywords:** aryl hydrocarbon receptor, Beta‐defensin1, Crohn's disease, microbial dysbiosis, ulcerative colitis (UC)

## Abstract

The aryl hydrocarbon receptor (AhR) acts as a critical signaling hub that connects immune cells, food and environmental cues, and microbiota to regulate intestinal homeostasis. In the current study, the role of AhR in the regulation of an antimicrobial peptide, β‐defensin1 (BD‐1) is investigated to control colitis. Human patients with ulcerative colitis (UC) and Crohn's disease (CD), and mice with three different models of colitis, express a significant decrease in the expression of BD‐1 in colonic epithelial cells (CECs). Dietary and environmental AhR ligands induce the expression of BD‐1 in CECs through the activation of two dioxin‐responsive elements (DREs) expressed on its promoter. AhR ligands attenuate colitis in wild‐type (WT) mice while inducing BD‐1. However, AhR ligands fail to induce BD‐1 and protect mice from colitis when there is an intestinal epithelial cell (IEC)‐specific deletion of AhR. Blocking BD1 in vivo using antibodies prevents the ability of AhR ligands to ameliorate colitis, restore dysbiosis, and attenuate colonic inflammation. The current study identifies a novel pathway involving dietary, environmental, and endogenous AhR ligands to induce the antimicrobial peptide BD‐1 in IECs, which in turn, plays a critical role in the regulation of intestinal homeostasis.

## Graphical abstract

This study identifies a key signaling pathway involving the environmental sensor AhR and antimicrobial peptides, such as BD‐1, in the regulation of intestinal homeostasis. It highlights the role of BD‐1 in regulating gut microbiota and the intestinal immune response. Targeting this pathway presents potential therapeutic strategies for the treatment of gut disorders, including colitis and colon cancer.

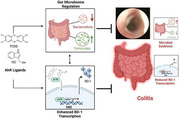

## 1 Introduction

Inflammatory bowel disease (IBD), a chronic inflammatory disease of the gastrointestinal tract, increases the risk of developing colon cancer.^[^ ^1^ ^]^ Based on the affected areas of inflammation and the extent of tissue loss in the gastrointestinal tract, IBD can be divided into Ulcerative colitis (UC) and Crohn's disease (CD). In contrast to CD, which causes inflammation and damages the entire gastrointestinal tract, UC‐associated inflammation and damage is restricted to the colon and rectum, with damage occurring at the epithelial surface.^[^ ^2^ ^]^ The estimated prevalence of UC in North America is 0.4% of the population, or 1.5 million people, with a yearly incidence of 15 per 100 000 people.^[^ ^3^ ^]^ Although the etiology of colitis is not fully understood, several studies suggest that the multifaceted interplay between microbial, environmental, and a host genetic factor underlies the development of colitis.^[^ ^2^, ^4^ ^]^

Complex microbial communities inhabit the human gastrointestinal tract; some of these communities are crucial for maintaining human health, while others induce gut disease.^[^ ^5^ ^]^ While butyrate‐producing bacteria have been demonstrated to lessen symptoms in UC colitis patients^[^ ^6^ ^]^ and in animal models of colitis,^[^ ^7^ ^]^ bacterial species that produce lipopolysaccharide (LPS) are known to promote colitis in animal models.^[^ ^8^ ^]^ Several studies have shown that dysregulated immune response, which may be triggered by gut microbial dysbiosis, induces chronic gut inflammation and colitis.^[^ ^6^, ^7^ ^]^ Adding to the complexity of gut homeostasis is the role played by colonic epithelial cell (CEC)‐derived antimicrobial compounds.

The intestinal epithelium, which plays a key role in host defense and acts as the gut mucosa's physical and immunological barrier, can produce antimicrobial molecules such as α‐ and β‐defensins (BDs) for epithelial protection from commensal microorganisms.^[^ ^9^ ^]^ Specialized epithelial cells, known as Paneth cells, are responsible for producing α‐defensins from the bottom of Lieberkühn's crypts in the small intestine, while regular columnar epithelial cells in both the small and large intestines secrete β‐defensins.^[^ ^9a^ ^]^ A comparatively large number of β‐defensins are expressed by both humans and mice; six human (hBD1‐6) and five mouse (mBD1‐5) β‐defensins have been identified and characterized. While other BDs, such as hBD‐2 and its orthologue mBD‐3, are triggered by bacteria and cytokines, hBD‐1 and its orthologue mBD‐1 are produced constitutively.^[^ ^10^ ^]^ Several studies have shown that BDs exhibit antibacterial activity against gram‐negative bacteria.^[^ ^10^, ^11^ ^]^ Little is currently known about how BDs are regulated, and it is not evident if the gut microbiota is necessary to produce these antimicrobial compounds.

Aryl hydrocarbon receptor (AhR), a ligand‐dependent transcription activator, plays an important role as an environmental sensor and a regulator of the immune response.^[^ ^12^ ^]^ AhR is a cytosolic protein that is activated by its ligands and moves into the nucleus, where it attaches to genes that have the dioxin response elements (DREs) and transactivates them.^[^ ^12^, ^13^ ^]^ AhR‐deficient mice and AhR antagonists have been shown to exhibit more severe colitis, and AhR activation may be a vital prophylactic and therapeutic measure against colitis.^[^ ^14^ ^]^ AhR activation was shown to manipulate the delicate balance between pathogenic T cells (Th17) and anti‐inflammatory T cells (Tregs), thus preventing UC.^[^ ^7^, ^15^ ^]^ AhR ligands have been found to decrease pro‐inflammatory cytokines including TNFα, IL‐6, IL‐12, IFNγ, IL‐7, and IL‐17, hence reducing fibrosis and microbial translocation in the gut.^[^ ^15^, ^16^ ^]^ Moreover, AhR activation by its ligands, including the microbial metabolites, leads to increased levels of anti‐inflammatory regulatory mechanisms such as IL‐10, IL‐22, and Foxp3, which aid in the restoration of gut homeostasis in colitis mouse models.^[^ ^7^, ^17^ ^]^ Recently, Wang et al. demonstrated that AhR/IL‐22/Stat3 signaling pathway modulated intestinal mucosa antimicrobial molecules by commensal microbiota.^[^ ^18^ ^]^

It is interesting to note that patients with IBD showed decreased expression of AhR,^[^ ^19^ ^]^ and hBD‐1.^[^ ^20^ ^]^ Moreover, a genetic variation in the gene encoding hBD‐1 is associated with IBD.^[^ ^21^ ^]^ However, whether AhR activation regulates secretion of antimicrobial peptides such as BD‐1 in CECs during colitis has not been previously investigated.

In this study, we investigated the role of AhR activation on induction of BD‐1 and the consequent effect on gut microbiota and colonic inflammation. Our data demonstrated that AhR transcriptionally regulates the production and secretion of antimicrobial BD‐1, which limits gut dysbiosis and attenuates colitis.

## 2 Results

### 2.1 UC Patients Exhibit Decreased Levels of AhR and hBD‐1 in the Colon

**Figure 1:**
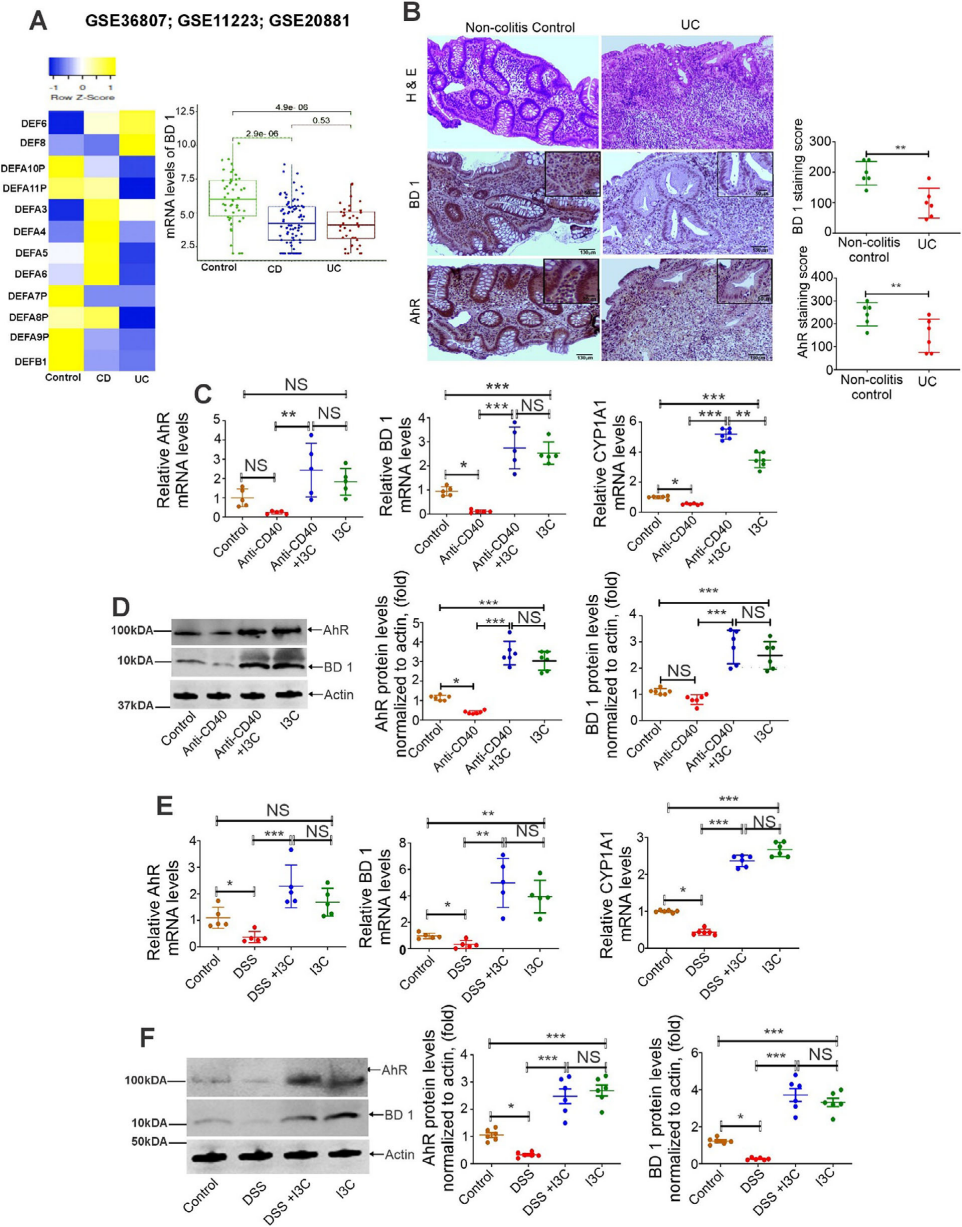
Expression of AhR and BD‐1 in colitis patients and CECs from control and colitis mice. A) Analyses of the Transcriptome profiling data of CD (*n* = 66) and UC (*n* = 82) patients versus healthy control samples (*n* = 61). Hierarchical clustering heatmap (Left panel) and Boxplot of BD‐1 (Right panel) are shown and analyzed through “limma” package taking adjusted *p*‐value < 0.05 in DEGs Analysis. B) Representative IHC staining for AhR and BD‐1 proteins in colonic biopsies collected from non‐colitis controls, and UC patients. Insets show magnified views, × 40. Scale bars: 50 µm. Dot plots show IHC scores for expression of the indicated proteins (*n*  = 12; 6 samples/group). Data were analyzed using unpaired 2‐tailed t‐test; Data are displayed as mean ± SD. **p < 0.01. C) The mRNA expression of AhR, BD1, and CYP1A1 in anti‐CD40‐induced colitis model with or without treatment with AhR ligand I3C (*n* = 5). D) The protein expression of AhR, and BD1 in anti‐CD40‐induced colitis model with or without treatment with AhR ligand I3C (*n* = 6). E) mRNA expression of AhR, BD1, and CYP1A1 in DSS‐induced colitis model with or without treatment with AhR ligand I3C (*n* = 5). F) The protein expression of AhR, and BD1 in DSS‐induced colitis model with or without treatment with AhR ligand I3C (*n* = 6). The graph panels for western blot show quantification of AhR and BD1 protein by densitometry, normalized to actin. Statistical significance was calculated using 1‐way ANOVA followed by Tukey's multiple comparison test. Data are displayed as mean ± SD. *p < 0.05; **p < 0.01, ***p < 0.001, NS = Not significant.

Given the important role played by antimicrobial peptides that resist microbial colonization of epithelial surfaces in the regulation of host defense immune response,^[^ ^9a,b^ ^]^ we first enrolled a public data set to investigate differential expression of various antimicrobial peptides in patients with UC, CD, and control subjects, from the Gene Expression Omnibus (GEO) (GSE36807; GSE20881; GSE11223). Our analysis of colonic specimens from 61 controls, 82 UC patients, and 66 CD patients found that the levels of several AMPs were dysregulated in UC and CD patients when compared to the controls (**Figure** **1**A left panel). Further focusing on the expression of hBD‐1, we noted that hBD‐1 was down‐regulated in UC and CD patients (Figure 1A right panel). Because the samples we screened from UC and CD patients were whole colonic tissue, and we were interested in CECs, we next analyzed the expression hBD‐1 protein using immunohistochemistry (IHC) in colonic biopsies collected from UC patients and non‐colitis‐control subjects (12 patients; 6/group). The colonic biopsies showed severe active colitis featuring crypt distortion, increased lymphoplasmacytic infiltrate in lamina propria, cryptitis, crypt abscesses, surface epitheliitis, and ulceration (Figure 1B). Control biopsies demonstrated normal crypt architecture and lack of inflammatory infiltrates in lamina propria and epithelium (Figure 1B). IHC analysis revealed cytoplasmic and nuclear expressions of hBD‐1 in the epithelium of non‐colitis cases. We also observed similar results in CD patients (Figure S1A, Supporting Information). Consistent with mRNA studies (Figure 1A), we found that hBD‐1 expression was downregulated in epithelial cells in UC and CD when compared to non‐colitis and non‐CD controls, respectively (Figure 1B; Figure S1A, Supporting Information). In such epithelial cells, we also noted decreased expression of AhR (Figure 1B).

#### 2.1.1 AhR Activation Increases the Expression of mBD‐1 in CECs

To further mechanistically characterize the role of AhR and mBD‐1, we extended the human studies in the murine models of colitis. Specifically, we investigated the expression of AhR and mBD‐1 in the CECs, the primary source of these molecules. To that end, mice with Anti‐CD40‐ and DSS‐induced colitis were treated with I3C, a well characterized AhR ligand.^[^ ^7^, ^22^ ^]^ We used four groups of mice in both the Anti‐CD40 and DSS‐induced colitis models. In the Anti‐CD40 model, Group 1 received i.p. injections of 0.05% DMSO/corn oil and Rat IgG2a isotype control (200 µg in PBS). Group 2 was injected with anti‐CD40 monoclonal antibody FGK45 (200 µg in PBS) and 0.05% DMSO/corn oil. Group 3 was pretreated with I3C (40 mg k^−1^g in 0.05% DMSO/corn oil) 48 h before anti‐CD40 and continued every other day. Group 4 received I3C and Rat IgG2a control. In the DSS model, Group 1 received vehicle (0.05% DMSO/corn oil), Group 2 received DSS (3%) and vehicle, Group 3 received I3C (40 mg k^−1^g i.p. in 0.05% DMSO/corn oil) after 1 h of DSS introduction and continued every other day, and Group 4 received I3C alone. CECs from control and experimental animals were isolated and identified as EPCAM(+) cells using flow cytometry, as shown in Figure S1B (Supporting Information). These cells were then used for RNA sequencing analysis and subsequent analysis of AhR and BD‐1 mRNA and protein expression. Using RNA sequencing analysis, we identified more than 200 robust genes whose expression was induced in anti‐CD40‐mediated colitis in CECs, and which were reversed following treatment with I3C, as shown in the heatmap (Figures S2A and S2B, Supporting Information). Further focusing on AhR and mBD‐1, the data showed that the epithelial cells from anti‐CD40‐induced colitis mice exhibited decreased levels of AhR and mBD‐1 mRNA (Figure 1C) and protein expression (Figure 1D) when compared to the controls. Also, treatment of anti‐CD40‐induced colitis mice with I3C caused an increase in the expression of AhR and mBD‐1 (Figures 1C,D). We found similar results in the DSS‐mediated colitis model (Figure 1E,F). We assessed AhR activation by measuring the mRNA expression of its target gene, CYP1A1, which was notably reduced in mice with colitis. However, treatment with I3C led to a significant increase in CYP1A1 expression (Figures 1C,E). It was also interesting to note that overall, I3C treatment alone induced the expression of AhR and mBD‐1 when compared to the controls (Figures 1C–F).

To further confirm the role of AhR in the induction of mBD‐1, we used 2,3,7,8‐tetrachlorodibenzo‐p‐dioxin (TCDD), a potent and well‐established AhR ligand. To that end, we treated mice having anti‐CD40/ DSS‐induced colitis with TCDD and analyzed the AhR and mBD‐1 mRNA and protein expression in the CECs, which were identified by EPCAM(+) cells using flow cytometry as shown in Figures S1C and S1D (Supporting Information). Similar to I3C, our data showed that TCDD upregulated AhR, CYP1A1 and mBD‐1 in CECs from control and colitis (anti‐CD40/ DSS‐ induced) animals (Figures S2C, S2D, S2G, and S2H, Supporting Information). We also observed similar results in intestinal epithelial cells (IEC) from control and colitis (anti‐CD40/ DSS‐ induced) animals (Figures S2E, S2F, S2I, and S2J, Supporting Information). Together, these data demonstrated that during colitis, mBD‐1 is downregulated and AhR ligands such as I3C or TCDD can induce the expression of mBD‐1 in CECs.

**Figure 2:**
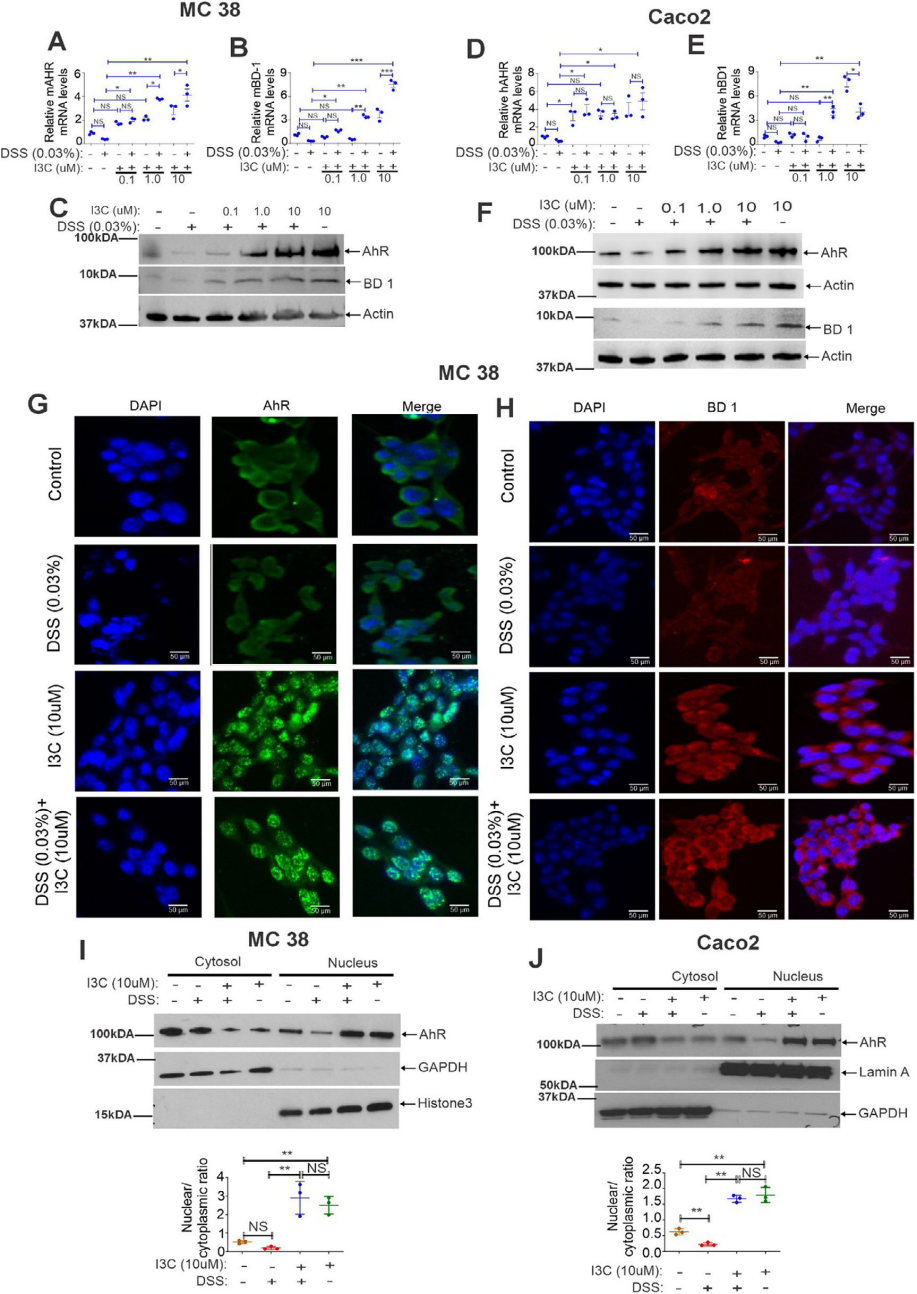
I3C treatment increases the expression of AhR and BD‐1 at both mRNA and protein levels in epithelial cells. MC38 cells were pretreated with I3C at indicated concentrations (0.1, 1, and 10 µm) and then treated with DSS (0.03%) for an additional 16 h. A–C) The mRNA (A, B) and protein (C) expression of AhR and BD‐1 were analyzed by real‐time PCR and western blot analysis respectively (*n* = 3). D–F) The same as A‐C, but Caco2 cells were used (*n* = 3). G,H) I3C treatment of MC38 cells leads to AhR nuclear translocation and induction of BD‐1. MC38 cells were pretreated with I3C (10 µm), for 2 h at indicated concentrations, and then treated with DSS (0.03%) for an additional 16 h. Protein expressions of G) AhR and H) BD‐1 were analyzed by immunofluorescence staining (*n* = 3). I) The same as G, but nuclear and cytosolic fractions were isolated and analyzed for the localization of AhR (*n* = 3) by western blot analysis. J) The same as I, but Caco2 cells were used for the analysis of localization of AhR (*n* = 3) by western blotting. The graph panels for western blot show quantification of nuclear to cytoplasmic ratio of AhR protein by densitometry, normalized to respective housekeeping proteins. Data are shown as mean ± SEM, and significance was determined using 1‐way ANOVA and Tukey's multiple comparisons test; **p < 0.05; ***p < 0.01; ***p < 0.001. NS = Not significant.

To directly test if the expression of BD‐1 is regulated by AhR, we used murine adenocarcinoma cells, MC38, and human colon carcinoma, Caco2 cells which were treated with I3C at 0.1, 1 and 10 µm and then treated with DSS (0.03%) for additional at 8, 16, and 24 h, to assess their impact on AhR expression in MC38 and Caco‐2 cells (data not shown). Our analysis revealed that I3C treatment at 10 µm and DSS at 0.03% for 16 h significantly altered AhR expression (data not shown). Based on these findings, we decided to use the 16‐h treatment with I3C and DSS for the entire analysis. Similar to in vivo colitis models, our analysis found that DSS+I3C treatment significantly increased the mRNA (**Figures** **2**A,B,D,E) and protein expression (Figures 2C,F) of AhR and BD‐1 in CECs MC38 (Figures 2A–C) and Caco2 (Figures 2D–F) when compared to DSS alone, especially at higher doses of I3C. Also, similar to the in vivo colitis studies, I3C alone was also able to upregulate AhR and BD‐1 at higher concentrations (Figures 2A‐2F).

When the AhR pathway is activated, AhR accumulates and translocates into the nucleus to induce expression of its target genes. Using immunofluorescence staining, we observed cytoplasmic localization of AhR in untreated MC38 (Figures 2G,I) and Caco2 cells (Figure 2J). I3C treatment of these cells decreased the expression of AhR in the cytosol while increasing the nuclear accumulation of AhR (Figures 2G,I), which was associated with upregulation of BD‐1 (Figure 2H) in MC38 cells. We also carried out a nuclear and cytosolic fractionation assay in MC38 and Caco2 cells. We observed a significant increase in the AhR protein levels in the nucleus after I3C treatment in MC38 cells (Figure 2I). Similar to MC38 cells, we detected a significant increase in the AhR protein levels in the nucleus after I3C treatment in Caco2 cells (Figure 2J). These data suggested that I3C induced the activation and nuclear accumulation of AhR and induction of BD‐1 in CECs.

### 2.2 AhR Activation Transcriptionally Upregulates BD‐1 Expression in CECs

**Figure 3:**
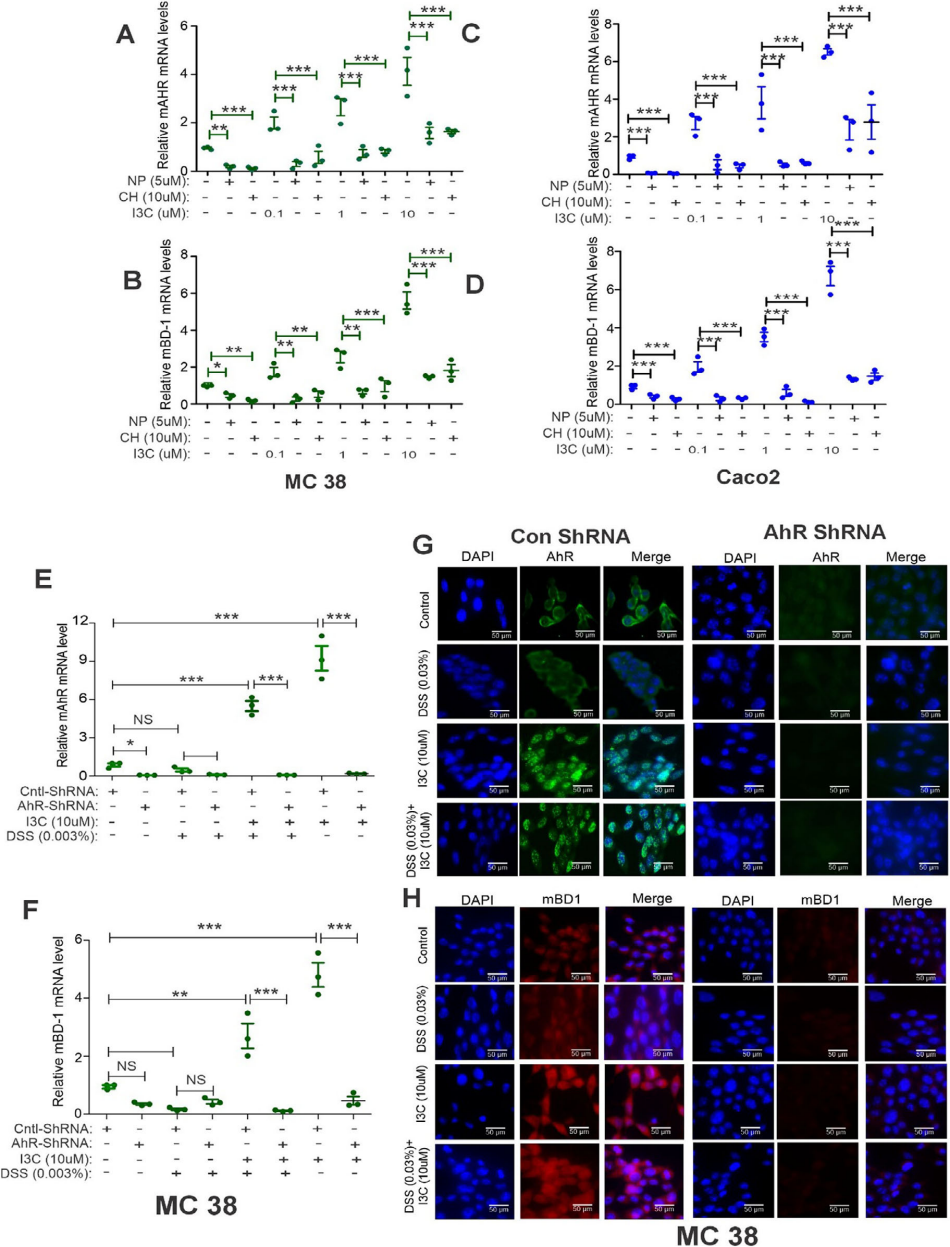
Blocking AhR attenuates I3C‐mediated upregulation of BD‐1 in CECs. A,B) MC38 cells were pretreated with I3C, AhR antagonists, α‐Naphthoflavone (NP; 5 µm) or CH223191 (CH; 10 µm) for 2 h at indicated concentrations, and then treated with DSS (0.03%) for additional 16 h. The mRNA expression of A) AhR and B) BD1 was analyzed by real‐time PCR. C,D) The same as A and B, but Caco2 cells were used. E,F) MC38 cells stably transfected with AhR shRNA or control shRNA were pretreated with I3C, at indicated concentrations, and then treated with DSS (0.03%) for an additional 16 h. The mRNA of E) AhR and F) BD1 was analyzed by real‐time PCR. G,H) The same as E and F, but the protein expression of G) AhR and H) BD‐1 was analyzed by immunofluorescence staining (*n* = 3). Data are shown as mean ± SEM, and significance was determined using 1‐way ANOVA and Tukey's multiple comparisons test; **p < 0.05; **p < 0.01; ***p < 0.001. NS = Not significant.

To further confirm that I3C was acting through AhR to induce BD‐1, we performed studies using AhR antagonists. To that end, MC38 (**Figures** **3**A,B) and Caco2 (Figures 3C,D) cells were pretreated with AhR antagonists, α‐Naphthoflavone (NP; 5 µm) or CH223191 (CH; 10 µm) for 2 h and then treated with I3C at the indicated concentrations. The mRNA expression of AhR and BD‐1 was analyzed by real‐time PCR. I3C treatment induced the expression of AhR and BD‐1 in a dose‐dependent manner, but the induction BD‐1 was reduced in cultures containing antagonists (Figures 3A–D). We validated our findings using siRNA experiments in which AhR was downregulated with siRNA in MC38 and Caco2 cells, which were then pretreated with I3C at indicated concentration for 2 h and then treated with DSS (0.03%) for an additional 16 h. Transfections with scrambled siRNA were used as controls. The mRNA expression of AhR and BD‐1 was analyzed by real‐time PCR. Our analyses showed that the ability of I3C to induce BD‐1 was significantly blocked in AhR‐downregulated MC38 (Figures S3A and S3B, Supporting Information) and Caco2 (Figures S3C and S3D, Supporting Information) cells. We also analyzed the AhR and BD‐1 protein expression in AhR‐downregulated MC38 cells as shown in Figures S4A and S4B (Supporting Information). Our analysis found that inhibition of AhR attenuates the stimulatory effect of I3C and leads to a marked downregulation of BD‐1 protein (Figure S4B, Supporting Information). We also stably expressed shRNA against AhR (Figures 3E,G). These experiments further confirmed that the ability of I3C to induce mRNA and protein expression of BD‐1 was significantly dampened in AhR‐downregulated MC38 cells (Figures 3E–H).

**Figure 4:**
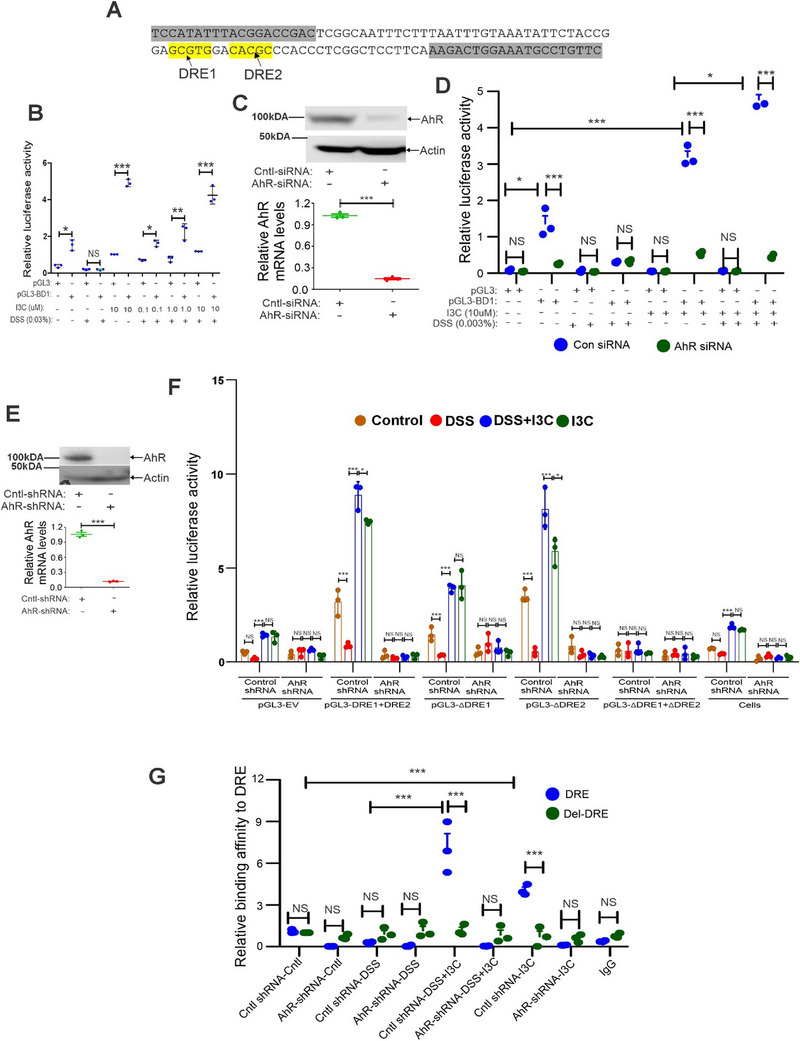
AhR transcriptionally regulates BD‐1 in MC38 cells. A) In silico analysis revealed two DREs 9 kb upstream of the BD‐1 transcription start point. B) MC38 cells were transfected with BD‐1‐promoter luciferase reporter plasmid and then treated with I3C and DSS at indicated concentration and BD‐1 promoter luciferase reporter assays were performed (*n* = 3). C) The transfection efficiency of the reporter assay for AhR siRNA was measured via western blotting analysis with antibodies against AhR (upper panel) and RT‐PCR analysis of AhR mRNA expression (bottom panel). D) Same as A and B, but MC38 cells transiently transfected with AhR siRNA or control siRNA were used (*n* = 3). E) The transfection efficiency of the reporter assay for AhR‐shRNA was measured via western blotting analysis with antibodies against AhR (upper panel) and Real time‐PCR analysis of AhR mRNA expression (bottom panel). F) Same as A and B, but MC38 stably transfected with AhR shRNA or control shRNA were transfected with pGL3‐DRE1+DRE2, pGL3‐ΔDRE1, pGL3‐ΔDRE2, and pGL3‐ΔDRE1+ΔDRE2 BD1 promoter constructs (as described in methods) and then treated with I3C and DSS at indicated concentration (*n* = 3). G) Chromatin immunoprecipitation (ChIP) assay using AhR antibody was performed, followed by qPCR applying primers covering DREs and absence of DREs (Del‐DRE) regions (*n* = 3) in MC38 cells. Data are shown as mean ± SEM, and significance was determined using 1‐way ANOVA and Tukey's multiple comparisons test; **p < 0.01; **p < 0.01; ***p < 0.001.

To check if I3C‐mediated AhR activation induces BD‐1 through DREs, we performed in silico analysis and found that there are two DREs located ≈9 kb upstream of transcription start site (TSS) of mBD‐1 (**Figure** **4**A). To confirm whether I3C treatment activated BD‐1 promoter, we constructed a mBD‐1 promoter with two DRE‐containing luciferase reporter and transfected into MC38 cells and then treated with I3C and DSS at indicated concentration for 16 h. The results confirmed that I3C treatment upregulated mBD‐1 promoter activity in a dose‐dependent manner (Figure 4B). To validate further how AhR is involved in the regulation of mBD‐1 promoter activity, we transfected mBD‐1 promoter with DRE‐containing luciferase reporter into MC38 cells, in which AhR was transiently downregulated using specific siRNA (Figure 4C). These cells were treated with I3C and DSS at indicated concentrations. We consistently observed that the activation of AhR by I3C significantly induced mBD1 promoter activity in MC38 cells transfected with control siRNA. Conversely, transient downregulation of AhR inhibited mBD‐1 promoter activity (Figure 4D). Next, we investigated to find out which DRE region mediates the transactivation of the AhR. We therefore generated four mBD‐1 promoter constructs, which we named pGL3‐DRE1+DRE2, pGL3‐ΔDRE1, pGL3‐ΔDRE2, and pGL3‐ΔDRE1+ΔDRE2 based on the presence and absence of DRE regions. We transfected these BD1 promoter reporters into MC38 cells, in which AhR was stably downregulated using specific shRNA (Figure 4E). The results confirmed that I3C treatment upregulated mBD‐1 promoter activity in control shRNA cells transfected with either pGL3‐ΔDRE1 or pGL3‐ΔDRE2 or both (pGL3‐DRE1+DRE2), but not in AhR downregulated cells (Figure 4F). In addition, I3C treatment did not induce mBD‐1 promoter activity in cells transfected with pGL3‐ΔDRE1+ΔDRE2 (Figure 4F), suggesting that both DRE regions are involved in AhR‐mediated induction of mBD‐1. We also performed ChIP assay in control and AhR‐downregulated cells (AhR shRNA cells) treated with I3C and DSS at indicated concentrations. The results demonstrated direct binding of AhR on mBD‐1‐promoter with DRE regions in control shRNA‐MC38 cells, compared with the AhR‐downregulated cells (Figure 4G). Taken together, these multiple lines of evidence demonstrated that AhR activation directly induces the expression of mBD‐1, involving DREs, in CECs.

### 2.3 AhR‐Dependent Upregulation of mBD‐1 during Colitis In Vivo

To assess the biological impact of mBD‐1 upregulation during colitis, we used an anti‐CD40‐induced colitis model, and analyzed the various clinical parameters, as shown in Figure S5 (Supporting Information). The animals were randomized into four groups. Group 1 consisted of controls. Animals in group 2 were given Anti‐CD40+Vehicle used for dissolving I3C (shown as Anti‐CD40 in Figures). Animals in group 3 were pretreated with I3C, 24 h before administration of Anti‐CD40. Group 4 animals were given I3C alone (Figure S5A, Supporting Information). Studies have shown that activation of CD40 signaling using anti‐CD40 antibody can cause colitis in T cell‐ and B‐cell‐deficient mice by increasing myeloid cell‐mediated production of IL‐23, IL‐1β, and IL‐12.^[^ ^23^ ^]^ Our analysis detected a significant reduction in body weight (Figure S5B, Supporting Information), colon length (Figure S5C, Supporting Information) and an increased macroscopic colitis score (Figure S5D, Supporting Information) in anti‐CD40‐induced colitis mice. However, I3C treatment reduced the percentage of weight loss (Figure S5B, Supporting Information), colon shortening (Figure S5C, Supporting Information), and macroscopic colitis score (Figure S5D, Supporting Information). To determine the extent of gut damage, we performed FITC‐dextran gut permeability assay (Figure S5E, Supporting Information) and measured circulating levels of serum amyloid A (SAA) (Figure S5F, Supporting Information). We found increased FITC‐dextran (Figure S5E, Supporting Information) and SAA (Figure S5F, Supporting Information) levels in serum of colitis mice; however, colitis mice treated with I3C exhibited decreased circulating serum levels of FITC‐dextran and SAA (Figures S5E and S5F, Supporting Information). Colonoscopic (Figures S5G and S5H, Supporting Information) and histopathological data (Figures S5I and S5J, Supporting Information) revealed the ulceration and tissue sloughing in the lining of the colon, tissue destruction in mucosa, submucosa, and LP layers; loss of crypts; and increased evidence of cellular infiltration in colitis mice when compared with controls. I3C treatment, however, maintained crypt formation and normal colonic tissue architecture, and they showed reduced signs of cellular infiltration (Figures S5G, S5H, S5I, and S5J, Supporting Information). Additionally, anti‐CD40‐induced colitis mice showed significantly higher levels of mRNA expression of proinflammatory cytokines IL‐1β (Figure S5K, Supporting Information), IFNγ (Figure S5L, Supporting Information), and TNFα (Figure S5M, Supporting Information) in colonic mucosa when compared to controls. Also, I3C therapy significantly reduced these cytokines in anti‐CD40‐induced colitis mice (Figures S5K–M, Supporting Information).

Pretreatment with AhR ligand can reduce immune response or other biological functions in mice, making them less likely to develop colitis. To exclude this possibility and investigate whether post‐treatment with I3C would also attenuate colitis, we induced colitis using administration of Anti‐CD40 and treated these mice with I3C on day 6 and continued every day for another 8 days, as shown in Figure S6A (Supporting Information). We analyzed the colitis associated symptoms and mRNA and protein expression of AhR and BD1 in CECs from control and experimental animals. Like pretreatment, post‐treatment of colitis mice with I3C suppressed colitis and colitis associated symptoms (Figures S6B–S6I, Supporting Information) and increased the expression of AhR and BD1 at both mRNA (Figure S6J, Supporting Information) and protein (Figure S6K, Supporting Information) levels.

We also employed the DSS‐induced colitis model to see if I3C was equally effective against it. We discovered that I3C was also able to suppress DSS‐induced colitis as evidenced by reversal of all colitis‐related pathology such as weight loss, colon length, macroscopic colitis score, SAA level, infiltration of immune cells, and maintained normal crypt formation and colonic tissue architecture (Figures S7A–S7J, Supporting Information). In addition, I3C therapy significantly reduced the mRNA expression of proinflammatory cytokines IL‐1β (Figures S7K, Supporting Information), IFNγ (Figure S7L, Supporting Information), and TNFα (Figure S7M, Supporting Information) in DSS‐induced colitis mice. To further corroborate the role of AhR activation, we used a potent AhR ligand, TCCD, to test its effect on anti‐CD40 (Figures S2C–S2F and S8A–S8K, Supporting Information) and DSS (Figures S2G–S2J and S9a–S9K, Supporting Information)‐induced colitis mouse models. Similar to I3C, our data showed that TCDD suppressed colitis (Figures S8A–S8K and S9A–S9K, Supporting Information), which was associated with the induction of mBD‐1 (Figures S2C–S2J, Supporting Information).

**Figure 5:**
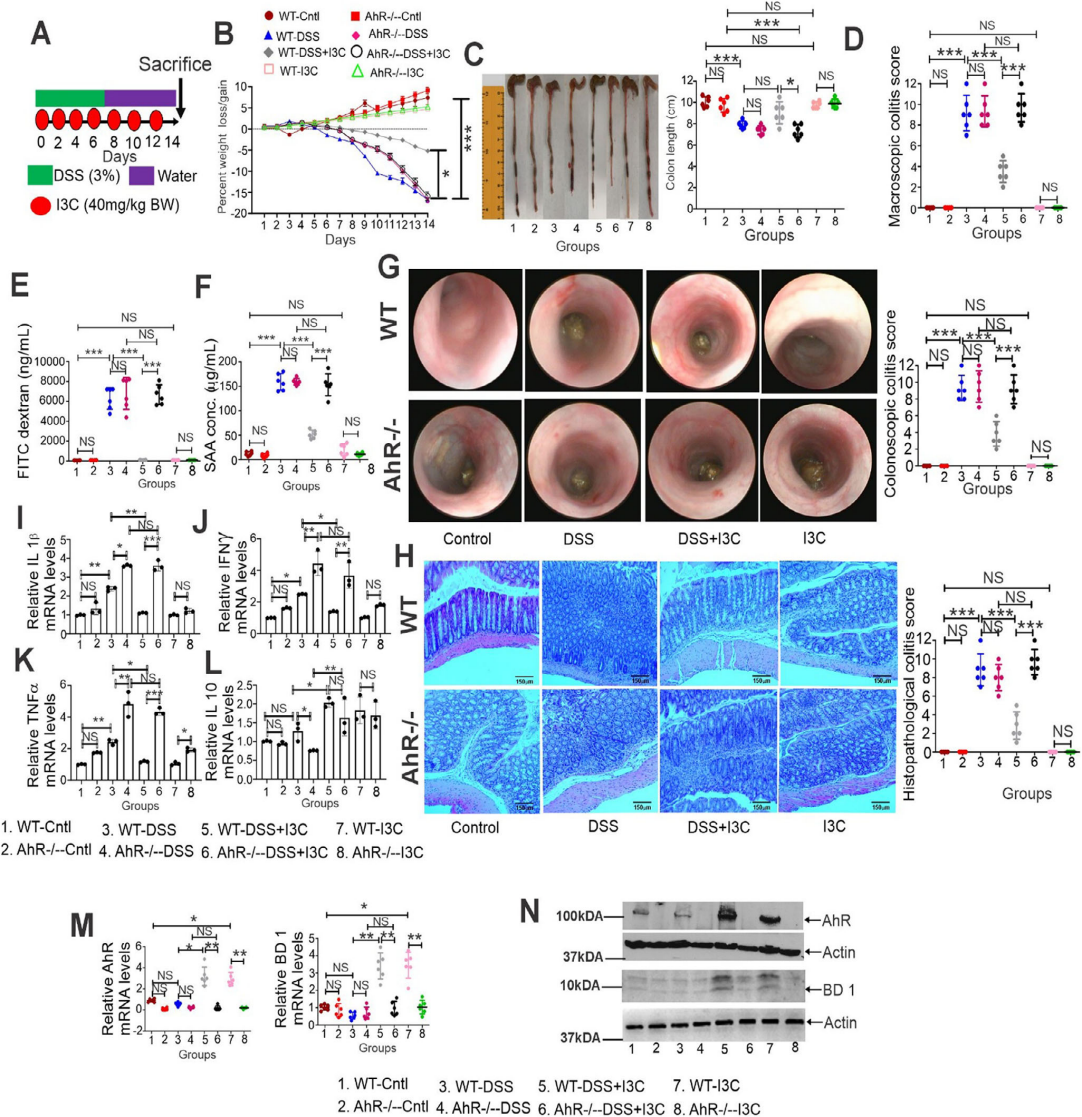
I3C treatment fails to suppress colitis in IEC‐AhR‐deficient mice. A) Experimental design for anti‐CD40‐induced colitis mice model as described in Methods. B–F) Colitis was assessed by B) percent weight loss, C) colon length, D) macroscopic score, E) FITC‐dextran as a measure of gut permeability and damage, F) serum SAA levels. G) Representative colonoscopy images of experimental and control animals (Left panel). Bar graph depicting colonoscopy scores from experimental mice (Right panel) (*n* = 6). H) Representative H&E stains of colons from experimental mice (Left panel). Scale bars: 140 µm (original magnification, × 10). Bar graph depicting histopathological scores of H&E‐stained colons from experimental mice (Right panel) (*n* = 6). I–L) Colonic tissue from control and experimental animals were used to analyze the mRNA expression of I) IL1β, J) IFNγ, K) TNFα, and L) IL10 by real‐time‐PCR (*n* = 3). M) CECs isolated from control and experimental mice were used to analyze the mRNA expression of AhR, and BD‐1 by real time‐PCR (*n* = 6). N) The same as (M), but the protein expression of AhR and BD1 was analyzed by western blot (*n* = 6). Data are displayed as mean ± SD. Significance was determined using 1‐way ANOVA and Tukey's multiple comparisons test; *p < 0.05; **p < 0.01; ***p < 0.001. NS = Not significant.

To investigate the impact of AhR‐mediated induction of mBD‐1 expression on colitis, we generated conditional AhR knockout mice in vil1‐expressing CECs using the cre‐flox system. Next, we induced colitis using DSS in these and wild‐type (WT) mice, and treated them with I3C as shown in **Figure** **5**A.^[^ ^24^ ^]^ We found that AhR conditional KO mice (AhR∆IEC) were more resistant to I3C‐medited attenuation of colitis as indicated by the clinical scores (Figures 5B–H, Supporting Information). Additionally, AhR∆IEC colitis mice showed significantly higher levels of mRNA expression of proinflammatory cytokines IL‐1β (Figure 5I), IFNγ (Figure 5J), and TNFα (Figure 5K) in colonic mucosa when compared to WT mice with colitis. Also, I3C therapy significantly reduced these cytokines in WT colitis mice, but not in AhR∆IEC colitis mice (Figures 5I–K). When we analyzed the expression of anti‐inflammatory cytokine IL‐10, I3C increased its mRNA levels both in WT colitis mice and AhR∆IEC colitis mice thereby suggesting that the ability of I3C to induce IL‐10 was independent of AhR expression on CECs (Figure 5L). Interestingly, treatment of AhR∆IEC colitis mice with I3C did not result in an increase in mBD‐1 mRNA (Figure 5M) and protein (Figure 5N) expression when compared to WT mice with colitis and treated with I3C, in which there was an increase in mBD‐1. These data suggested that BD‐1 was induced through AhR activation on CECs.

Using GSE242891 data set, we further investigated the effect of AhR deletion in CECs on tight junction (TJ) integrity. Carcinoembryonic antigen‐related cell adhesion molecules (CEACAMs) are glycoproteins that are highly expressed in IECs and show altered expression during colitis.^[^ ^25^ ^]^ We found that CEACAMS 1,9,10,15,18,19, etc. were induced by I3C in WT colitis mice but not in AhR∆IEC colitis mice (Figure S10A, Supporting Information). Similarly, we analyzed Claudins, a class of proteins, that comprise the backbone of TJs.^[^ ^26^ ^]^ Our analysis found that some Claudins were downregulated in WT mice with colitis, and I3C treatment increased their expression (Claudins 34c2 and 34b2) (Figure S10A, Supporting Information). In contrast, some Claudins were upregulated in WT mice with colitis and I3C treatment decreased their expression (Claudins 1,2,4,6,7,10,22,34d) (Figure S10A, Supporting Information).

### 2.4 mBD‐1 Plays a Critical Role in the Attenuation of Colitis

**Figure 6:**
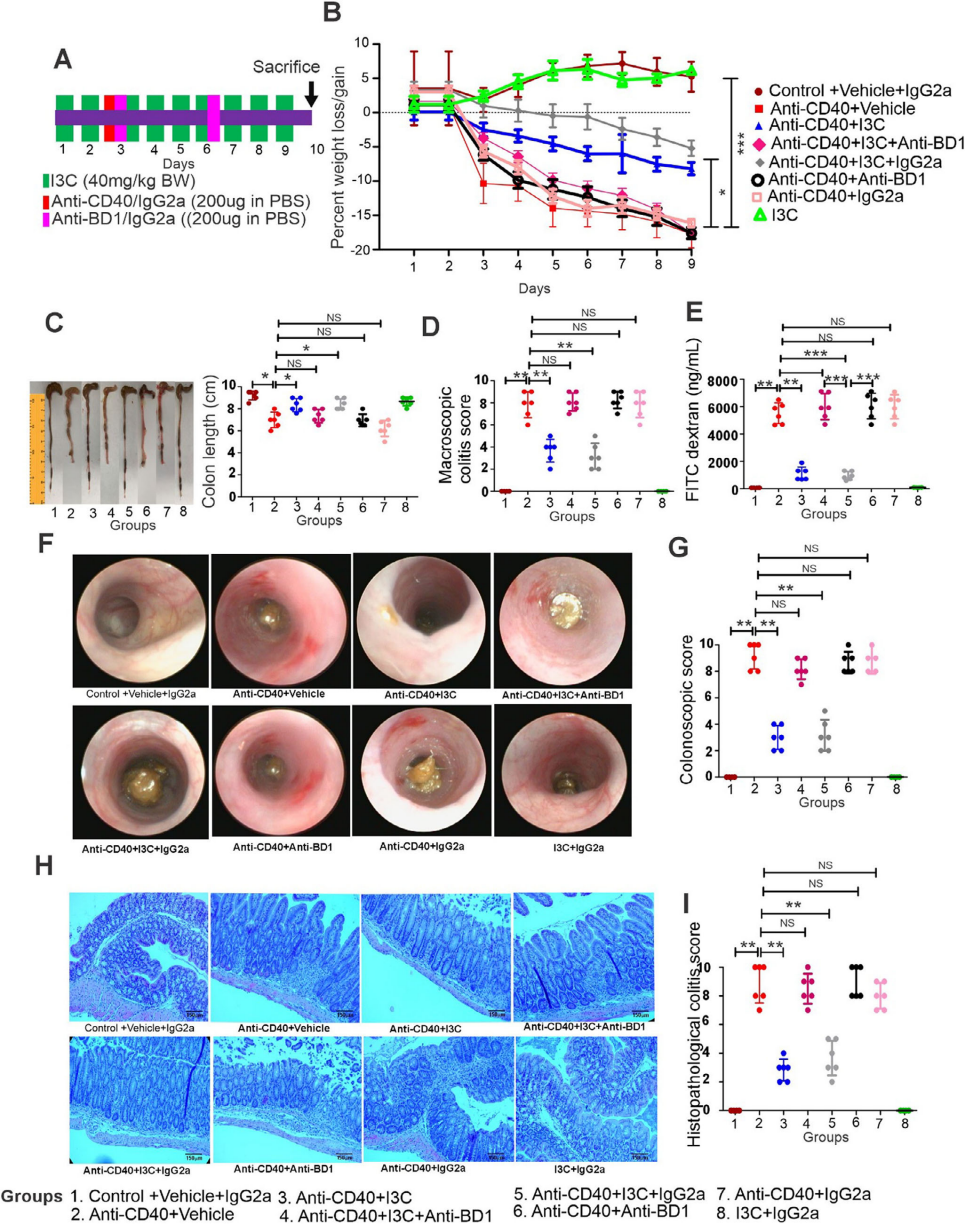
AhR ligand I3C fails to inhibit colitis in mice when BD‐1 is blocked in vivo. A) Experimental design for BD‐1 blocking and anti‐CD40‐induced colitis mice model as described in Methods. B–F) Colitis was assessed by B) percent weight loss, C) colon length, D) macroscopic score, and E) FITC‐dextran levels. F) Representative colonoscopy images of experiments and control animals. G) Bar graph depicting colonoscopy scores from experimental mice (*n* = 6). H) Representative H&E stains of colons from experimental mice (Left panel). Scale bars: 140 µm (original magnification, × 10). I) Bar graph depicting histopathological scores of H&E‐stained colons from experimental mice (Right panel) (*n* = 6). Data are shown as mean ± SD, *n* = 6 and significance were determined using 1‐way ANOVA and Tukey's multiple comparisons test; *p < 0.05; **p < 0.01; ***p < 0.001.

We next investigated whether AhR‐mediated induction of mBD‐1 plays a role in the regulation of colitis. To that end, we administered anti‐BD1 Abs or isotype control Abs (IgG) (200 µg in PBS) twice during colitis mice as shown in **Figure** **6**A to block BD1. The treatment of anti‐CD40 colitis mice with I3C reduced the overall colitis as seen before with reversal of weight loss (Figure 6B), colon length (Figure 6C), macroscopic score (Figure 6D), and gut leakage as evidenced by decreased FITC‐dextran levels (Figure 6E). Furthermore, scoring the colonic mucosa for damage revealed significantly reduced mucosal damage in mice treated with I3C (Figures 6F,G). Histological assessment of colon (Figures 6H,I) showed strong mucosal damage caused by anti‐CD40, characterized by a massive loss of the crypt architecture. In contrast, treatment with I3C prevented this loss of crypts and maintained a normal mucosa, comparable to the naïve mice. Interestingly, in these experiments, when we treated mice with mBD‐1 antibodies, I3C was not able to attenuate anti‐CD40‐mediated colitis (Figures 6B–I). We observed similar results in male mice (Figures S11A–S11I, Supporting Information). In addition, we analyzed the mRNA expression of numerous cytokines such as IL 1β, IL23, IL6, TNFα, and IFNγ in whole colon tissue from control and experimental animals. Our study revealed that colitis animals had higher levels of these cytokines, while I3C therapy dramatically reduced these cytokines (Figures S12A–S12E, Supporting Information). However, blocking BD‐1 in vivo with Abs in colitis animals resulted in elevated levels of different cytokines (Figures S12A–S12E, Supporting Information). Collectively, these findings indicated that mBD‐1 induced following AhR activation plays a critical role in the attenuation of colitis in vivo.

### 2.5 I3C Treatment Reverses Microbial Dysbiosis through AhR‐Dependent Upregulation of mBD‐1 In Vivo

**Figure 7:**
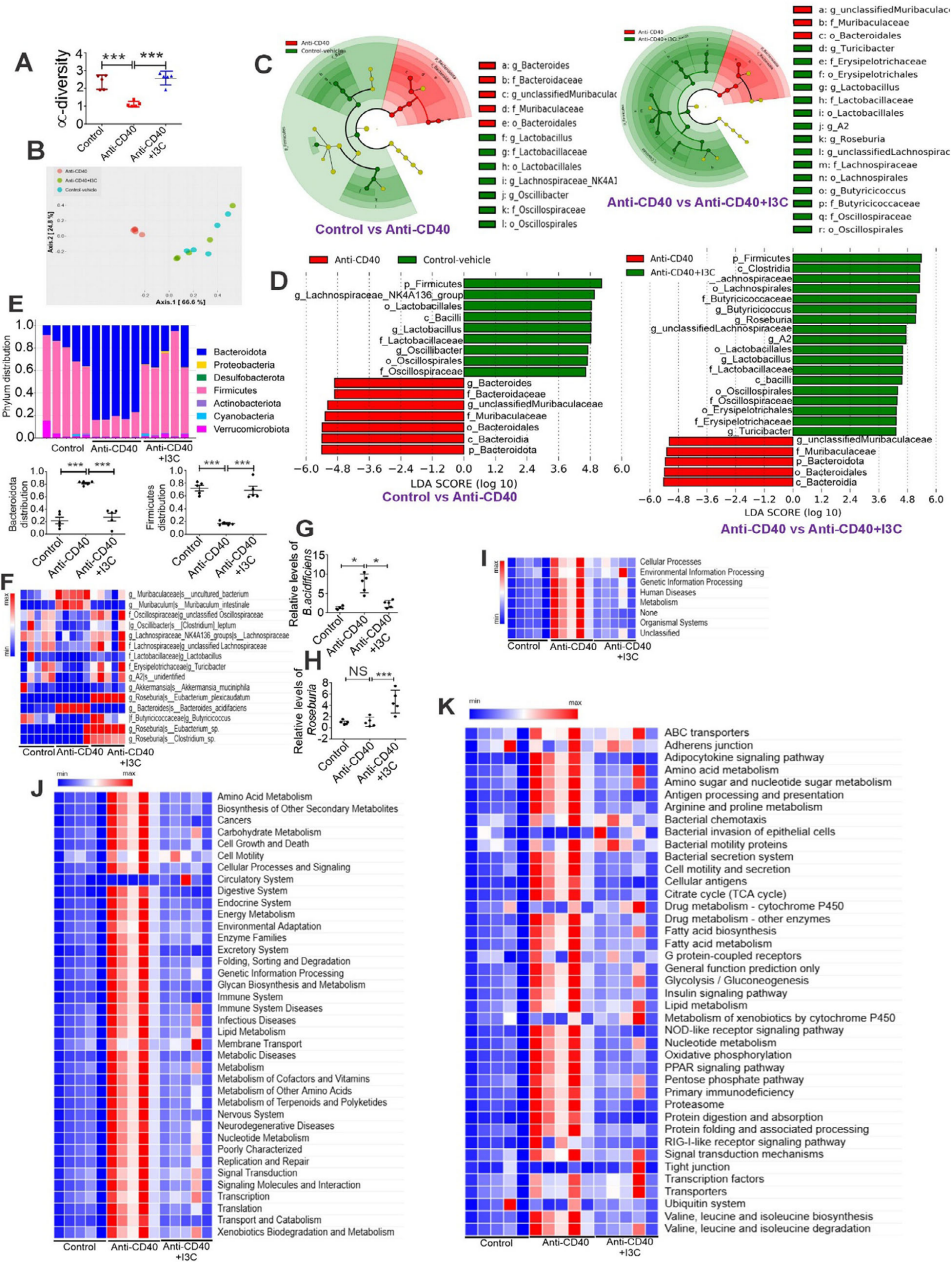
AhR ligand, I3C, alters the colon microbiota composition in anti‐CD40‐induced colitis. 16S rRNA sequencing from the colonic contents of control and experimental animals was performed. Sequenced reads were analyzed using Nephele to determine A) chao1 α diversity and B) β diversity by PCA plot. C) Cladogram and D) LDA score depicting biomarkers between control and anti‐CD40 groups (Left panel)/between anti‐CD40 and anti‐CD40+I3C groups (right panel). E) A detailed overview of phylum distributions in both control and experimental animals. The graph shown in the lower panel illustrates the abundance of Bacteroidota and Firmicutes in control and colitis‐induced mice (*n* = 5). F) The heatmap illustrates a more granular species distribution in both control and experimental animals (*n* = 5). G,H) Real‐time PCR analysis was performed to assess the levels of B. acidifaciens (G) from the Bacteroidota phylum and Roseburia (H) from the Firmicutes phylum in control and experimental animals (*n* = 5). I–K) A heatmap showing the predicted changes in colon microbiota function for control and experimental animals based on KEGG pathway analysis at levels 1, 2, and 3 (I‐Level 1, J‐Level 2, and K‐Level 3) (*n* = 5). Data are shown as mean ± SD; *n* = 5. Significance was determined using 1‐way ANOVA and Tukey's multiple comparisons test; *p < 0.05; ***p < 0.001.

Commensal bacteria or their metabolites have been shown to activate AhR, which reduces microbial translocation and fibrosis in the gut, thus contributing to the restoration of gut homeostasis.^[^ ^7^, ^18^ ^]^ Also, it has been demonstrated that gut microbiota stimulate the expression of antimicrobial peptides such as RegIIIc and certain defensins by the CECs, and that epithelial cell‐produced AMPs are essential for maintaining intestinal homeostasis by controlling microbiota.^[^ ^10^, ^27^ ^]^ We therefore studied the impact of AhR‐mediated induction of mBD‐1 on microbial dysbiosis in colitis mice. The 16S rRNA sequencing from the colon contents of control and colitis animals was performed using the MiSeq platform. Sequenced reads were analyzed using Nephele to determine chao1 α diversity (**Figure** **7**A) and β diversity (Figure 7B) by PCA plot. Anti‐CD40‐induced colitis mice displayed significantly lower chao1 α diversity than controls. However, I3C treatment enhanced the chao1 α diversity in colitis mice. In addition, in terms of β diversity, colonic samples from colitis mice were significantly different between control versus treatment (Anti‐CD40 + I3C) groups (Figure 7B). The results of α and β diversity analysis showed that the microbiome composition of colitis mice treated with I3C was more similar to that of controls than of colitis‐induced animals.

LEfSe was used to identify differentially enriched microbial taxa from the phylum to the genus level in control and colitis mice (Figures 7C,D). Cladogram (Figure 7C) and LDA score (Figure 7D) depicting enriched microbial taxa between control versus Anti‐CD40 colitis groups (left panel) and between Anti‐CD40 versus Anti‐CD40+I3C groups (right panel). Cladogram (Figure 7C) and LDA score (Figure 7D) and Bar plot (Figure 7E) demonstrated that anti‐CD40‐induced colitis mice showed a decrease in many members of the Firmicutes phylum (gram‐positive bacteria) and an increase in the members of Bacteroidia phylum (gram‐negative bacteria) (Figures 7C–E). However, I3C treatment enhanced the many members of the Firmicutes phylum that produce important short‐chain fatty acids (SCFAs) such as butyrate, which keeps the colon healthy. In addition, I3C treatment decreased the members of Bacteroidia phylum (Figures 7C–E) that enhances the production of LPS. We also found that lactobacillus, which plays a protective role against UC, was predominantly found in control mice as well as colitis+I3C mice but were lacking in anti‐CD40 colitis mice (Figures 7C,D).

In our study, we identified differences in the microbiota composition at the species level between control and colitis mice, as presented in heatmaps (Figure 7F). Specifically, we found that the *Bacteroides acidifaciens* and *Muribaculum intestinale* from the Bacteroidia phylum, along with *Acetatifactor muris* from the Firmicutes phylum, were significantly elevated in colitis mice compared to the control group. However, anti‐CD40‐induced colitis mice treated with I3C showed reduced levels of these species (*B. acidifaciens*, *Muribaculum intestinale*, and *Acetatifactor muris*), as depicted in Figure 7F. Interestingly, certain members of the Firmicutes phylum, such as *Eubacterium plexicaudatum*, *Clostridium sp*., and *Eubacterium sp*. from the *Roseburia* genus, as well as *Akkermansia muciniphila* from the Verrucomicrobiota phylum, *Clostridium leptum* from the *Oscillibacter* genus, and members of the *Butyricicoccaceae* genus, were significantly more abundant in the anti‐CD40+I3C‐treated mice compared to the anti‐CD40+Vehicle‐treated mice (Figure 7F). We performed PCR using specific primer for colitis‐associated gram‐negative *Bacteroides acidfaciens* (*B. acidifaciens*) from Bacteroidia (Figure 7G) and beneficial gram‐positive genus *Roseburia* (Figure 7H) from firmicutes phylum.^[^ ^7b^ ^]^ Consistent with results from the Nephele and LefSe data outputs, we found that I3C treatment significantly increased the *Roseburia* and decreased the *B. acidifaciens* in colitis mice. These results suggested that AhR‐mediated production of mBD‐1 (Figures 1C–F and 5M,N) may be responsible for lower levels of gram‐negative bacteria (Figures 7C–H) in colitis mice, because BD‐1 has been demonstrated to eradicate a variety of gram‐negative bacteria.^[^ ^10^, ^11^ ^]^ In addition, reduced abundance of Bacteroidetes in colitis mice treated with I3C facilitates the buildup of potentially many members of firmicutes such as *Roseburia*. Collectively, these data suggested that AhR activation by I3C may attenuate colitis by preventing microbial dysbiosis in colitis mice.

Using 16S rRNA gene amplicon sequencing, we employed PICRUSt2 for KEGG pathway analysis of the intestinal microbiota. Cluster analysis of the KEGG pathways at the first level revealed that the intestinal microbiota in the anti‐CD40‐induced colitis mice primarily exhibited functions related to cellular processes, human diseases, biological systems, metabolism, genetic information processing, and environmental information processing as shown in Figure 7I. Cluster analysis of KEGG pathways at the second level classified gene functions into several distinct pathways. Among these, the intestinal microbiota in the anti‐CD40‐induced colitis mice predominantly exhibited functions associated with several pathways, as shown in Figure 7J. Cluster analysis of KEGG pathways at the third level revealed significant differences in the metabolic profiles of the intestinal microbiota between the experimental groups (Figure 7K). In the anti‐CD40‐induced colitis mice, functions related to carbohydrate metabolism, including the pentose phosphate pathway, amino sugar and nucleotide sugar metabolism, glycolysis and gluconeogenesis, lipid metabolism, bacterial invasion of epithelial cells, and several other pathways highlighted in Figure 7K, were notably enhanced (Figure 7K). However, in the anti‐CD40‐induced colitis mice treated with I3C, these functional profiles were largely reversed (Figure 7I–K).

To investigate if the prevention of dysbiosis by I3C during colitis results in microbiota that promotes AhR or BD‐1 expression, we collected the colonic content microbiota from control, anti‐CD40‐induced colitis, and anti‐CD40+I3C treated mice, and cocultured with MC38 and Caco2 cells for 24 h. Next, we analyzed the mRNA and protein expression of AhR and BD1 (Figures S12F–S12I, Supporting Information). Microbiota from colitis mice significantly downregulated the mRNA (Figures S12F and S12G, Supporting Information) and protein (Figures S12H and S12I, Supporting Information) expression of AhR and BD1 compared to cells cocultured with microbiota population from control mice. However, microbiota from anti‐CD40+I3C treated mice significantly increased the expression of AhR and BD1 compared to cells cocultured with microbiota from colitis mice (Figures S12F–S12I, Supporting Information). These findings demonstrated that I3C‐mediated attenuation of dysbiosis may further help the induction of AhR and BD‐1 in CECs.

**Figure 8:**
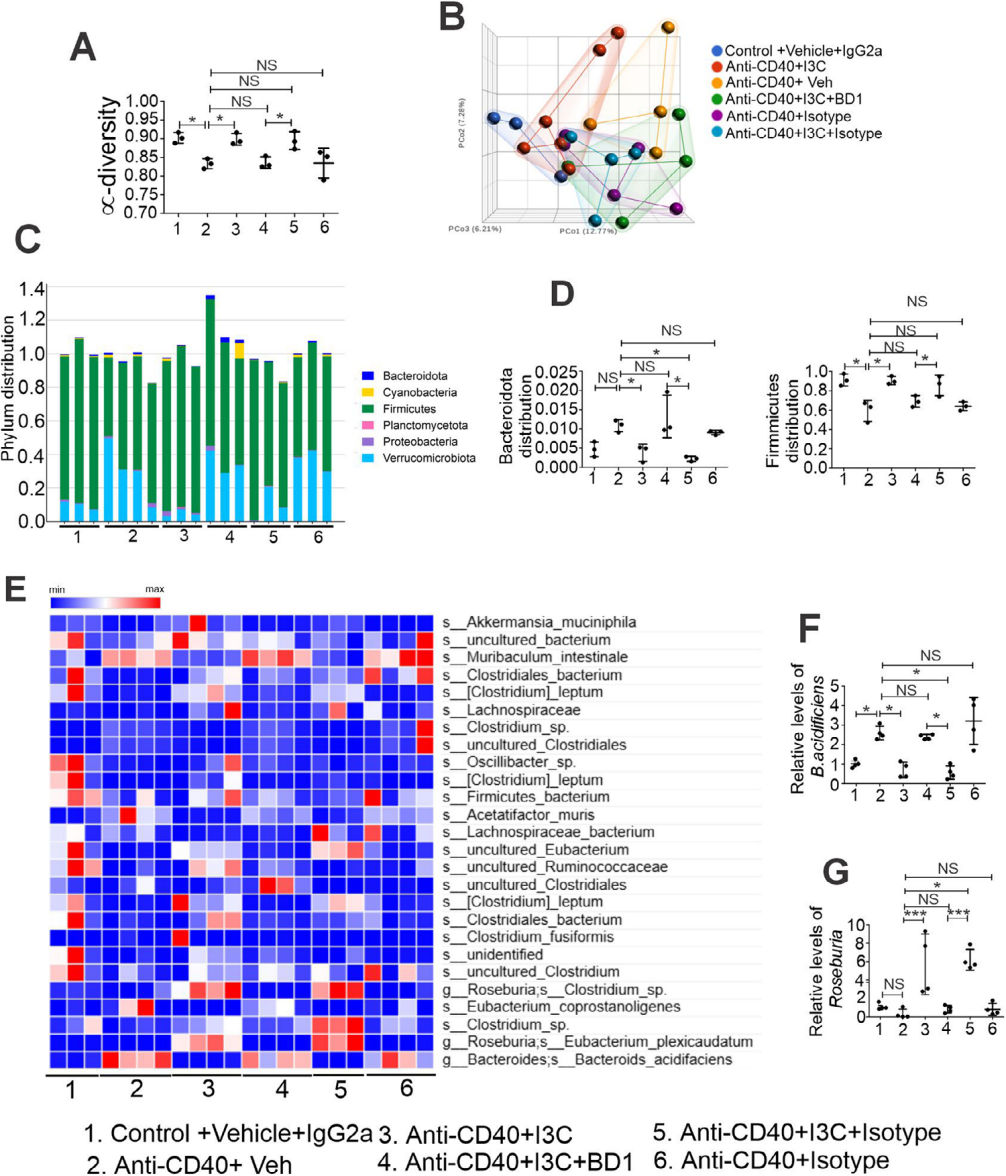
AhR ligand I3C does not alter the colon microbiota composition in anti‐CD40‐induced colitis female mice having BD‐1 defective function. A–G) 16S rRNA sequencing was performed on the colonic contents of control and experimental animals. The sequenced reads were analyzed using Nephele to assess A) Chao1 α diversity and B) β diversity through PCA plotting. C) A comprehensive overview of phylum distributions in both control and experimental groups (*n* = 3). D) The graph in the left panel shows the relative abundance of Bacteroidota and Firmicutes in control and colitis‐induced mice (*n* = 3). E) The heatmap provides a detailed species distribution comparison between control and experimental animals (*n* = 3 or 4). F,G) Real‐time PCR analysis was conducted to validate the levels of *B. acidifaciens* (F) from the Bacteroidota phylum and *Roseburia* (G) from the Firmicutes phylum (*n* = 4). Data are shown as mean ± SD; *n* = 5. Significance was determined using 1‐way ANOVA and Tukey's multiple comparisons test; *p < 0.05; ***p < 0.001.

We next addressed the role of mBD‐1 in colitis‐associated dysbiosis using antibodies against BD1. I3C treatment caused significant changes in gut microbiota with depletion of colitis‐associated gram‐negative bacteria, such as *B. acidfaciens*, and increasing beneficial gram‐positive species (genus *Roseburia*) known to produce butyrate (**Figures** **8**A–G; Figures S13A–S13G, Supporting Information) in anti‐CD40‐induced colitis mice administrated with IgG2a control antibody. However, when the mBD‐1 was blocked using an anti‐BD‐1 antibody, I3C failed to reverse the colitis‐associated dysregulation of gut microbiota and inflammation in female (Figures 8A–G) and male mice (Figures S13A–S13G, Supporting Information). Collectively, our findings demonstrated that AhR‐mediated induction of BD1 plays a crucial role in reversing colitis‐associated dysbiosis.

## 3 Discussion

In the current study, we investigated the role of AhR expressed by the CECs in the regulation of antimicrobial peptide, BD‐1, and consequent effect on microbial flora in the gut as well as colonic inflammation during colitis. Our findings demonstrated for the first time the direct transcriptional upregulation of BD‐1 following AhR activation, and consequently reversal of the colitis‐associated dysbiosis in the gut microbiota and colonic inflammation. The results indicate the role of impaired AhR signaling leading to decreased BD‐1 induction in promoting the development of colitis. The decreased BD‐1 expression in the colon was noted in both human UC patients and murine colitis models. Experiments involving BD‐1 neutralization in vivo demonstrated that AhR‐mediated BD‐1 induction plays a critical role in preventing dysbiosis and colonic inflammation during colitis. Our findings provide a novel link between epithelial cell signaling involving AhR and induction of antimicrobial peptides such as BD‐1 with dysbiosis, colonic inflammation, and colitis.

BD‐1 is an antimicrobial peptide that is widely and constitutively produced by IECs. Virtually, nothing is known about the role of BD‐1 in regulation of IBD in humans or in animal models of colitis. Interestingly, single‐nucleotide polymorphisms in the human BD1 encoding gene have been associated with CD.^[^ ^21^ ^]^ In the current study, we found that UC but not CD patients expressed decreased levels of BD1. This is consistent with the previous studies demonstrating that while these two diseases share some similarities, they have distinct microbiome profiles.^[^ ^28^ ^]^ We discovered that administering I3C to colitis mice significantly increased mBD‐1 through AhR‐mediated DRE signaling pathway. Thus, in mice that failed to express AhR in CECs, I3C failed to induce mBD‐1 and as a result, they developed severe colitis. Also, we observed that the activation of AhR by other AhR ligands such as TCDD, also caused significant upregulation of mBD‐1 and suppressed colitis. We also noted that CECs (such as MC38 and Caco2) deficient in AhR expression were not able to produce BD‐1. Multiple lines of colitis mice models and cell‐based experiments demonstrated that I3C treatment activates AhR, which directly binds to the DREs in the mBD‐1 promoter, leading to the induction of mBD‐1 expression in CECs. Importantly, our studies also demonstrated that AhR‐mediated induction of BD‐1 is involved in the regulation of the gut microbiome because in vivo blocking of BD‐1 prevented AhR ligands from reversing colitis‐associated microbial dysbiosis and colonic inflammation. It was shown that hBD‐1 primarily targets enveloped viruses like HIV‐18, fungi belonging to the Candida species, and Gram‐negative bacteria.^[^ ^11^, ^29^ ^]^ In good agreement, increased AhR‐dependent mBD‐1 expression reversed microbial dysbiosis in this study as evidenced by increased bacteria from firmicutes phylum and decreased gram‐negative bacteria.

Regarding the studies on other antimicrobial peptides, we used the public data set recently published by our group,^[^ ^24^ ^]^ to investigate the differential expression of various AMPs in WT and AhRΔIECs colitis mice, GEO (GSE242891). This analysis revealed that WT colitis mice showed decreased expression of multiple AMPs while treatment of these mice with I3C caused an increase in their expression. Also, AhRΔIECs colitis mice showed a marked decrease in the AMPs and I3C treatment in these mice failed to induce these peptides (Figure S10A, Supporting Information). However, not all antimicrobial peptides showed this pattern.

AhR, which is primarily expressed by epithelial cells and innate immune cells in the gut, plays an important role in the regulation of innate mucosal immunity through the maintenance of delicate balance between proinflammatory and anti‐inflammatory mediators.^[^ ^12^, ^30^ ^]^ Impacts on the maintenance of gut immune cells, such as intraepithelial lymphocytes, innate lymphoid cells (ILCs), Th17 and Treg cells, as well as gut inflammation and barrier activities, are probably how AhR signaling regulates the microbial composition in gut.^[^ ^7^, ^12^, ^14^, ^15^, ^16^, ^17^ ^]^ In UC and CD patients, there is chronic inflammation with a marked accumulation of leukocytes in the gut, which is believed to result from an attempt by the host to curtail the invasion of pathogenic microbiota in the gut.^[^ ^31^ ^]^

In the current study, we noted that AhRΔIEC colitis mice did not exhibit more severe clinical scores of colitis when compared to WT mice with colitis, which is consistent with our previous studies.^[^ ^24^, ^32^ ^]^ However, we did find that these mice had higher levels of inflammatory cytokines in the colon. It is possible that the degree of severity in colitis may depend on whether AhR is deleted only in IECs versus all cells. The data presented in this study on AhRΔIEC colitis mice are consistent with a recent study in which AhR∆IEC colitis mice showed higher levels of certain inflammatory markers but not all—e.g., STAT‐3 and intestinal permeability were not altered in AhR∆IEC mice with colitis when compared to WT mice with colitis.^[^ ^33^ ^]^

The TJ proteins, including occludin and claudins, play a critical role in the maintenance of epithelial barrier integrity. A recent study demonstrated that inducible AhR deletion from IECs worsened intestinal inflammation by damaging the epithelial barrier and modifying microbial species or metabolites, rather than affecting intestinal permeability or TJ integrity.^[^ ^33^ ^]^ However, other studies demonstrated that activation of AhR by alpha‐tocopheryl‐quinone led to an enhanced intestinal TJ barrier function by increasing barrier‐forming claudin‐3 (CLDN3) and reducing channel‐forming CLDN2.^[^ ^34^ ^]^ Such differences can be explained by the fact that the former study used mice deficient in AhR in CECs versus the latter study using an AhR ligand to study the effect on TJ proteins. In the current study, using RNA seq analysis of AhR expression or deletion in CECs, we noted significant alterations in several TJ proteins, especially claudins. However, we did not see a clear pattern of AhR‐dependent or independent expression in TJ proteins and thus, additional studies are necessary. Mucins also play a critical role in protecting the host epithelium in the GI tract. Thus, Muc2 deletion leads to the development of spontaneous colitis in mice.^[^ ^35^ ^]^ We have shown previously that Muc2 was significantly downregulated during colitis while treatment with I3C restored Muc2 expression on IECs.^[^ ^24^ ^]^ Also, this effect was negated in AhRΔIEC mice. Moreover, I3C‐treated colitis WT mice showed increased markers of goblet cells (Tff3 and Clca3b); however, this increase was not altered in AhRΔIEC mice.

Dietary components such as I3C found in cruciferous vegetables, xenobiotics such as TCDD, and microbial metabolites including tryptophan metabolites and SCFAs can act as AhR ligands triggering AhR activation and leading to decreased induction of inflammatory cytokines such as IFNγ, IL‐6, IL‐12, TNF, IL‐7, and IL‐17.^[^ ^31^ ^]^ AhR activation also promotes anti‐inflammatory pathways such as induction of IL‐10, IL‐22, prostaglandin E2, and Foxp3.^[^ ^7^, ^30^ ^]^ The connection between AhR ligands and IBD is also evident from clinical studies demonstrating reduced levels of gut microbiota–derived AhR ligands in such patients.^[^ ^36^ ^]^

Our previous study as well as those from others demonstrated that I3C treatment attenuates colonic inflammation and reveres microbial dysbiosis primarily through induction of IL‐22.^[^ ^7^, ^32^, ^36^ ^]^ Our data supports previous findings by demonstrating that AhR deficiency in CECs leads to loss of protective effects of I3C in the DSS‐induced colitis model, despite restoring anti‐inflammatory cytokines IL10 and IL‐22 production in the colon.^[^ ^24^ ^]^ In this study, we used a single dose of TCDD at 25 µg k^−1^g body weight while for I3C, it was necessary to administer every other day until the end of the experiment. The prolonged versus transient activation of AhR by these two ligands, respectively, may explain the single versus multiple doses necessary to attenuate colitis by these ligands.  TCDD has been shown to activate^[^ ^37^ ^]^ or inhibit^[^ ^38^ ^]^ NF‐κB pathway depending on the dose and tissue targets.  In contrast, studies with I3C have shown that it primarily inhibits NF‐κB pathway.^[^ ^39^ ^]^  Because activation of NF‐kB pathway leads to pro‐inflammatory cytokine induction like TNF‐α, IL‐6, and IL‐1β, we checked if TCDD or I3C when administered alone would induce these cytokines in our model and found that they did not, except TCDD causing a mild increase in IL‐1β.  In fact, TCDD and I3C suppressed these inflammatory cytokines induced during colitis.  Thus, while squelching of AhR by NF‐kB is reported,^[^ ^40^ ^]^ we feel that it was not involved in our study. However, our analysis demonstrates that AhR‐dependent induction of BD1 in CECs may be the key potential mechanism by which AhR can circumvent microbial dysbiosis and colitis. In addition, AhR activation has been shown to govern other critical characteristics in CECs, such as epithelial cell TJ and turnover, which cannot be excluded and need to be investigated further.

Oral or intraperitoneal administration of I3C in rodents leads to similar conversion of I3C into various metabolites, particularly diindolylmethane (DIM) (Reviewed in^[^ ^41^ ^]^). This is also true when you use I3C in cell cultures.^[^ ^41^ ^]^ Thus, we believe that the in vivo and in vitro effects of I3C observed in the current study result from the metabolites such as DIM which is also a potent AhR ligand. As a ligand, I3C should only activate AhR. However, we observed consistent upregulation following treatment of colitis mice or CECs such as MC38 and Caco2 cells with I3C. These findings are consistent with previous reports showing that I3C increased AhR expression in MCF‐7 cells.^[^ ^22^ ^]^ Thus, it is likely that I3C regulates the expression of AhR through additional mechanisms such as miRNA and epigenetic modifications. We have previously shown that AhR ligands mediate differential effects based on miRNA induction.^[^ ^42^ ^]^ Also, others have shown that miRNAs negatively regulate AhR expression in various colitis mouse models.^[^ ^43^ ^]^ Moreover, miRNA such as miRNA‐124 has been shown to regulate the expression of AhR.^[^ ^43b^ ^]^

AhR activation also regulates gut microbiota composition,^[^ ^19^, ^44^ ^]^ which plays a significant role in the regulation of colitis. Multiple studies demonstrated that IBD patients and mice with colitis exhibit significant increases in overall Bacteriodetes and a decrease in Firmicutes at the phylum level.^[^ ^45^ ^]^ Interestingly, individuals with IBD have reduced levels of commensal microbiomes that produce AhR ligands, including *Butyricicoccus*, *Lactobacillus*, and *Roseburia*.^[^ ^6^, ^46^ ^]^ In the current study, we noted a decrease in Firmicutes and an increase in Bacteriodetes in the murine models of colitis which were reversed following administration of AhR ligands. The decreased abundance of Firmicutes phylum may also lead to reduced production of butyrate, a direct ligand for AhR that keeps the colon healthy.^[^ ^7b^ ^]^ Additionally, the increased Bacteroidia phylum observed in our study has been shown to enhance the production of LPS, which triggers chronic inflammation in the gut.^[^ ^7^, ^47^ ^]^ We also observed decreased levels of lactobacillus from Firmicutes phylum, which has been shown to protect the intestinal mucus barrier, epithelial cell barrier, and intestinal immunological barrier from environmental insults.^[^ ^17^, ^48^ ^]^ Importantly, AhR ligands were able to reverse this dysbiosis seen during colitis by inducing BD‐1. However, Blocking BD‐1 in vivo prevents the ability of I3C to attenuate colitis, thereby connecting the role of BD‐1 to microbiome and colonic inflammation. β‐defensin 1 exhibits microbicidal activity predominantly against Gram‐negative bacteria.^[^ ^49^ ^]^ Consistent with these findings, we observed that I3C treatment enhanced AhR‐β‐defensin 1 signaling, which correlated with decreased levels of Bacteroidetes, a group of mostly gram‐negative bacteria that produce LPS. In addition, reduced abundance of Bacteroidetes in colitis mice treated with I3C facilitates the buildup of potentially many members of firmicutes, including *Lactobacillus*, *Roseburia* and *Butyricicoccus*, which produce butyrate and indole derivatives thereby creating a cycle of persistent AhR activation. This is also supported by the current study in which we found that the colonic microbiota from colitis mice when cultured with CECs, significantly downregulated the expression of AhR and BD1 when compared to the controls. We also observed similar findings in colitis mice and CECs treated with DSS. However, no significant changes in AhR mRNA levels were observed between CECs cocultured with microbiota from control mice and those cocultured with colonic microbiota from colitis mice. This suggests that multiple mechanisms could be playing a role including post‐transcriptional mechanisms, such as ubiquitination, phosphorylation, methylation, acetylation, glycosylation, and proteolytic cleavage or AhR repressor (AHRR), which can indirectly down‐regulate AhR which in turn leads to decreased expression of BD‐1. Clearly additional studies are necessary. Interestingly, microbiota from colitis+I3C treated mice significantly increased the expression of AhR and BD1. These findings indicate how I3C‐mediated AhR activation leads to beneficial microbiota that can induce BD‐1. Our results corroborate other studies showing the significance of microbiota‐mediated AhR activation.^[^ ^17^, ^18^, ^50^ ^]^ In fact, numerous investigations have elucidated the pivotal function of AhR in controlling microbial dysbiosis and inflammation through the regulation of intestinal immune cells in the colon.^[^ ^7^, ^12^, ^14^, ^15^, ^16^, ^30^ ^]^ Also, studies have demonstrated that commensal microbiota modulate intestinal mucosa antimicrobial molecules through the AhR/IL‐22/Stat3 signaling pathway.^[^ ^18^ ^]^

mRNA levels of AhR were previously reported to decrease in CD patients, but not in UC patients.^[^ ^51^ ^]^ Therefore, the levels of AhR in IBD patients need to be further studied while taking into account several factors such as cell specificity, active/inactive disease in tissue evaluated, types of treatments given to patients that might impact AhR expression, and other important demographic information (e.g., sex, age). Previous studies have shown that mucosal hBD‐1 expression was decreased in both CD and UC,^[^ ^20^ ^]^ while another study that measured mRNA showed that the levels of hBD‐1 were not significantly different between IBD patients and controls.^[^ ^52^ ^]^ Yet another study noted that hBD‐1 mRNA was detectable in 61% of control and CD and 53% of UC biopsies.^[^ ^10b^ ^]^ Such discrepancies may have resulted from sample size, type of tissue (whole colonic tissue vs CECs), and assays (mRNA, immunohistochemistry) to study hBD‐1. Also, while hBD‐1 is constitutively expressed in the intestine, highest levels are found in CECs.^[^ ^52^ ^]^ Thus, clearly, more studies are needed using CD patients to test if their CECs also express decreased levels of AhR. It is worth noting that genetic variations in the gene encoding hBD‐1 are associated with the risk for CD.^[^ ^21^ ^]^ Such studies are also necessary in all types of IBD. Also, while we showed that neutralizing BD‐1 reverses the action of AhR ligands in terms of preventing colitis‐associated dysbiosis and colonic inflammation, additional studies are needed to test if AhR‐ligand‐driven microbiota can prevent colitis using fecal microbiota transplantation experiments.

## 4 Conclusion

In summary, downregulation of epithelial cell–specific AhR and BD‐1 could promote the survival and interaction of colitis‐causing bacterial species with host epithelial cells. Because AhR and BD‐1 are important tumor suppressors, inhibiting them may also promote colitis‐associated tumorigenic transformation. Interestingly, AhR‐mediated transcriptional upregulation of BD‐1 in CECs suppresses microbiota‐driven inflammation and colitis. Taken together, these findings reveal a new function for activating AhR in upregulating BD‐1, thereby expanding our knowledge of the pathophysiology of human colitis caused by a compromised AhR pathway.

## 5 Experimental Section

### Cell Culture

CECs, MC38 (Murine adenocarcinoma), and Caco2 (Human colorectal adenocarcinoma) were used in this study. Additional details are provided in online supporting information.

### Gene Expression Profiling of Colitis Patients

Transcriptome profiling data of IBD patients, including both CD and UC, were obtained from publicly available repository GEO with accession number GSE36807, GSE20881, and GSE11223. Also a public data set was enrolled to investigate the differential expression of various antimicrobial peptides in WT and AhRΔIECs colitis mice model, from the GEO (GSE242891). Additional details are provided in online supporting information.

### Human Tissue Samples

Deidentified human colonic tissue specimens from patients with severe UC, CD, and control individuals without colitis or CD were obtained from the University of Miami. The study protocols for human subjects were approved by the Institutional Review Board of the University of Miami under IRB ID 20231111. They were analyzed for the expression of AhR and BD‐1 proteins by IHC using AhR (Cat#sc‐133088) from Santa Cruz Biotechnology (Santa Cruz, CA) and BD1 (Cat#PA575666) from Invitrogen. IHC scores were calculated by multiplying the intensity score, which was graded as 0 (negative), 1 (weak), 2 (moderate), or 3 (strong) by the percentage of positive cells.

### Animals

To generate IEC‐specific conditional AhR knockout mice (AhRΔIEC), AhR3.1Bra/J (strain#: 0 06203) mice were crossed with B6.Cg‐Tg(Vil1‐cre)1000Gum/J (strain#: 02 1504) mice, both of which were originally from a C57BL/6 mouse background. Mice were bred in‐house at the animal facilities located at the University of South Carolina School of Medicine. Mice were genotyped by PCR analysis of DNA isolated from tail snips using DNeasy Blood & Tissue Kit (Qiagen, Hilden, Germany) and primers designed by Jackson Laboratories and purchased from IDT Technologies (Coralville, IA). To further confirm AhR cell‐specific deletion in CECs, the mRNA and protein expression of AhR were measured using RT‐PCR and western blot analysis in enriched CD326+ (or EPCAM+) cells from WT, and AhRΔIEC mice colons. Female C57BL/6 and B6.CB17‐Prkdc^scid^/SzJ mice (8‐10 weeks) purchased from the Jackson Laboratory were housed in specific pathogen‐free conditions, under 12‐h light/12‐h dark cycles. All procedures were conducted in accordance with protocols approved by the Association for Assessment and Accreditation of Laboratory Animal Care (AAALAC)–accredited animal facility at the University of South Carolina School of Medicine, under protocol number 2669‐101803‐070723. Mice were given ad libitum access to water and a standard chow diet.

### Induction of Colitis and Treatment with I3C, Neutralization of BD‐1 Effects and Assessment of Colitis Parameters

B6.CB17‐Prkdc^scid^/SzJ mice were used for the anti‐CD40 Ab‐induced colitis model, while female C57BL/6 mice and AhR^ΔIEC^ were used for the DSS‐colitis studies. The previous method was employed to induce anti‐CD40‐ and DSS‐induced colitis.^[^ ^7^, ^23^, ^53^ ^]^ Four groups of mice were used in both the Anti‐CD40‐ and DSS‐induced colitis models. In the Anti‐CD40 model, Group 1 served as the control group and received intraperitoneal (i.p.) injections of 0.05% DMSO/corn oil and Rat IgG2a isotype control (200 µg in PBS). Mice in Group 2 were injected intraperitoneally (i.p.) with the anti‐CD40 monoclonal antibody FGK45 (200 µg in PBS) and also received 0.05% DMSO/corn oil via intraperitoneal injection. For treatment with I3C, mice in Group 3 were pretreated with 100 µL i.p. injections of I3C (40 mg k^−1^g in 0.05% DMSO/corn oil) 48 h prior to the administration of anti‐CD40, and continued every other day until the end of the experiment.^[^ ^7b^ ^]^ Animals in Group 4 received I3C (40 mg k^−1^g in 0.05% DMSO/corn oil) as in Group 3 and Rat IgG2a isotype control (200 µg in PBS) as in Group 1. Single dose of TCDD (25 µg k^−1^g body weight in 100 uL of corn oil) was administered to mice by i.p., injection 24 h before DSS exposure or anti‐CD40 administration as described previously.^[^ ^17b^ ^]^ To inhibit BD‐1, mice were dosed i.p. with anti‐BD‐1 (200 µg in PBS) two times between the experiment, as described in Figure 6A. For DSS‐induced colitis models, Group 1 was served as controls and received i.p. injections of 0.05% DMSO/corn oil as vehicle for I3C treatment, Group 2 received 3%DSS orally in drinking water and i.p. injections of 0.05% DMSO/corn oil as vehicle, Group 3 animals were received I3C (i.p. injections of 40 mg k^−1^g in 0.05% DMSO/corn oil) 1 h after introduction of DSS and continued every other day until completion of the experiment. Group 4 animals received I3C alone as in group 3. The mice were housed in each treatment group in separate cages, thereby preventing coprophagy across the groups. Also, the food and water were provided ad libitum thereby minimizing coprophagy. Blood, colonic contents, and colon tissue samples were obtained at the end of the experiment. After washing with phosphate‐buffered saline, the colon was sliced longitudinally, formalin fixed, and paraffin embedded.

Colitis disease parameters are assessed as described previously.^[^ ^7^, ^54^ ^]^ Weight was measured daily, and colon lengths, at the end of the experiments. Macroscopic colitis scores were determined based on previous reports.^[^ ^54a^ ^]^ The FITC‐dextran assay was used to test in vivo gut permeability.^[^ ^54b^ ^]^ Briefly, mice were administered orally with 4 kD FITC‐dextran (Sigma‐Aldrich) (600 mg k^−1^g) dissolved in 100 µL of PBS. After 4 h, blood was collected from mice by retroorbital bleeding, and FITC‐dextran concentrations were determined using a Biotek Synergy H4 multimode microplate reader with excitation wavelength at 480 nm. SAA levels from serum were measured on day 8 of the anti‐CD40 model and day 10 of the DSS model using a SAA mouse ELISA (Abcam) according to the procedures provided by the manufacturer. Colonoscopy images were captured on day 9 of the anti‐CD40 model and day 13 of the DSS model using a Karl Storz Tele Pack Vet X LED endoscope designed for small animals. The colonoscopy score was assessed as described previously.^[^ ^55^ ^]^ Histological scores were assessed in formalin‐fixed colonic tissue sections stained with hematoxylin and eosin based on a combination of colonic tissue damage and infiltration of inflammatory cells. Images were captured on Discover ECHO Microscopes.

### I3C Treatment of Cells

Briefly, CECs were pretreated with I3C at 0.1, 1, and 10 µm for 2 h and then treated with DSS (0.03%) for an additional 16 h. Cells were then collected after 16 h and analyzed for mRNA and protein expression of AhR and BD‐1 by RT‐PCR and Western blotting respectively.

### Microbial 16S rRNA Gene Analysis

To perform bacterial phylogenetic analysis, microbial 16S rRNA sequencing analysis was performed using genomic DNA isolated from colonic flushes from control and colitis mice as previously described.^[^ ^7^, ^56^ ^]^ The sequenced data collected on the Illumina Miseq were analyzed using the Nephele platform from the National Institute of Allergy and Infectious Diseases (NIAID) Office of Cyber Infrastructure and Computational Biology (OCICB) in Bethesda, Maryland, USA.^[^ ^57^ ^]^ Output files were analyzed using the LefSe Galaxy web application tool developed by the Huttenhower group to determine gut microbial composition.^[^ ^58^ ^]^ PICRUSt2 was utilized to perform KEGG pathway analysis on the intestinal microbiota based on 16S rRNA gene amplicon sequencing data.

### Isolation of CECs

CECs were isolated from control and experimental mice as described previously.^[^ ^59^ ^]^ Briefly, colons were longitudinally excised after the intestinal contents were removed, and mucus was then gently scraped away in sterile 1X PBS. After being cut into 0.5‐cm pieces, the tissue was incubated for 30 min at 37 °C with shaking in sterile 1X HBSS [without Ca2+ and mg2+] containing 3% FBS, 10 mm EDTA, and 5 mm DL‐dithiotreitol [DTT]. After incubation, these solutions with the colon pieces were filtered through a 100‐µm filter. To allow for the sedimentation of debris, the supernatant containing the intra‐epithelial cells fraction was incubated on ice for at least 10 min. The intra‐epithelial cells fraction extracted from the upper portion of the supernatant was washed with PBS and used for flow cytometric, mRNA and protein analysis.

### Bacterial Collection and Coculture Studies

Colonic contents from control, anti‐CD40‐induced colitis and anti‐CD40‐induced colitis mice treated with I3C were collected in RPMI medium and centrifuged at 500 rpm for 5 min. The supernatant was cocultured with MC38 and Caco2 cells for 24 h, and the cells were analyzed the expression of AhR and BD1 at mRNA and protein levels.

### Whole‐Transcriptome Sequencing

A total RNA was purified from CECs using the Qiagen RNA easy kit. As per manufacturer instructions, a total of 100 ng RNA from each sample (control and experimental mice) was analyzed in Illumina HiSeq 2000 using NextSeq 500/550 High Output Kit v2.5 (75 Cycles) as described previously.^[^ ^60^ ^]^ The Illumina Tru‐seq RNA sample prep kit was used for library preparation. Raw sequencing reads (50 bp single‐end) were mapped to mouse genome and the accepted hits were used for assembling transcripts and estimating their abundance using PARTEK. The differentially expressed genes were determined by PARTEK workflow. The heat maps were generated using “ggplot” in R environment. The raw data were normalized to GAPDH using average expression intensity. This data is available in NCBI's GEO database.

### Other Reagents and Methods

Details for antibodies, Vectors, RNAi, chemicals, transfections, generation of the mBD‐1 promoter reporter constructs, generation of stable cell lines, reporter assays, immunofluorescence, Western Blotting, RNA extraction and Quantitative Reverse‐Transcriptase PCR, chromatin immunoprecipitation and primers are provided in online supporting information.

### Study Approval

The ethics committees of the participating hospitals and the University of Miami, Miami, and the Institutional Review Board of the University of Miami approved the protocols for human studies. Animal studies were performed according to the protocols approved by the Association for Assessment and Accreditation of Laboratory Animal Care–accredited (AAALAC‐accredited) animal facility at the University of South Carolina School of Medicine.

### Statistical Analysis

Statistical analysis was performed using GraphPad Prism 7. The immunohistochemical results were analyzed by 2‐tailed Student's *t* test. RT‐PCR data and the densitometric measurements of western blot bands were statistically analyzed by 1‐way ANOVA followed by Tukey's multiple comparison test. Results were shown as mean ± SD (or SE). Results were considered significant if *p* was less than 0.05. In figures, *p  < .05, **p < .01, ***p < .001, ****p < .001, NS = Not significant.

## Conflict of Interest

The authors declare no competing interests.

## Author Contributions

M.P., M.N., and P.N. conceptualized this study, M.P. performed the majority of experiments, analyzed data, and wrote the manuscript. M.P., K.K., A.M., H.H., and S.T., performed animal experiments, flow cytometry analysis, and analyzed data. M.P., X. Y., and A.M., performed western blot, immunofluorescence, and IHC staining. M.P. and M.G. analyzed the IHC data and interpreted results. M.P., X.Y., K.W., and T.C. performed RNA sequencing. A.S. and P.B.B. contributed to colonoscopic imaging from experimental groups. M.P., K.K., and Y.Z. performed 16sRNA sequencing and analyzed data. X.Y. and N.S. helped to prepare reporter constructs, perform luciferase assays and analyzed data together with M.P. P.B.B. and J.L. helped to interpret results. All other computational analyses of RNA sequencing, transcriptome profiling data of IBD patients were performed by R.G., and M.P., M.N., and P.N. were responsible for obtaining all the funding, coordinating the project, and editing the manuscript. All authors read and approved the final manuscript.

## Supporting information



Supporting Information

## Data Availability

The data that support the findings of this study are available on request from the corresponding author. The data are not publicly available due to privacy or ethical restrictions.

## References

[1] W. K. Chatila , H. Walch , J. F. Hechtman , S. M. Moyer , V. Sgambati , D. M. Faleck , A. Srivastava , L. Tang , J. Benhamida , D. Ismailgeci , C. Campos , F. Wu , Q. Chang , E. Vakiani , E. de Stanchina , M. R. Weiser , M. Widmar , R. K. Yantiss , M. A. Shah , A. J. Bass , Z. K. Stadler , L. H. Katz , I. K. Mellinghoff , N. S. Sethi , N. Schultz , K. Ganesh , D. Kelsen , R. Yaeger , Nat. Commun. 2023, 14, 110. 36611031 10.1038/s41467-022-35592-9 PMC9825391

[2] J. J. Rudbaek , M. Agrawal , J. Torres , S. Mehandru , J. F. Colombel , T. Jess , Nat. Rev. Gastroenterol. Hepatol. 2023, 21, 86. 37950021 10.1038/s41575-023-00854-4 PMC11148654

[3] B. Gros , G. G. Kaplan , JAMA, J. Am. Med. Assoc. 2023, 330, 951. 10.1001/jama.2023.15389 37698559

[4] a) J. T. Chang , N. Engl. J. Med. 2020, 383, 2652; 33382932 10.1056/NEJMra2002697

[5] a) R. P. D. Nascimento , A. Machado , J. Galvez , C. B. B. Cazarin , M. R. Marostica Junior , Life Sci. 2020, 258, 118129; 32717271 10.1016/j.lfs.2020.118129

[6] a) W. Frankel , J. Lew , B. Su , A. Bain , D. Klurfeld , E. Einhorn , R. P. MacDermott , J. Rombeau , J. Surg. Res. 1994, 57, 210; 8041140 10.1006/jsre.1994.1133

[7] a) H. R. Alrafas , P. B. Busbee , M. Nagarkatti , P. S. Nagarkatti , J. Leukocyte Biol. 2019, 106, 467; 30897248 10.1002/JLB.3A1218-476RR PMC6863607

[8] K. Gronbach , I. Flade , O. Holst , B. Lindner , H. J. Ruscheweyh , A. Wittmann , S. Menz , A. Schwiertz , P. Adam , B. Stecher , C. Josenhans , S. Suerbaum , A. D. Gruber , A. Kulik , D. Huson , I. B. Autenrieth , J. S. Frick , Gastroenterology 2014, 146, 765. 24269927 10.1053/j.gastro.2013.11.033

[9] a) C. L. Bevins , N. H. Salzman , Nat. Rev. Microbiol. 2011, 9, 356; 21423246 10.1038/nrmicro2546

[10] a) D. A. O'Neil , E. M. Porter , D. Elewaut , G. M. Anderson , L. Eckmann , T. Ganz , M. F. Kagnoff , J. Immunol. 1999, 163, 6718; 10586069

[11] a) R. Bals , M. J. Goldman , J. M. Wilson , Infect. Immun. 1998, 66, 1225; 9488417 10.1128/iai.66.3.1225-1232.1998 PMC108037

[12] V. Rothhammer , F. J. Quintana , Nat. Rev. Immunol. 2019, 19, 184. 30718831 10.1038/s41577-019-0125-8

[13] B. Stockinger , K. Shah , E. Wincent , Nat. Rev. Gastroenterol. Hepatol. 2021, 18, 559. 33742166 10.1038/s41575-021-00430-8 PMC7611426

[14] a) K. Furumatsu , S. Nishiumi , Y. Kawano , M. Ooi , T. Yoshie , Y. Shiomi , H. Kutsumi , H. Ashida , Y. Fujii‐Kuriyama , T. Azuma , M. Yoshida , Dig. Dis. Sci. 2011, 56, 2532; 21374063 10.1007/s10620-011-1643-9

[15] a) F. J. Quintana , A. S. Basso , A. H. Iglesias , T. Korn , M. F. Farez , E. Bettelli , M. Caccamo , M. Oukka , H. L. Weiner , Nature 2008, 453, 65; 18362915 10.1038/nature06880

[16] a) A. Yeste , I. D. Mascanfroni , M. Nadeau , E. J. Burns , A. M. Tukpah , A. Santiago , C. Wu , B. Patel , D. Kumar , F. J. Quintana , Nat. Commun. 2014, 5, 3753; 24796415 10.1038/ncomms4753 PMC4157605

[17] a) T. Takamura , D. Harama , S. Matsuoka , N. Shimokawa , Y. Nakamura , K. Okumura , H. Ogawa , M. Kitamura , A. Nakao , Immunol. Cell Biol. 2010, 88, 685; 20231854 10.1038/icb.2010.35

[18] J. Wang , P. Wang , H. Tian , F. Tian , Y. Zhang , L. Zhang , X. Gao , X. Wang , Innate Immun. 2018, 24, 297. 29976114 10.1177/1753425918785016 PMC6830914

[19] J. J. Hou , A. H. Ma , Y. H. Qin , Front. Cell. Infect. Microbiol. 2023, 13, 1279172. 37942478 10.3389/fcimb.2023.1279172 PMC10628454

[20] J. Wehkamp , J. Harder , M. Weichenthal , O. Mueller , K. R. Herrlinger , K. Fellermann , J. M. Schroeder , E. F. Stange , Inflammatory Bowel Dis. 2003, 9, 215. 10.1097/00054725-200307000-00001 12902844

[21] A. K. Kocsis , P. L. Lakatos , F. Somogyvari , P. Fuszek , J. Papp , S. Fischer , T. Szamosi , L. Lakatos , A. Kovacs , P. Hofner , Y. Mandi , Scand. J. Gastroenterol. 2008, 43, 299. 18938660 10.1080/00365520701682615

[22] M. Ociepa‐Zawal , B. Rubis , M. Lacinski , W. H. Trzeciak , Acta Biochim. Pol. 2007, 54, 113. 17311112

[23] H. H. Uhlig , B. S. McKenzie , S. Hue , C. Thompson , B. Joyce‐Shaikh , R. Stepankova , N. Robinson , S. Buonocore , H. Tlaskalova‐Hogenova , D. J. Cua , F. Powrie , Immunity 2006, 25, 309. 16919486 10.1016/j.immuni.2006.05.017

[24] A. Saxena , C. Mitchell , R. Bogdon , K. Roark , K. Wilson , S. Staley , M. Hailey , M. C. Williams , A. Rutkovsky , P. Nagarkatti , M. Nagarkatti , P. B. Busbee , Int. J. Mol. Sci. 2024, 25, 2404. 38397081 10.3390/ijms25042404 PMC10888603

[25] a) M. Kelleher , R. Singh , C. M. O'Driscoll , S. Melgar , Cytokine Growth Factor Rev. 2019, 47, 21; 31133507 10.1016/j.cytogfr.2019.05.008

[26] S. Cuzic , M. Antolic , A. Ognjenovic , D. Stupin‐Polancec , A. Petrinic Grba , B. Hrvacic , M. Dominis Kramaric , S. Musladin , L. Pozgaj , I. Zlatar , D. Polancec , G. Aralica , M. Banic , M. Urek , B. Mijandrusic Sincic , A. Cubranic , I. Glojnaric , M. Bosnar , V. Erakovic Haber , Front. Pharmacol. 2021, 12, 682614. 34867313 10.3389/fphar.2021.682614 PMC8635807

[27] Y. Zhao , F. Chen , W. Wu , M. Sun , A. J. Bilotta , S. Yao , Y. Xiao , X. Huang , T. D. Eaves‐Pyles , G. Golovko , Y. Fofanov , W. D'Souza , Q. Zhao , Z. Liu , Y. Cong , Mucosal Immunol. 2018, 11, 752. 29411774 10.1038/mi.2017.118 PMC5976519

[28] V. Pascal , M. Pozuelo , N. Borruel , F. Casellas , D. Campos , A. Santiago , X. Martinez , E. Varela , G. Sarrabayrouse , K. Machiels , S. Vermeire , H. Sokol , F. Guarner , C. Manichanh , Gut 2017, 66, 813. 28179361 10.1136/gutjnl-2016-313235 PMC5531220

[29] E. Ricci , S. Malacrida , M. Zanchetta , M. Montagna , C. Giaquinto , A. De Rossi , JAIDS, J. Acquired Immune Defic. Syndr. 2009, 51, 13. 19390326 10.1097/QAI.0b013e31819df249

[30] C. Gutierrez‐Vazquez , F. J. Quintana , Immunity 2018, 48, 19. 29343438 10.1016/j.immuni.2017.12.012 PMC5777317

[31] L. Pernomian , M. Duarte‐Silva , C. R. de Barros Cardoso , Clin. Rev. Allergy Immunol. 2020, 59, 382. 32279195 10.1007/s12016-020-08789-3

[32] C. Mitchell , S. Staley , M. C. Williams , A. Saxena , R. Bogdon , K. Roark , M. Hailey , K. Miranda , W. Becker , N. Dopkins , M. M. Pena , K. M. Hogan , M. Baird , K. Wilson , P. Nagarkatti , M. Nagarkatti , P. B. Busbee , Front. Immunol. 2024, 15, 1444045. 39229279 10.3389/fimmu.2024.1444045 PMC11368719

[33] A. Qazi , S. Comiskey , N. Calzadilla , F. Amin , A. Sharma , E. Khin , N. Holton , C. R. Weber , S. Saksena , A. Kumar , W. A. Alrefai , R. K. Gill , Nutrients 2023, 15, 4980. 38068838 10.3390/nu15234980 PMC10708520

[34] A. S. Ganapathy , K. Saha , A. Wang , P. Arumugam , V. Dharmaprakash , G. Yochum , W. Koltun , M. Nighot , G. Perdew , T. A. Thompson , T. Ma , P. Nighot , Cell Rep. 2023, 42, 112705. 37393618 10.1016/j.celrep.2023.112705 PMC10528852

[35] M. Van der Sluis , B. A. De Koning , A. C. De Bruijn , A. Velcich , J. P. Meijerink , J. B. Van Goudoever , H. A. Buller , J. Dekker , I. Van Seuningen , I. B. Renes , A. W. Einerhand , Gastroenterology 2006, 131, 117. 16831596 10.1053/j.gastro.2006.04.020

[36] B. Lamas , M. L. Richard , V. Leducq , H. P. Pham , M. L. Michel , G. D. Costa , C. Bridonneau , S. Jegou , T. W. Hoffmann , J. M. Natividad , L. Brot , S. Taleb , A. Couturier‐Maillard , I. Nion‐Larmurier , F. Merabtene , P. Seksik , A. Bourrier , J. Cosnes , B. Ryffel , L. Beaugerie , J. M. Launay , P. Langella , R. J. Xavier , H. Sokol , Nat. Med. 2016, 22, 598. 27158904 10.1038/nm.4102 PMC5087285

[37] M. Han , X. Liu , S. Liu , G. Su , X. Fan , J. Chen , Q. Yuan , G. Xu , Toxicol. Lett. 2017, 273, 10. 28302560 10.1016/j.toxlet.2017.03.013

[38] C. E. Ruby , M. Leid , N. I. Kerkvliet , Mol. Pharmacol. 2002, 62, 722. 12181450 10.1124/mol.62.3.722

[39] A. Ahmad , B. Biersack , Y. Li , D. Kong , B. Bao , R. Schobert , S. B. Padhye , F. H. Sarkar , Anti‐Cancer Agents Med. Chem. 2013, 13, 1002. 10.2174/18715206113139990078 PMC3901097 23272910

[40] a) C. F. Vogel , E. M. Khan , P. S. Leung , M. E. Gershwin , W. L. Chang , D. Wu , T. Haarmann‐Stemmann , A. Hoffmann , M. S. Denison , J. Biol. Chem. 2014, 289, 1866; 24302727 10.1074/jbc.M113.505578 PMC3894361

[41] H. L. Bradlow , In Vivo 2008, 22, 441. 18712169

[42] W. H. Neamah , N. P. Singh , H. Alghetaa , O. A. Abdulla , S. Chatterjee , P. B. Busbee , M. Nagarkatti , P. Nagarkatti , J. Immunol. 2019, 203, 1830. 31492743 10.4049/jimmunol.1900291 PMC6755129

[43] a) A. M. Alzahrani , H. Hanieh , H. M. Ibrahim , O. Mohafez , T. Shehata , M. Bani Ismail , M. Alfwuaires , Int. Immunopharmacol. 2017, 52, 342; 29017096 10.1016/j.intimp.2017.09.015

[44] A. Korecka , A. Dona , S. Lahiri , A. J. Tett , M. Al‐Asmakh , V. Braniste , R. D'Arienzo , A. Abbaspour , N. Reichardt , Y. Fujii‐Kuriyama , J. Rafter , A. Narbad , E. Holmes , J. Nicholson , V. Arulampalam , S. Pettersson , npj Biofilms Microbiomes 2016, 2, 16014. 28721249 10.1038/npjbiofilms.2016.14 PMC5515264

[45] a) D. N. Frank , A. L. St Amand , R. A. Feldman , E. C. Boedeker , N. Harpaz , N. R. Pace , Proc. Natl. Acad. Sci. USA 2007, 104, 13780; 17699621 10.1073/pnas.0706625104 PMC1959459

[46] a) F. Imhann , A. Vich Vila , M. J. Bonder , J. Fu , D. Gevers , M. C. Visschedijk , L. M. Spekhorst , R. Alberts , L. Franke , H. M. van Dullemen , R. W. F. Ter Steege , C. Huttenhower , G. Dijkstra , R. J. Xavier , E. A. M. Festen , C. Wijmenga , A. Zhernakova , R. K. Weersma , Gut 2018, 67, 108; 27802154 10.1136/gutjnl-2016-312135 PMC5699972

[47] F. Di Lorenzo , C. De Castro , A. Silipo , A. Molinaro , FEMS Microbiol. Rev. 2019, 43, 257. 30649292 10.1093/femsre/fuz002

[48] C. Li , K. Peng , S. Xiao , Y. Long , Q. Yu , Cell Death Discovery 2023, 9, 361. 37773196 10.1038/s41420-023-01666-w PMC10541886

[49] P. K. Singh , H. P. Jia , K. Wiles , J. Hesselberth , L. Liu , B. A. Conway , E. P. Greenberg , E. V. Valore , M. J. Welsh , T. Ganz , B. F. Tack , P. B. McCray Jr. , Proc. Natl. Acad. Sci. USA 1998, 95, 14961. 9843998 10.1073/pnas.95.25.14961 PMC24558

[50] a) A. Agus , J. Planchais , H. Sokol , Cell Host Microbe 2018, 23, 716; 29902437 10.1016/j.chom.2018.05.003

[51] I. Monteleone , A. Rizzo , M. Sarra , G. Sica , P. Sileri , L. Biancone , T. T. MacDonald , F. Pallone , G. Monteleone , Gastroenterology 2011, 141, 237. 21600206 10.1053/j.gastro.2011.04.007

[52] A. Fahlgren , S. Hammarstrom , A. Danielsson , M. L. Hammarstrom , Clin. Exp. Immunol. 2003, 131, 90. 12519391 10.1046/j.1365-2249.2003.02035.x PMC1808590

[53] U. P. Singh , N. P. Singh , B. Singh , L. J. Hofseth , R. L. Price , M. Nagarkatti , P. S. Nagarkatti , J. Pharmacol. Exp. Ther. 2010, 332, 829. 19940103 10.1124/jpet.109.160838 PMC2835444

[54] a) M. A. Engel , C. A. Kellermann , G. Burnat , E. G. Hahn , T. Rau , P. C. Konturek , J. Physiol. Pharmacol. 2010, 61, 89; 20228420

[55] T. Kodani , A. Rodriguez‐Palacios , D. Corridoni , L. Lopetuso , L. Di Martino , B. Marks , J. Pizarro , T. Pizarro , A. Chak , F. Cominelli , J Vis Exp 2013, 80, e50843. 10.3791/50843 PMC3940705 24193215

[56] K. N. Chitrala , H. Guan , N. P. Singh , B. Busbee , A. Gandy , P. Mehrpouya‐Bahrami , M. S. Ganewatta , C. Tang , S. Chatterjee , P. Nagarkatti , M. Nagarkatti , Eur. J. Immunol. 2017, 47, 1188. 28543188 10.1002/eji.201646792 PMC5704912

[57] N. Weber , D. Liou , J. Dommer , P. MacMenamin , M. Quinones , I. Misner , A. J. Oler , J. Wan , L. Kim , M. C. McCarthy , S. Ezeji , K. Noble , D. E. Hurt , Bioinformatics 2018, 34, 1411. 29028892 10.1093/bioinformatics/btx617 PMC5905584

[58] N. Segata , J. Izard , L. Waldron , D. Gevers , L. Miropolsky , W. S. Garrett , C. Huttenhower , Genome Biol. 2011, 12, R60. 21702898 10.1186/gb-2011-12-6-r60 PMC3218848

[59] W. Becker , H. R. Alrafas , P. B. Busbee , M. D. Walla , K. Wilson , K. Miranda , G. Cai , V. Putluri , N. Putluri , M. Nagarkatti , P. S. Nagarkatti , J Crohns Colitis 2021, 15, 1032. 33331878 10.1093/ecco-jcc/jjaa253 PMC8218712

[60] M. Bam , X. Yang , E. E. Zumbrun , J. P. Ginsberg , Q. Leyden , J. Zhang , P. S. Nagarkatti , M. Nagarkatti , Transl. Psychiatry 2017, 7, 1222. 10.1038/tp.2017.185 PMC5611749 28850112

