# Supplementary material for: AhR‐Dependent Induction of β‐Defensin 1 in Colonic Epithelial Cells Regulates Cross‐Talk between Gut Microbiota and Immune Response Leading to Attenuation of Colitis
Source: Adv Sci (Weinh). 2025 May 23;12(25):2416324. doi: 10.1002/advs.202416324 (PMC12225009; doi:10.1002/advs.202416324)

**AhR-Dependent Induction of β-Defensin 1 in Colonic Epithelial Cells Regulates Cross-Talk Between Gut Microbiota and Immune Response Leading to Attenuation of Colitis**

[Manikandan Palrasu](javascript:;),^1^ Amarnath Marudamuthu,^1^ [Khadija Kakar](javascript:;),^1^  Hamida Hamida,^1^ Shruthi Thada,^1^ Rohan Gupta,^1^ [Kiesha Wilson](javascript:;),^1^ Taylor Carter,^1^ Yin Zhong,^1^ Archana Saxena,^1^ [Xiaoming Yang](javascript:;),^1^ [Narendra Singh](javascript:;),^1^ [Philip Brandon Busbee](javascript:;),^1^ Jie Li^2^, Monica Garcia-Buitrago^3^, [Prakash Nagarkatti](javascript:;),^1^ [Mitzi Nagarkatti](javascript:;),^1*^

# ^1^Department of Pathology, Microbiology and Immunology, University of South Carolina School of Medicine, Columbia, South Carolina, USA.

# ^2^Department of Chemistry and Biochemistry, University of South Carolina, Columbia, South Carolina 29208, USA.

^3^Department of Pathology, University of Miami Miller School of Medicine, Miami, Florida, 33136, USA.

^*^Corresponding Author:

**Mitzi Nagarkatti, Ph.D**

SmartState Endowed Chair, Center for Cancer Drug Discovery

Chair, Department of Pathology, Microbiology, and Immunology
University of South Carolina School of Medicine Columbia

Basic Science Bldg 1, Rm C-26

Columbia, SC, 29208, USA

Phone: 803-216-3404

Email: [mitzi.nagarkatti@uscmed.sc.edu](mailto:mitzi.nagarkatti@uscmed.sc.edu)


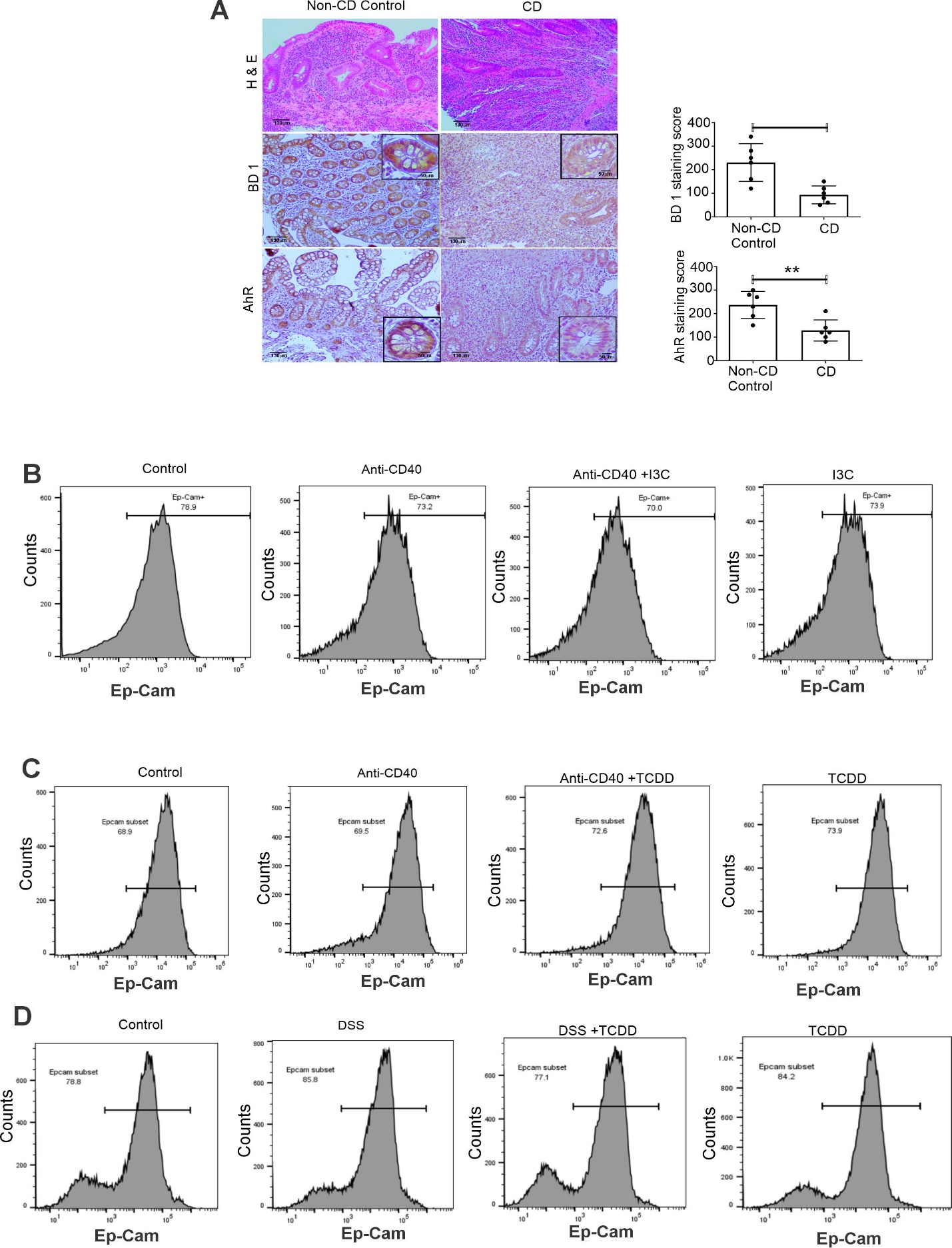

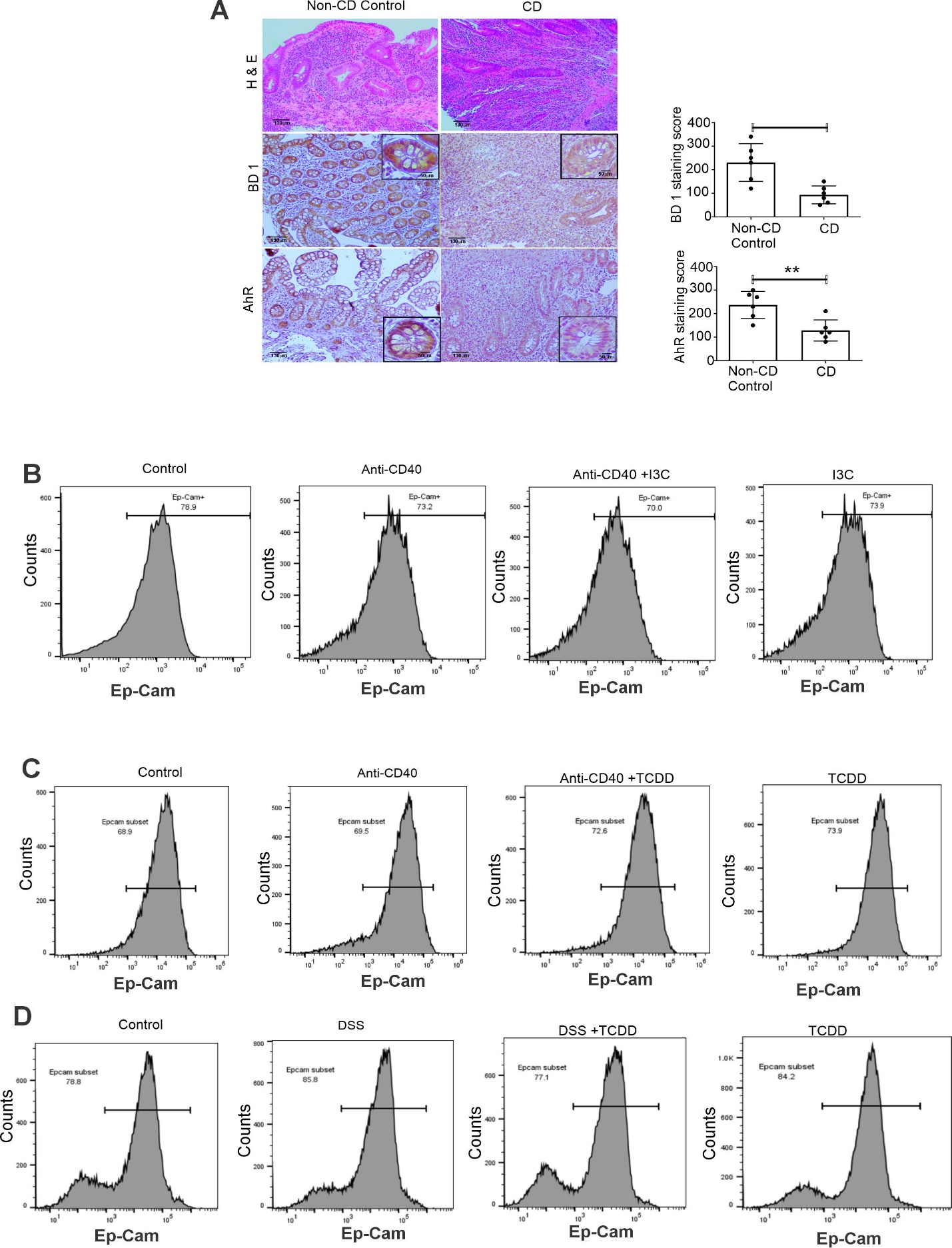


**Figure S1.** (A) Representative immunohistochemical (IHC) staining of AhR and hBD-1 proteins in colonic biopsies from non-CD controls and CD patients is shown. Insets provide magnified views at ×40 magnification, with scale bars representing 50 μm. Dot plots display IHC scores for the expression of the indicated proteins (n = 12; 6 samples per group). Data were analyzed using unpaired 2-tailed t-test; Data are displayed as mean ± SD. ***p* < 0.01. (B) CECs isolated from control and anti-CD40 Ab-induced colitis mice were identified by flow cytometric analysis using the cell surface marker EPCAM. (C) The same as (A), but the treatment was TCDD. (D) The same as (C), but the colitis was induced by DSS.


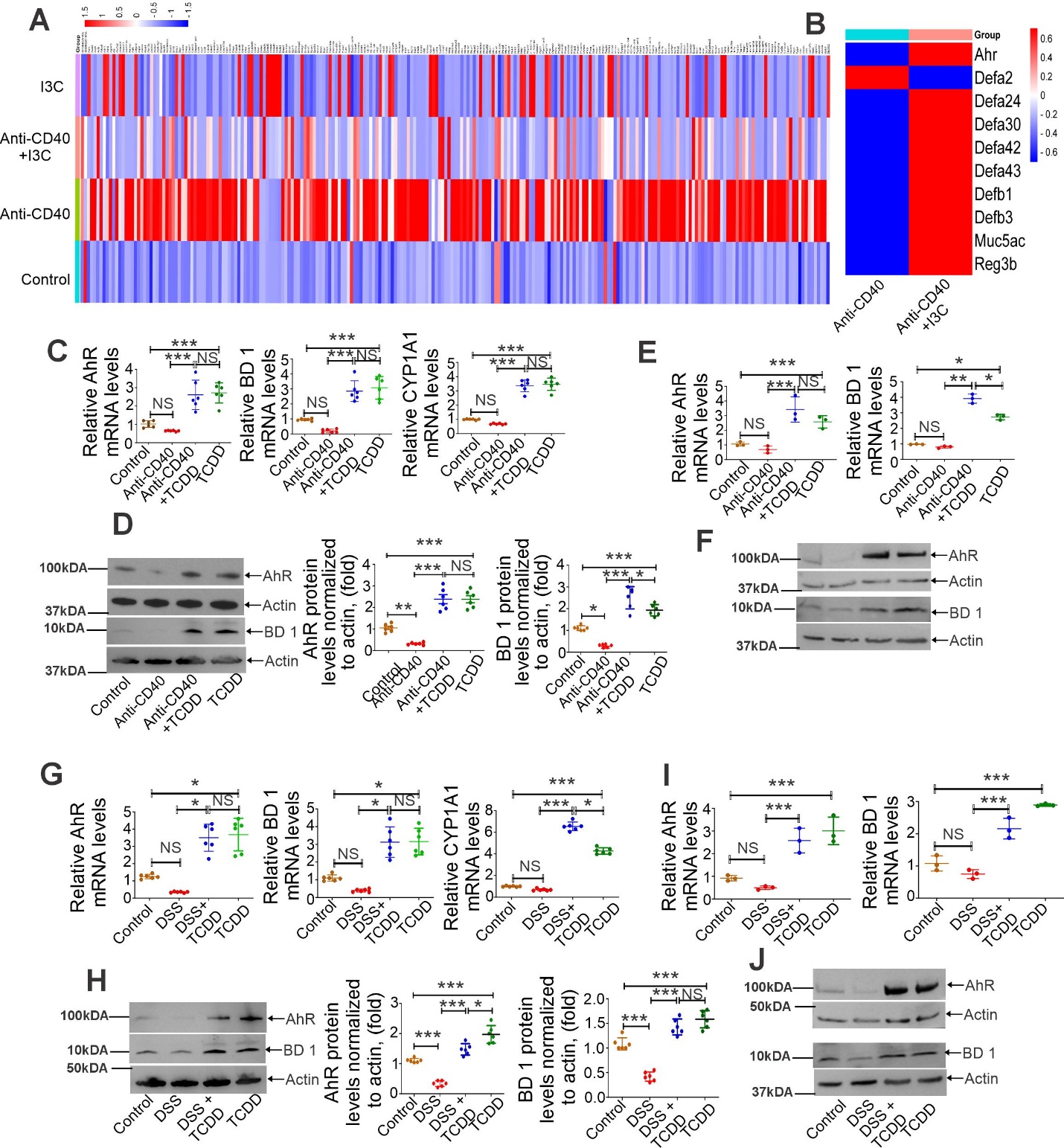


**Figure S2.** TCDD induces AhR and BD-1 in colonic epithelial cells from colitis mice. (A) Transcriptome analysis of CECs from control and anti-CD40-induced colitis mice model. Heatmap using Ward's hierarchical clustering shows two-fold variations in many genes among different groups. The raw data were normalized to GAPDH using average expression intensity. (B) Heatmap depicting dysregulated antimicrobial peptides and other genes expressed as fold changes when compared to vehicle alone. (C, D) mRNA (C) and protein (D) of AHR, and BD-1 in anti-CD40-induced colitis model (n=6). (E,F) The same as (C and D), but mRNA (E) and protein (F) of AHR, and BD-1 were analyzed in the intestinal epithelial cells (n=3). (G, H) mRNA (E) and protein (F) of AHR, and BD-1 in CECs from DSS-induced colitis model (n=6). (I, J) The same as (G and H), but mRNA (I) and protein (J) of AHR, and BD-1 were analyzed in the intestinal epithelial cells (n=3). The graph panels for western blot show quantification of AhR and BD-1 protein by densitometry, normalized to actin. Data are shown as mean ± SEM, and significance was determined using 1-way ANOVA and Tukey’s multiple comparisons test; *p < 0.05; **p < 0.01; ***p< 0.001. NS=Not significant.


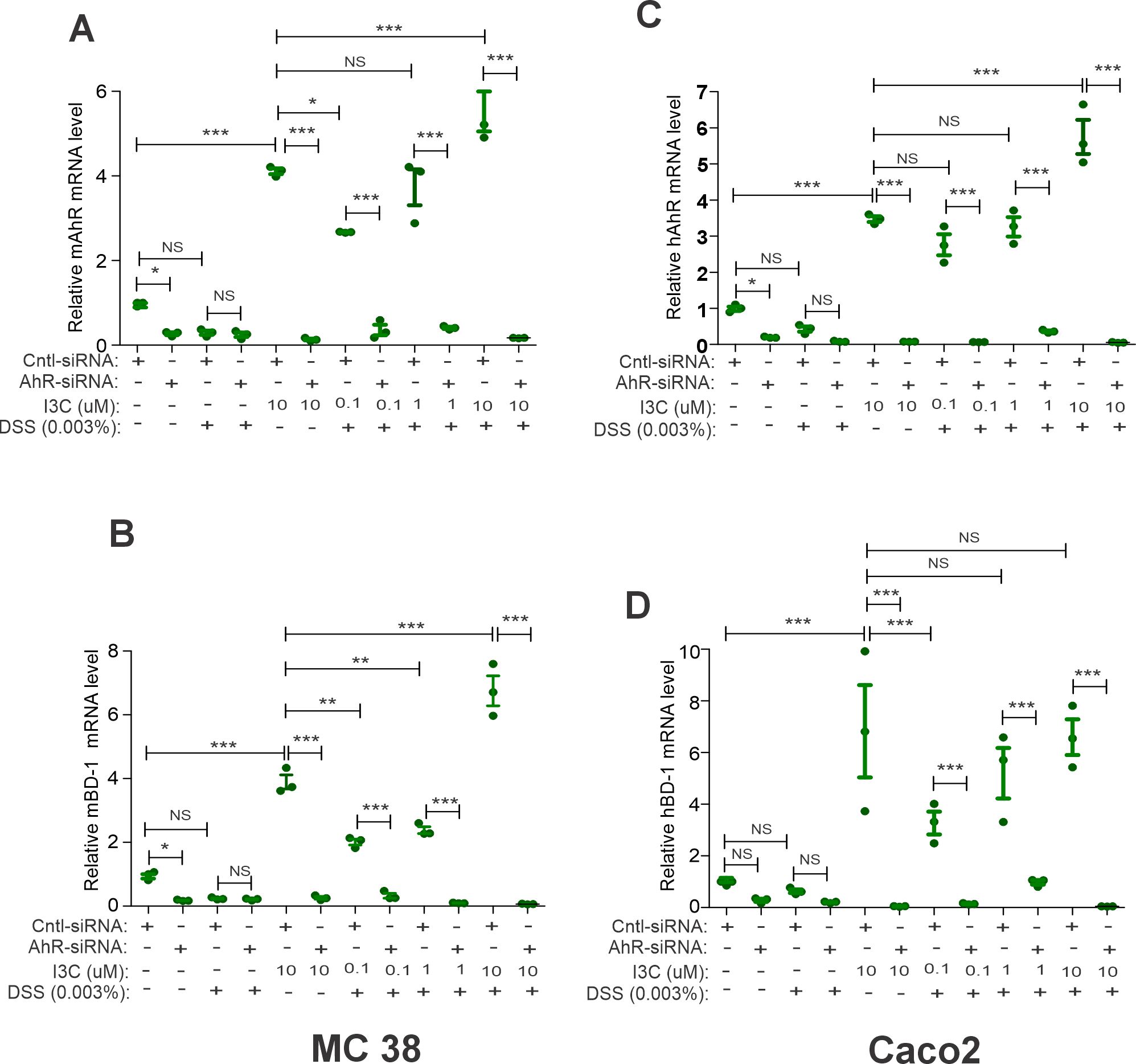
**Figure S3.** Transient knockdown of AhR downregulates BD-1 expression at mRNA level in CECs. MC38 cells transiently transfected with AhR siRNA or control siRNA were pretreated with I3C, at indicated concentrations, and then treated with DSS (0.03%) for an additional 16 hours. The mRNA of AhR (A) and BD-1 (B) was analyzed by Real-time PCR. (C, D) The same as A and B, but Caco2 cells were used. Data are shown as mean ± SEM, and significance was determined using 1-way ANOVA and Tukey’s multiple comparisons test; **p < 0.01; **p < 0.005; ***p < 0.001.


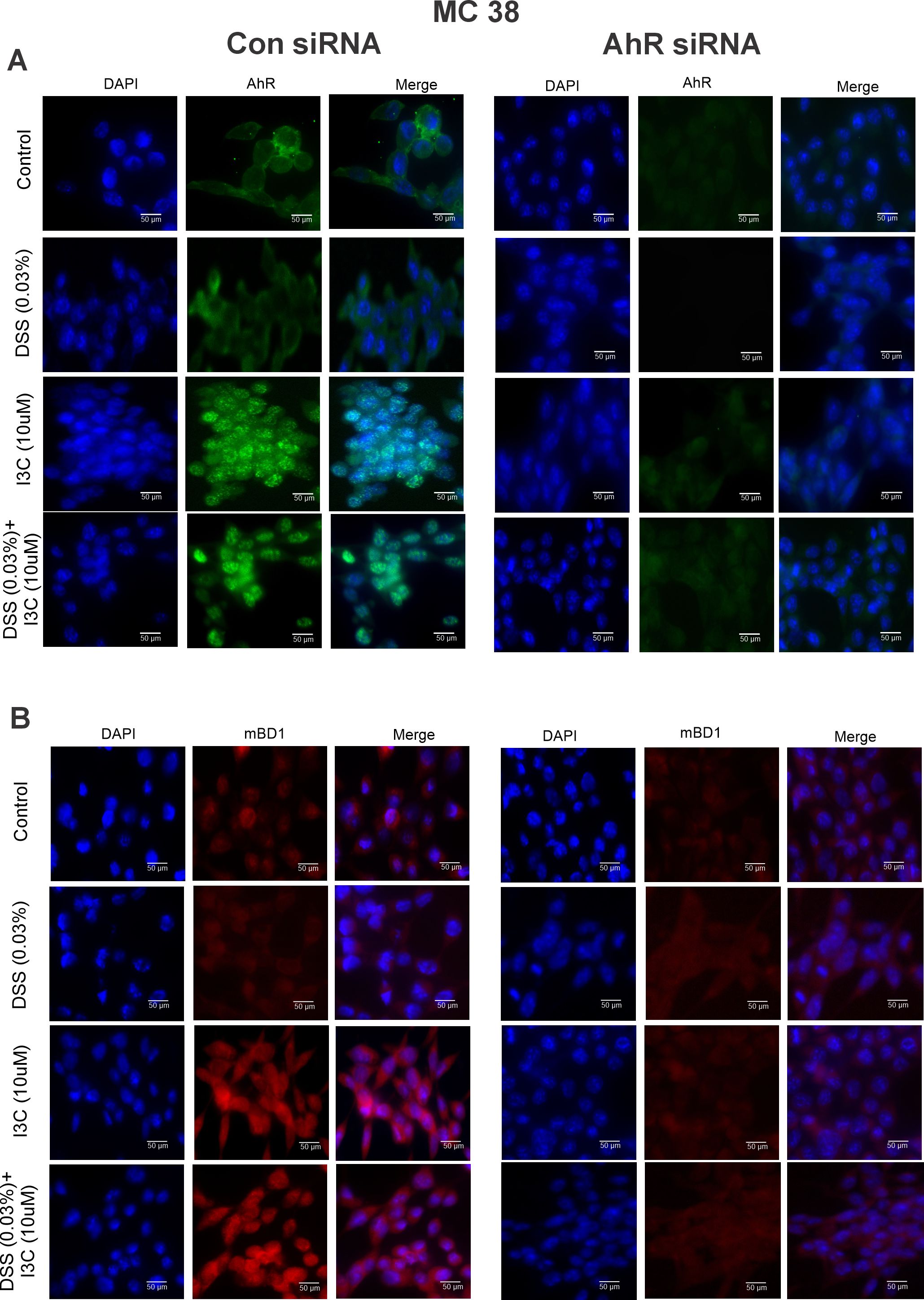


**Figure S4.** Transient knockdown of AhR downregulates BD-1 expression at protein level in CECs. MC38 cells transfected with AhR siRNA or control siRNA were pretreated with I3C, at indicated concentrations, and then treated with DSS (0.03%) for an additional 16 hours. The expression of AhR (A) and BD-1 (B) proteins was analyzed by immunofluorescence staining.


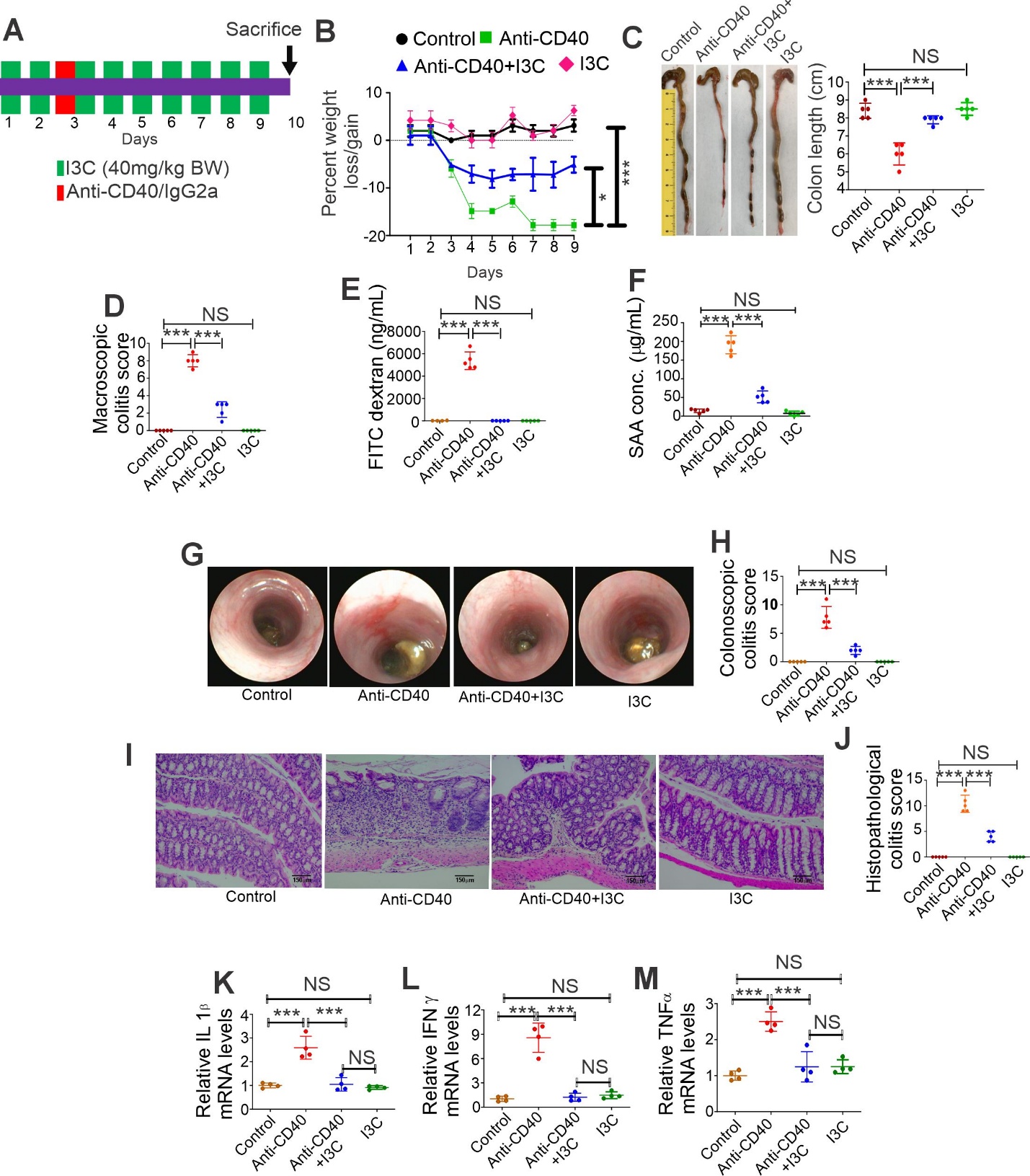


**Figure S5.** I3C treatment leads to attenuation of anti-CD40 Ab-induced colitis. (A) Experimental design for anti-CD40-induced colitis in mice as described in Methods. (B–F) Colitis was assessed by percent weight loss (B), colon length (C), macroscopic score (D), FITC-dextran as a measure of gut permeability and damage (E), and serum SAA levels (F). (G) Representative colonoscopy images of the experimental and control animals. (H) Bar graph depicting colonoscopy scores from experimental mice. (**I**) Representative H&E stains of colons from experimental mice (*n* = 5). Scale bars: 150 μm (original magnification, ×10). (J) Bar graph depicting histopathological scores of H&E-stained colons from experimental mice (*n* = 5). (K-M) Colonic tissue from control and experimental animals were used to analyze the mRNA expression of IL1β (K), IFNγ (L), and TNFα (M) by real-time-PCR (n=4). Data are displayed as mean ± SEM. Significance was determined using 1-way ANOVA and Tukey’s multiple comparisons test; ****p* < 0.001. NS=Not significant.


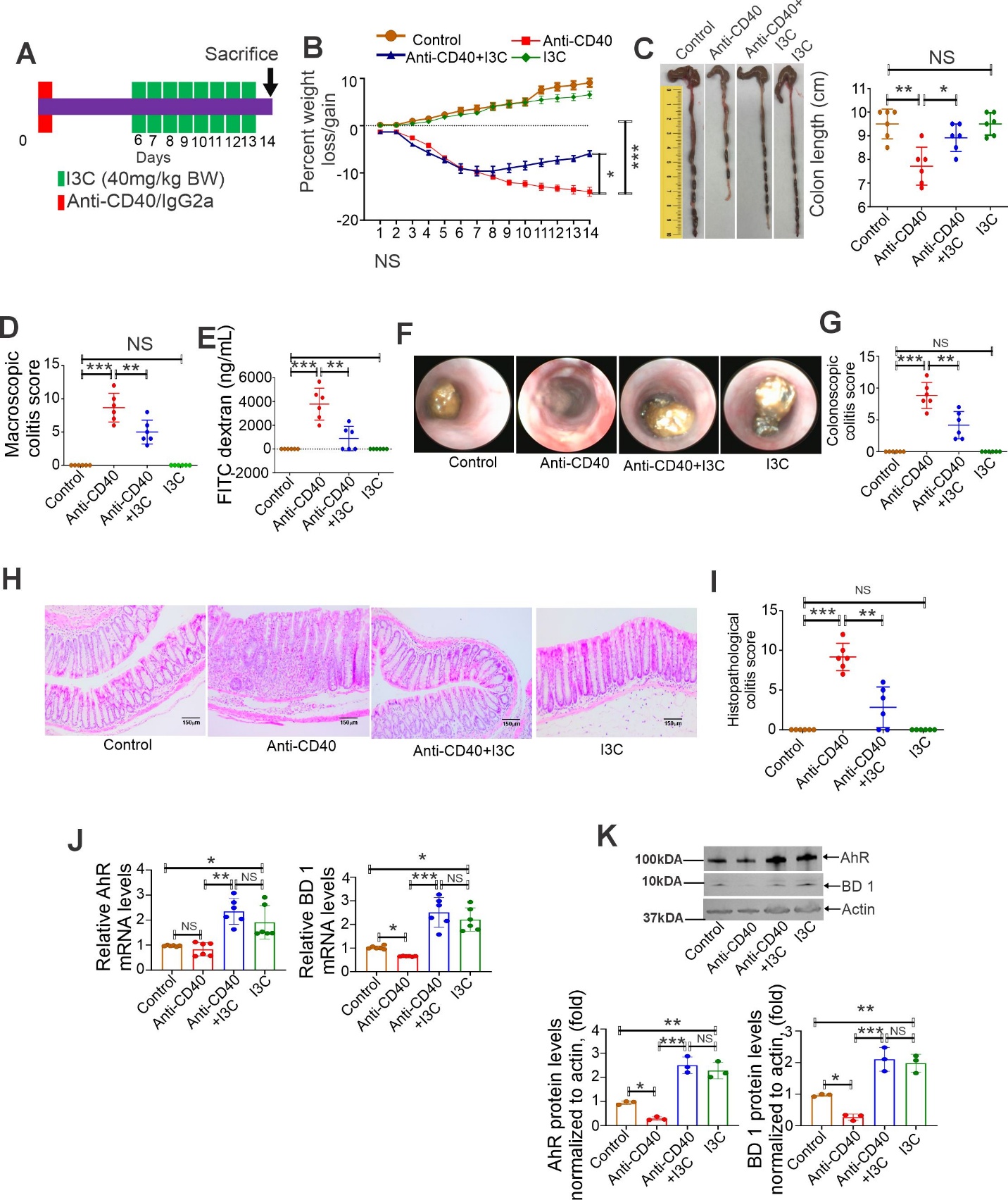
**Figure S6.** I3C posttreatment leads to attenuation of anti-CD40 Ab-induced colitis. (A) Experimental design for anti-CD40-induced colitis and post-treatment of I3C in colitis mice. (B-E) Colitis was assessed by percent weight loss (B), colon length (C), macroscopic score (D), and FITC-dextran as a measure of gut permeability and damage (E), (F,G) Representative colonoscopy images (F) and colonoscopy scores (G) of the experimental and control animals. (H,I) Representative H&E stains of colons (H) and Bar graph (I) depicting histopathological scores from experimental mice (*n* = 6). Scale bars: 150 μm (original magnification, ×10). (J, K) The mRNA (J; n=6) and protein (K; n=3) of AhR, and BD1 in anti-CD40-induced colitis model with or without post-treatment with I3C. Data are displayed as mean ± SEM. Significance was determined using 1-way ANOVA and Tukey’s multiple comparisons test; *p < 0.05; **p < 0.01; ***p < 0.001. NS=Not significant.


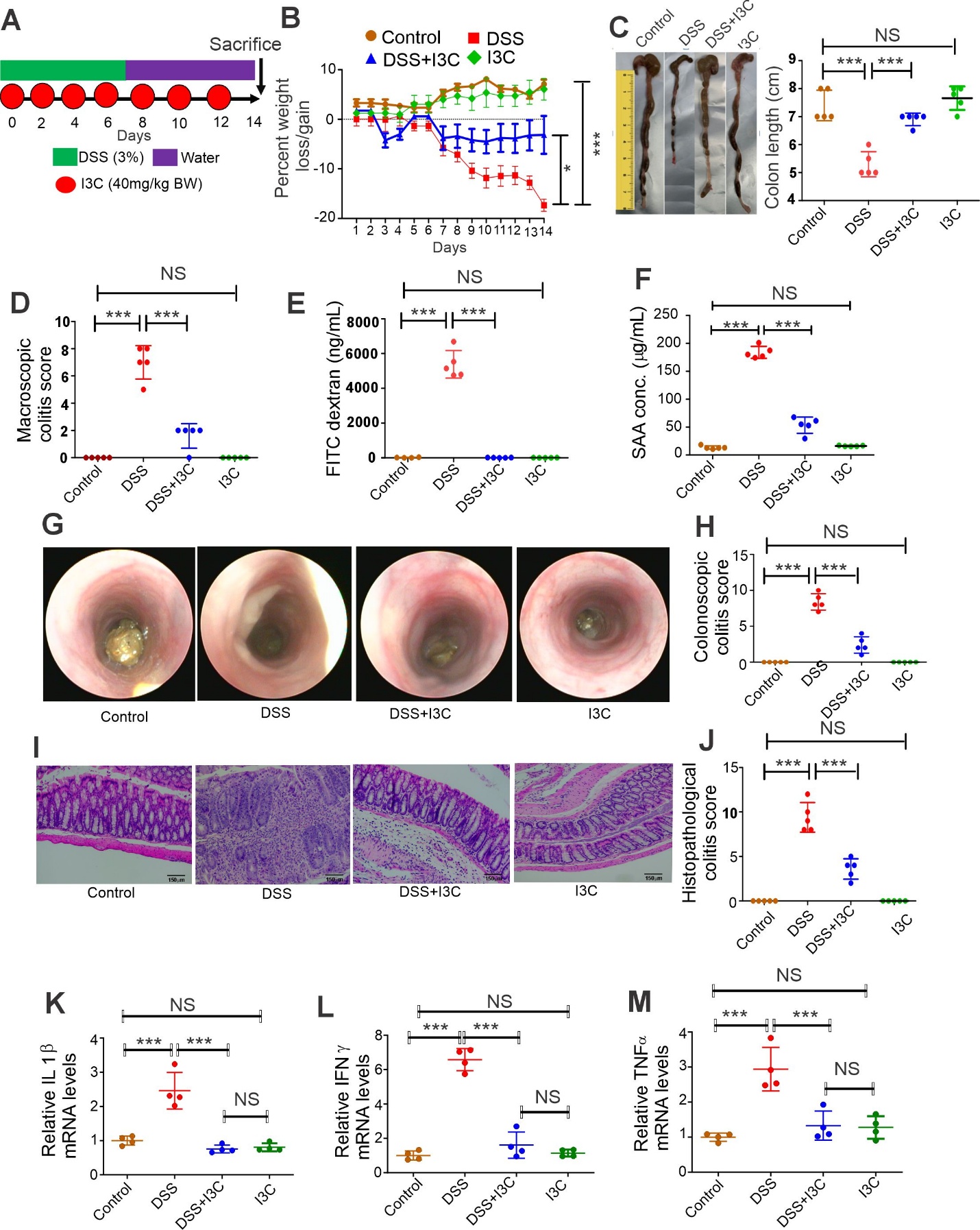
**Figure S7.** I3C treatment leads to inhibition of DSS-induced colitis. (A) Experimental design for DSS-induced colitis in mice. (B–F) Colitis was assessed by percent weight loss (B), colon length (C), macroscopic score (D), serum FITC-dextran (E) and SAA levels (F). (G) Representative colonoscopy images of the experimental and control animals. (H) Bar graph depicting colonoscopy scores from experimental mice. (I) Representative H&E stains of colons from experimental mice (*n* = 5). Scale bars: 150 μm (original magnification, ×10). (J) Bar graph depicting histopathological scores of H&E-stained colons from experimental mice (*n* = 5). (K-M) Colonic tissue from control and experimental animals was used to analyze the mRNA expression of IL1β (K), IFNγ (L), and TNFα (M) by real-time-PCR (n=4). Data are displayed as mean ± SEM; n=5. . Significance was determined using 1-way ANOVA and Tukey’s multiple comparisons test; ****p* < 0.001.


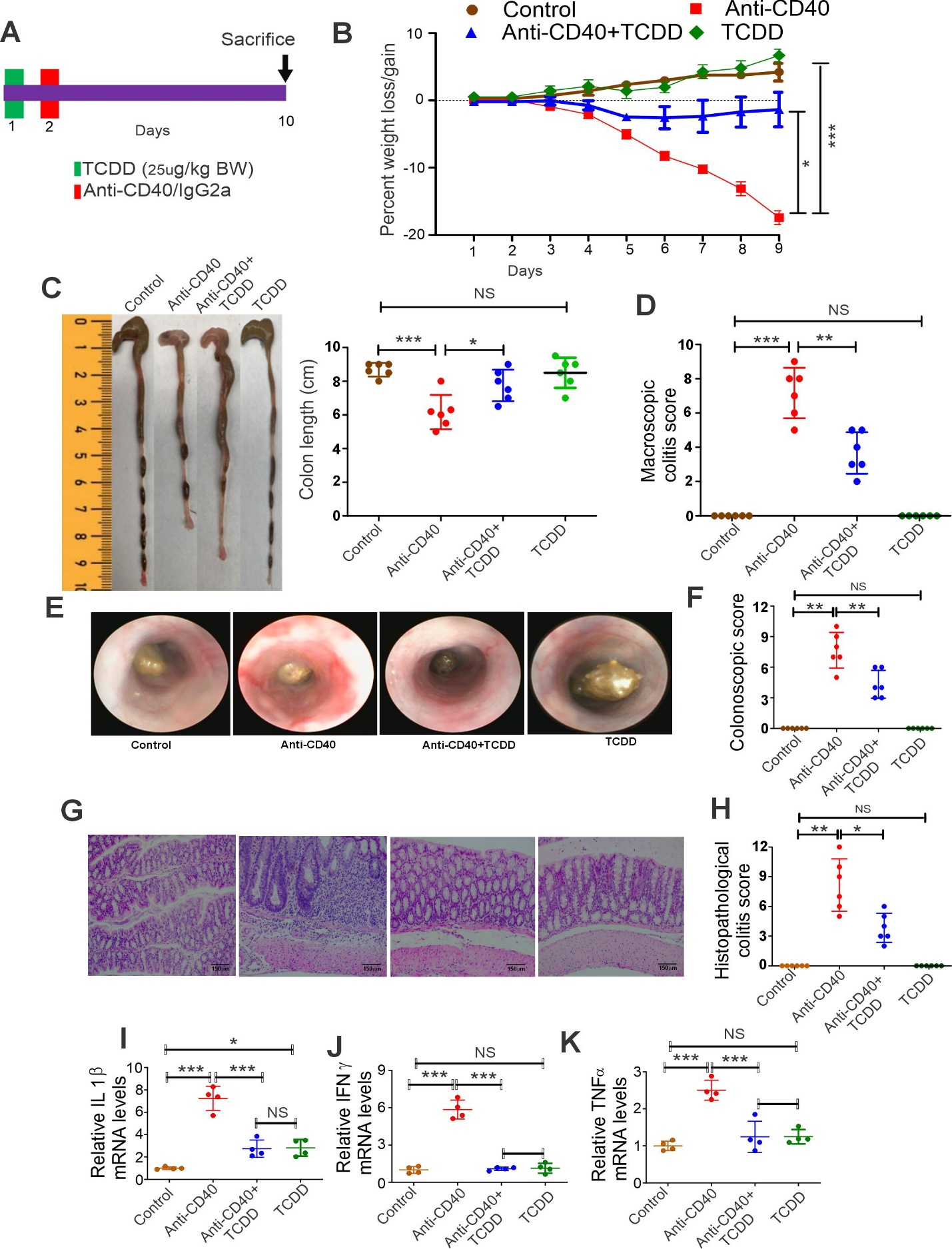
**Figure S8**. TCDD treatment attenuates anti-CD40 Ab-induced colitis. (A) Experimental design for anti-CD40-induced colitis in mice as described in Methods. (B–F) Colitis was assessed by percent weight loss (B), colon length (C), macroscopic score (D). (E) Representative colonoscopy images of the experimental and control animals. (F) Bar graph depicting colonoscopy scores from experimental mice. (G) Representative H&E stains of colons from experimental mice (n = 5). Scale bars: 150 μm (original magnification, ×10). (H) Bar graph depicting histopathological scores of H&E-stained colons from experimental mice (n = 5). (I-K) Colonic tissue from control and experimental animals were used to analyze the mRNA expression of IL1β (I), IFNγ (J), and TNFα (K) by real-time-PCR (n=4). Data are displayed as mean ± SEM; n=5. Significance was determined using 1-way ANOVA and Tukey’s multiple comparisons test; *p < 0.05; **p < 0.01; ***p < 0.001. NS=Not significant.


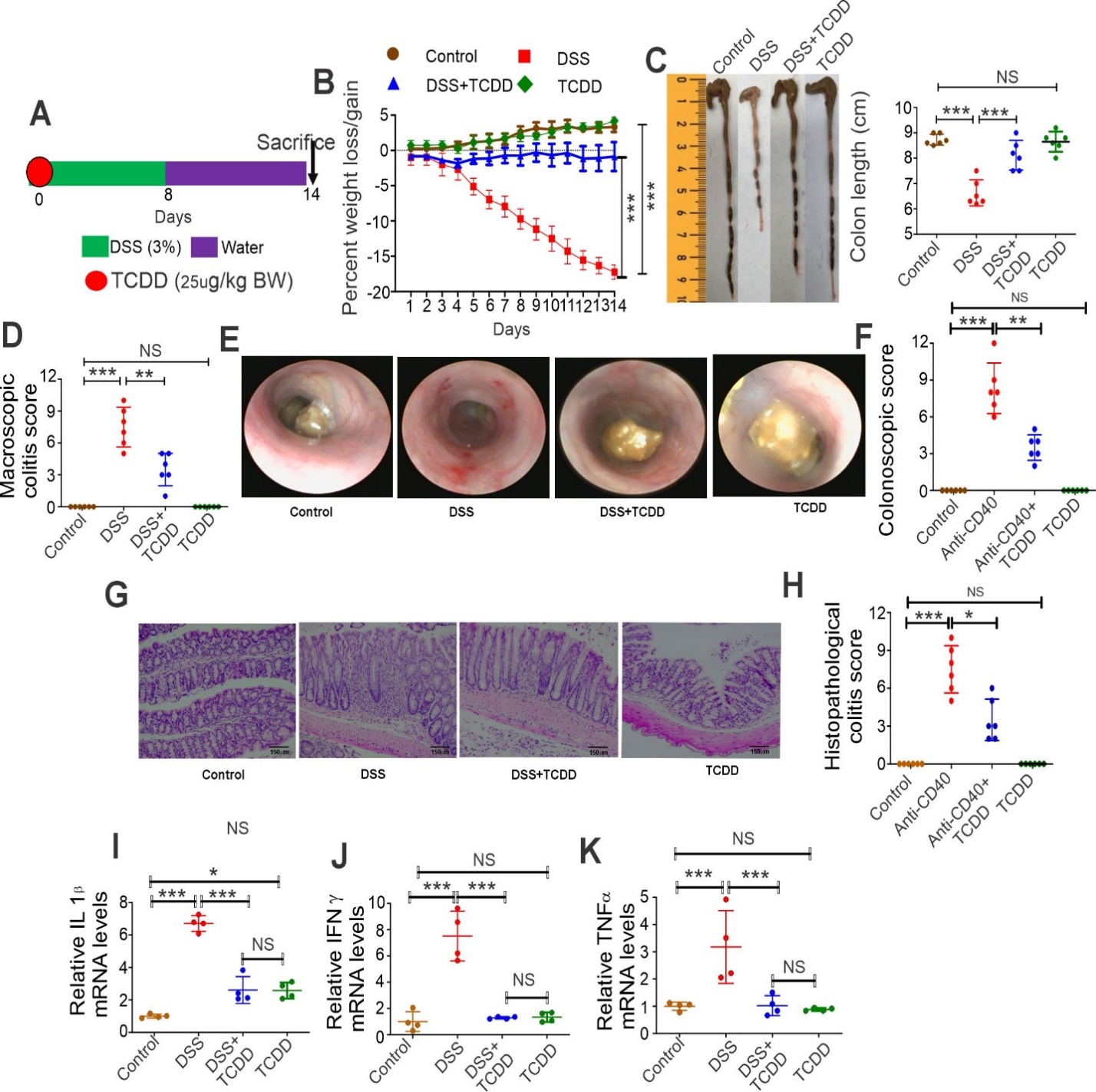
**Figure S9**. TCDD treatment attenuates DSS-induced colitis. (A) Experimental design for DSS-induced colitis in mice as described in Methods. (B–F) Colitis was assessed by percent weight loss (B), colon length (C), macroscopic score (D). (E) Representative colonoscopy images of the experimental and control animals. (F) Bar graph depicting colonoscopy scores from experimental mice. (G) Representative H&E stains of colons from experimental mice (n = 5). Scale bars: 150 μm (original magnification, ×10). (H) Bar graph depicting histopathological scores of H&E-stained colons from experimental mice (n = 5). (I-K) Colonic tissue from control and experimental animals were used to analyze the mRNA expression of IL1β (I), IFNγ (J), and TNFα (K) by real-time-PCR (n=4). Data are displayed as mean ± SEM; n=5. Significance was determined using 1-way ANOVA and Tukey’s multiple comparisons test; *p < 0.05; **p < 0.01; ***p < 0.001. NS=Not significant.


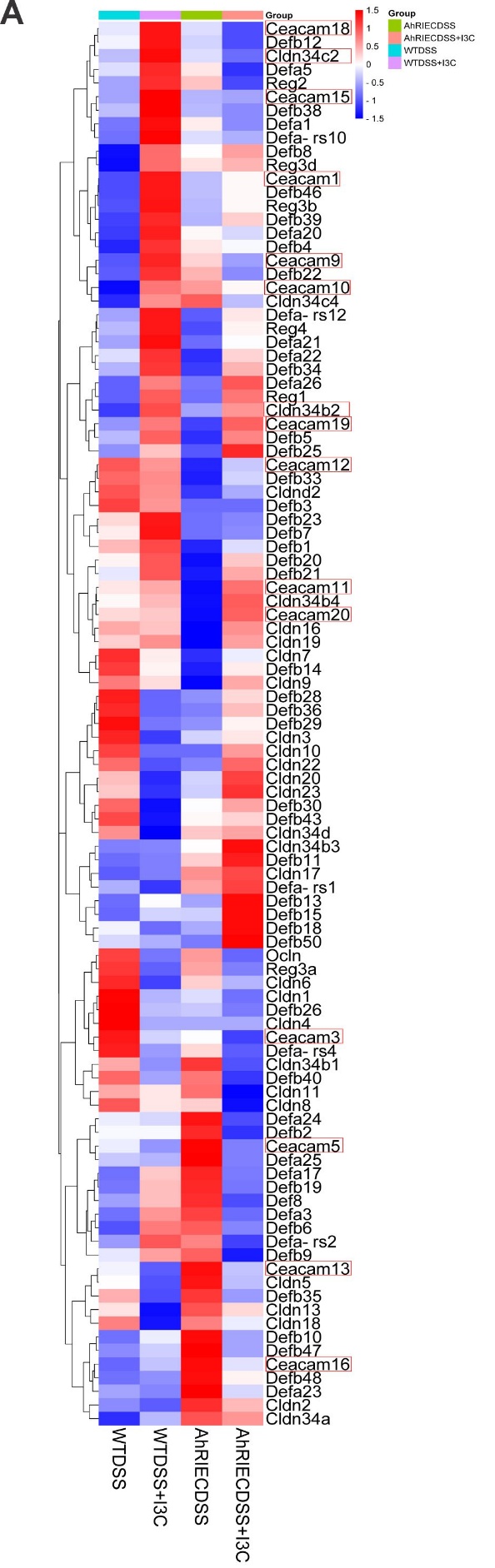


**Figure S10.** Deficiency of AhR in IECs reduces the ability of I3C to induce BD1. We enrolled (GSE242891) and performed the transcriptome microarray analysis of enriched CECs from WT and AhRΔIECs colitis mice model. (A) Heatmap using Ward's hierarchical clustering showed two-fold variations in many genes including AMPs, claudins and Ceacams among different groups.


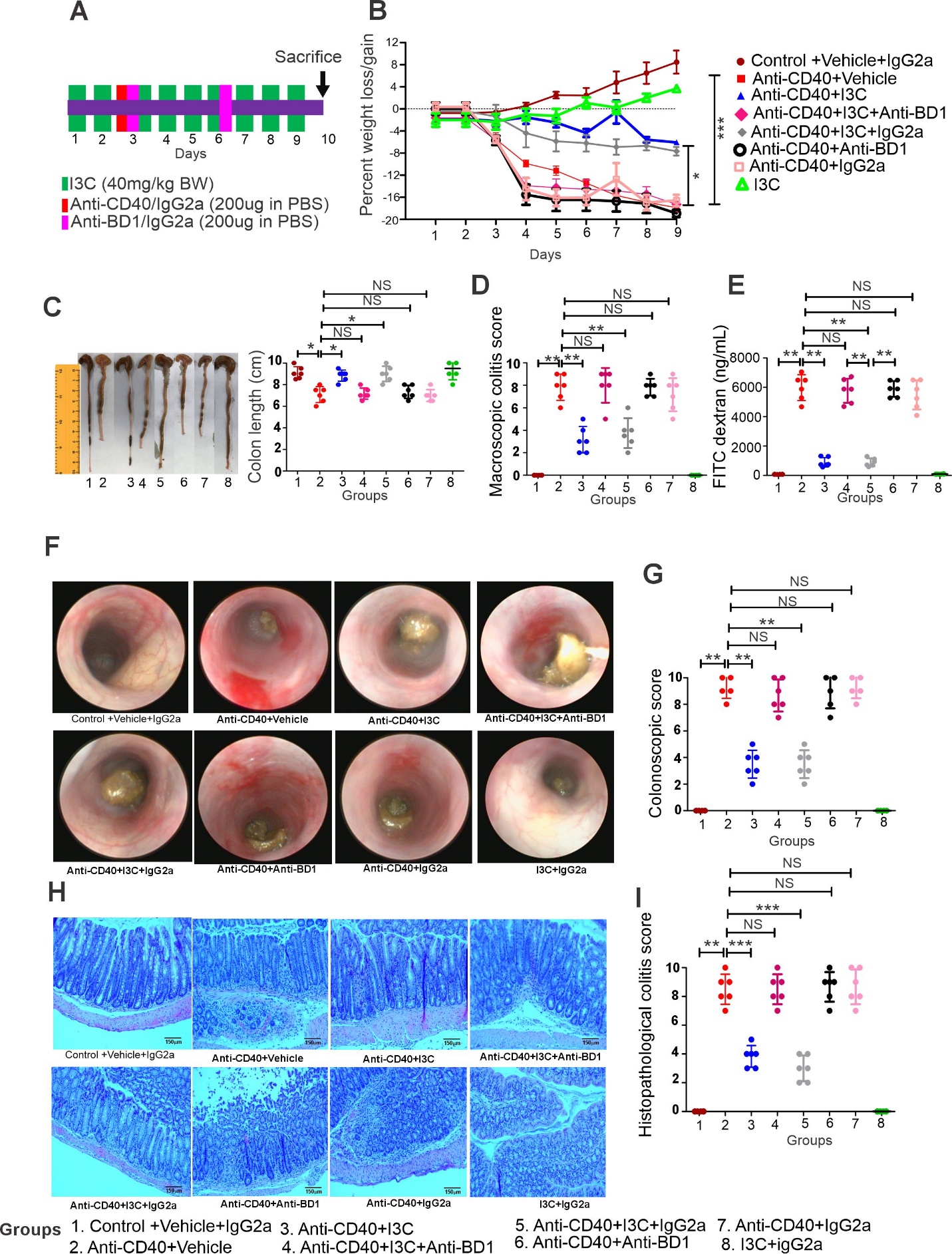
**Figure S11.** I3C failed to inhibit colitis in male mice treated with anti-BD-1 Abs. (A) Experimental design for BD-1 blocking and anti-CD40-induced colitis mice model as described in Methods. (B–F) Colitis was assessed by percent weight loss (B), colon length (C), macroscopic score (D), and FITC-dextran levels (E). (F) Representative colonoscopy images of experiments and control animals. (G) Bar graph depicting colonoscopy scores from experimental mice (n=6). (H) Representative H&E stains of colons from experimental mice (Left panel). Scale bars: 150 μm (original magnification, ×10). (I) Bar graph depicting histopathological scores of H&E-stained colons from experimental mice (Right panel) (n=6). Data are shown as mean ± SD, n=6 and significance were determined using 1-way ANOVA and Tukey’s multiple comparisons test; **p < 0.05; **p< 0.01; ***p < 0.001. NS=Not significant.


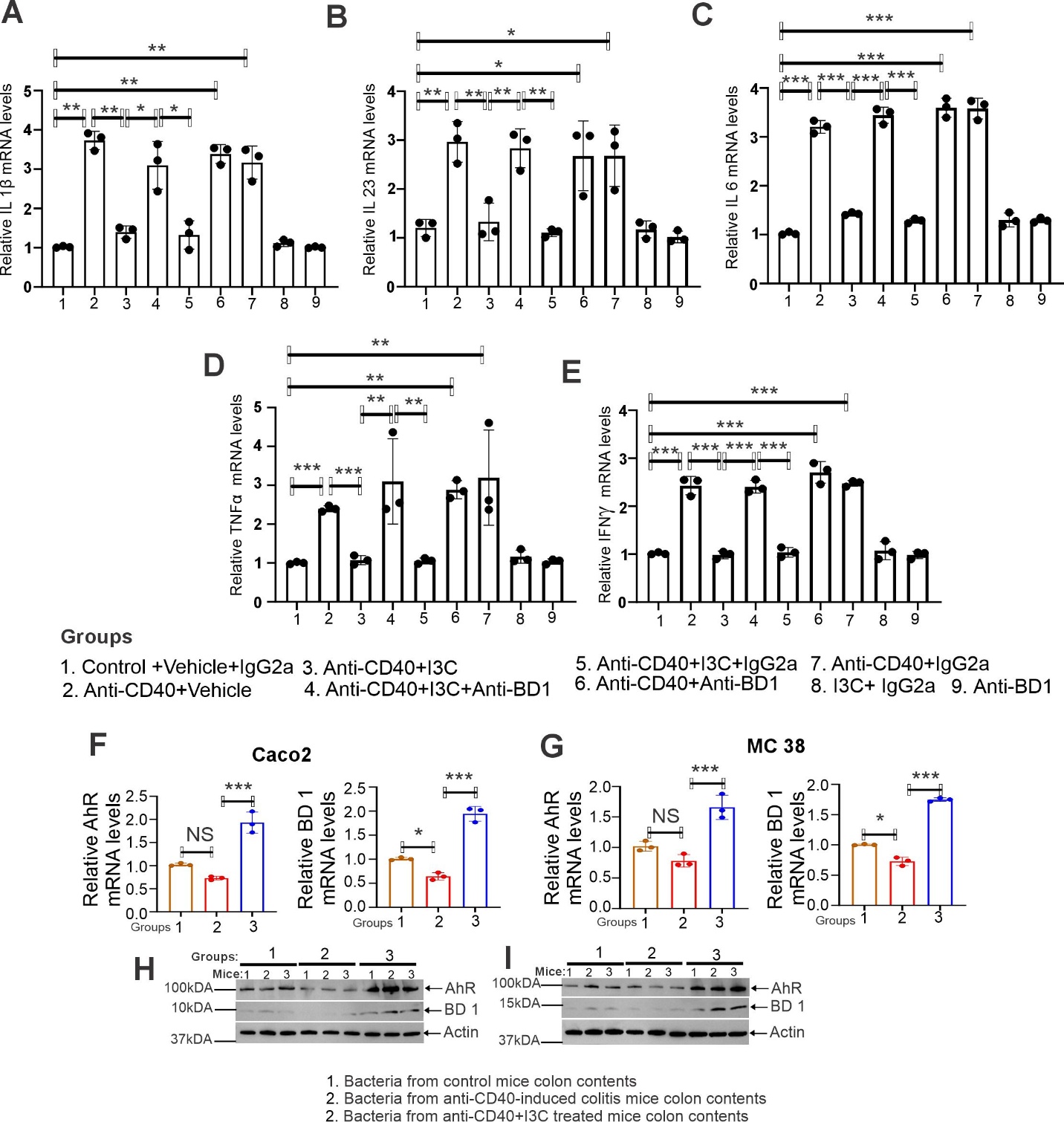


**Figure S12**. Evaluation of proinflammatory cytokines in anti-CD40-induced colitis mice with BD-1 blocking using anti-BD1 abs. (A-E) Colonic tissue from control and experimental animals were used to analyze the mRNA expression of IL1β (A), IL23 (B), IL6 (C), TNFα (D), and IFNγ (E) by real time-PCR (n=3). The bacterial population from colon contents of control, anti-CD40-induced colitis and anti-CD40+I3C treated mice were cocultured with CECs, Caco2 (F,H) and MC38 (G, I) for 24hrs and we analyzed the mRNA (F and G) and protein expression (H,I) of AhR and BD1 (n=3). Data are shown as mean ± SD. Significance was determined using 1-way ANOVA and Tukey’s multiple comparisons test; *p < 0.05; **p < 0.01; ***p < 0.005; ****p < 0.001.


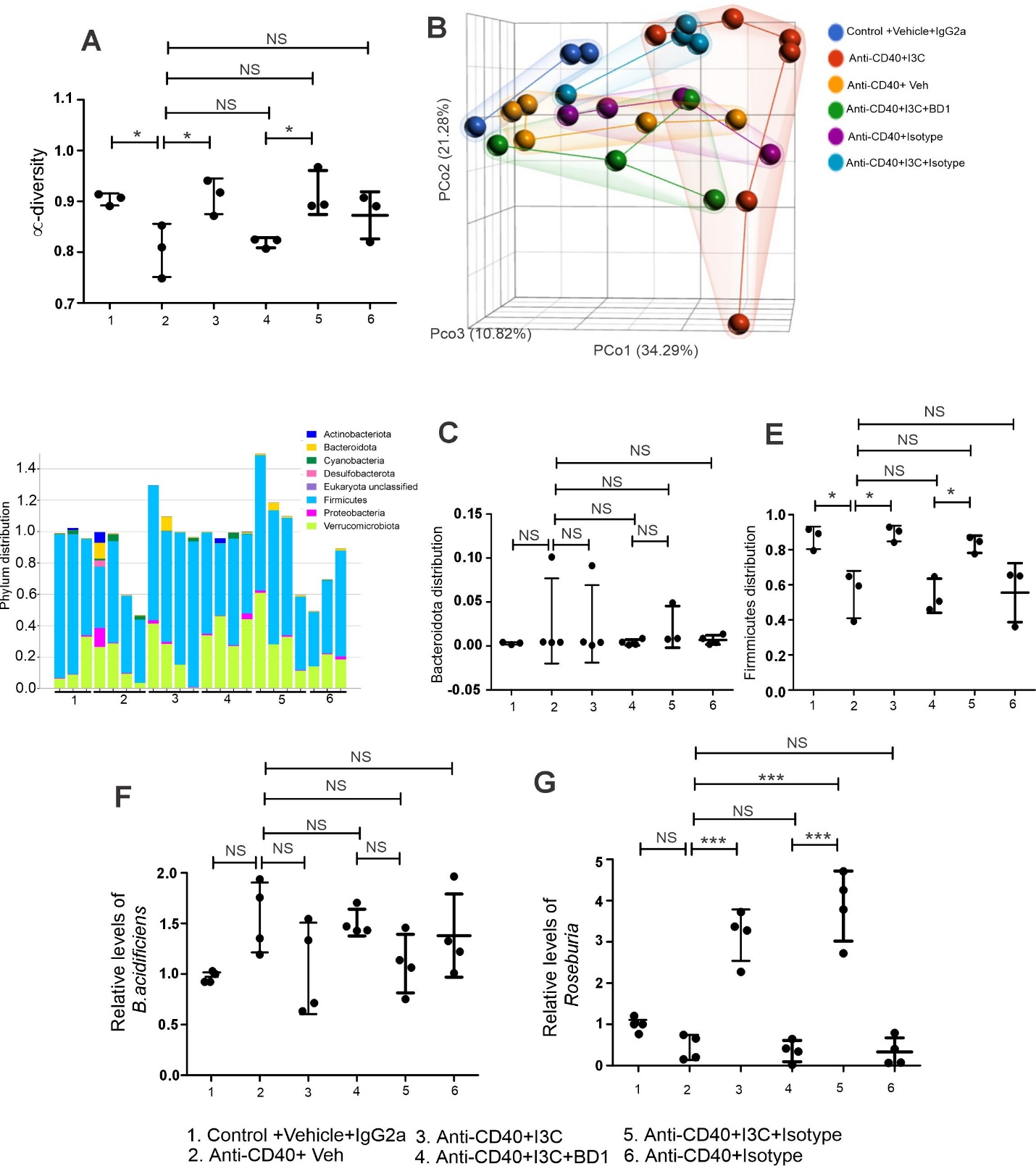
**Figure S13**. 16S rRNA sequencing from the colonic contents of control and experimental male animals was performed. Sequenced reads were analyzed using Nephele to determine chao1 α diversity (A) and β diversity by PCA plot (B). (C-E) The levels of Bacteroidota (C), Verrucomicrobiota (D) and Firmicutes (F) phylum in control and experimental animals (n=3). (F) Real time-PCR analysis was done to validate level of B. acidifaciens (F) from Bacteroidota phylum and Roseburia (G) from Firmicutes phylum (n=4). Data are shown as mean ± SD; n=5. Significance was determined using 1-way ANOVA and Tukey’s multiple comparisons test; *p < 0.05; **p < 0.01; ***p < 0.005; ****p < 0.001.

**MATERIALS AND METHODS**

**Human Tissue Samples**

Deidentified human colonic tissue specimens from patients with severe ulcerative colitis (UC), Crohn’s disease (CD), and control individuals without colitis or CD were obtained from the University of Miami. The study protocols for human subjects were approved by the Institutional Review Board of the University of Miami under IRB ID 20231111. We analyzed for the expression of AhR and BD-1 proteins by IHC using AhR (Cat#sc-133088) from Santa Cruz Biotechnology (Santa Cruz, CA) and BD1 (Cat#PA575666) from Invitrogen. IHC scores were calculated by multiplying the intensity score, which was graded as 0 (negative), 1 (weak), 2 (moderate), or 3 (strong) by the percentage of positive cells.

**Gene Expression Profiling of Colitis Patients**

Transcriptome profiling data of IBD patients, including both Crohn’s disease (CD) and Ulcerative colitis (UC) were obtained from publicly available repository Gene Expression Omnibus (GEO) with accession number GSE36807, GSE20881 and GSE11223. The raw data was downloaded into the .cel format, which was processed and filtered into the R language environment. Quality control of the microarray data was carried out using “affy” package, whereas batch effects were removed through “SVA” package obtained from Bioconductor and the differences in the expression were analyzed through “limma” package taking adjusted P-value <0.05. The Boxplot and heatmap of the expression with respect to samples were created using “ggplot”.

**Animals**

To generate IEC-specific conditional AhR knockout mice (AhR^ΔIEC^), AhR^tm3.1Bra^/J (strain#: 006203) mice were crossed with B6.Cg-Tg(Vil1-cre)1000Gum/J (strain#: 021504) mice, both of which were originally from a C57BL/6 mouse background. Mice were bred in-house at the animal facilities located at the University of South Carolina School of Medicine. Mice were genotyped by PCR analysis of DNA isolated from tail snips using DNeasy Blood & Tissue Kit (Qiagen, Hilden, Germany) and primers designed by Jackson Laboratories and purchased from IDT Technologies (Coralville, IA). To further confirm AhR cell-specific deletion in CECs, the mRNA and protein expression of AhR were measured using RT-PCR and western blot analysis in enriched CD326^+^ (or EPCAM^+^) cells from WT, and AhR^ΔIEC^ mice colons. Female C57BL/6 and B6.CB17-Prkdc^scid^/SzJ mice (8-10 weeks) purchased from the Jackson Laboratory were housed in specific pathogen-free conditions, under 12-hour light/12-hour dark cycles. All procedures were conducted in accordance with protocols approved by the Association for Assessment and Accreditation of Laboratory Animal Care (AAALAC)–accredited animal facility at the University of South Carolina School of Medicine, under protocol number 2669-101803-070723. Mice were given ad libitum access to water and a standard chow diet. Mice were given ad libitum access to water and a standard chow diet.

**Induction of colitis and treatment with I3C, neutralization of BD-1 effects and assessment of colitis parameters**

B6.CB17-Prkdc^scid^/SzJ mice were used for the anti-CD40 Ab-induced colitis model, while female C57BL/6 mice and AhR^ΔIEC^ were used for the DSS-colitis studies. We employed the previous method to induce anti-CD40- and DSS-induced colitis [1]. Briefly, mice were injected intra peritoneal (i.p.) with the anti-CD40, IgG2a monoclonal antibody FGK45 (200 µg in PBS). For treatment with I3C, mice were pretreated with 100 μL i.p. injections of I3C (40 mg/kg in 0.05% DMSO/ corn oil) or VEH 48hrs prior to administration of anti-CD40 and continued every other day until the end of the experiment [1c]. Single dose of TCDD (25 µg/kg body weight in 100uL of corn oil) was administered to mice by i.p., injection 24hrs before DSS exposure or anti-CD40 administration as described previously [2]. To inhibit BD-1, mice were dosed i.p. with anti-BD-1 (200 µg in PBS) two times between the experiment, as described in fig. 6A. To induce colitis using DSS, C57BL/6 mice were given either water or drinking water containing 3% DSS for seven days followed by a week of regular drinking water. I3C treatment (i.p. injections of 40 mg/kg in 0.05% DMSO/corn oil) was given 1 hour after introduction of DSS and continued every other day until completion of the experiment. Blood, colonic contents, and colon tissue samples were obtained at the end of the experiment. After washing with phosphate-buffered saline, the colon was sliced longitudinally, formalin fixed, and paraffin embedded.

Colitis disease parameters are assessed as described previously [1c, 3]. We measured weight daily and colon lengths at the end of the experiments. Macroscopic colitis scores were determined based on previous reports [3a]. The FITC-dextran assay was used to test *in vivo* gut permeability [3b]. Briefly, mice were administered orally with 4kD FITC-dextran (Sigma-Aldrich) (600 mg/kg) dissolved in 100 μL of PBS. After four hours, blood was collected from mice by retroorbital bleeding, and FITC-dextran concentrations were determined using a Biotek Synergy H4 multimode microplate reader with excitation wavelength at 480 nm. SAA levels from serum were measured on day 8 of the anti-CD40 model and day 10 of the DSS model using a SAA mouse ELISA (Abcam) according to the procedures provided by the manufacturer. Colonoscopy images were captured on day 9 of the anti-CD40 model and day 13 of the DSS model using a Karl Storz Tele Pack Vet X LED endoscope designed for small animals. The colonoscopy score was assessed as described previously [4]. Histological scores were assessed in formalin-fixed colonic tissue sections stained with hematoxylin and eosin based on a combination of colonic tissue damage and infiltration of inflammatory cells. Images were captured on Discover ECHO Microscopes.

**Cell lines**

CECs, MC38 (Murine adenocarcinoma) and Caco2 (Human colorectal adenocarcinoma) were purchased from ATCC. To authenticate the identity of cell lines, the ATCC employs PCR-based assays, karyotyping, and other methods. MC38 and Caco2 cells were maintained in Dulbecco’s modified Eagle’s medium and EMEM medium containing 10% FBS (Thermo Fisher Scientific) respectively. All cells were cultured in a humidified atmosphere with 5% CO_2_ at 37°C.

**Antibodies, Vectors, RNAi, and chemicals**

The following antibodies were used for western blotting and IHC and immunofluorescence staining: AhR (Cat#sc-133088) from Santa Cruz Biotechnology (Santa Cruz, CA); AhR (Cat#MA1-514), and BD1 (Cat#PA575666) from Invitrogen (Rockford, IL); β-Actin (Cat#A5441) from Sigma-Aldrich (St. Louis, MO); PE anti-mouse CD326 (Ep-CAM) Antibody Cat#118206, APC anti-mouse CD45.2 Antibody (Cat#109814), Mouse IgG2a (Cat#400202) from BioLegend (San Diego, CA); normal rabbit IgG (Cat#2729) and HRP-conjugated anti-rabbit IgG (Cat#7074) from Cell Signaling Technology (Danvers, MA); HRP-conjugated anti-mouse IgG (Cat#W4028) from Promega; Rat IgG2a isotype control (Cat# BE0089) and Rat Anti-CD40 Recombinant Antibody (clone FGK4.5) (Cat#BE0016-2) from BioXcell (Lebanon, NH); Goat anti-Mouse IgG (H+L) Highly Cross-Adsorbed Secondary Antibody, Alexa Fluor™ 488 (Cat# A-11029), Goat anti-Mouse IgG (H+L) Cross-Adsorbed Secondary Antibody, Alexa Fluor™ 568 (Cat# A-11004), and Goat anti-Rabbit IgG (H+L) Cross-Adsorbed Secondary Antibody, Alexa Fluor™ 568 (Cat# A-11011) from Thermo Fisher Scientific.

pGL-3 and renilla luciferase plasmids were purchased from addgene. AhR siRNAs (Cat#sc-29654 (H) and sc-29655 (M)), control siRNA (catalog sc-37007), AhR shRNA (m) Lentiviral Particles (Cat#sc-29655-V) and Control shRNA Lentiviral Particles-A (Cat# sc-108080) were from Santa Cruz Biotechnology. Indole-3-carbinol (I3C) (I7256-5G) was obtained from Sigma-Aldrich. Lipfectamine 3000 (L3000-015), RNAiMAX (Cat#13778-15), Halt™ Protease Inhibitor Cocktail (100X) (Cat#78430) and RIPA Lysis and Extraction Buffer (Cat#89901) were purchased from Thermo Fisher Scientific. Dextran Sulfate Sodium (DSS) (Cat#160110) was purchased from MP Biomedicals. TA Cloning™ Kit, with pCR™2.1 Vector and One Shot™ TOP10F' Chemically Competent E. coli (Cat# K203001) was purchased from Thermo Fisher.

**Generation of the mBD-1 Promoter Reporter Constructs**

The mBD-1 promoter-reporter construct (pGL-3-BD1Pro) made in pGL3- basic vector, a luciferase reporter plasmid (Promega), was kindly provided by Dr. T. Ratliff (University of Iowa, Iowa City, Iowa). In this construct, mBD-1 promoter region (105 bp) located ~9kb upstream of transcription start site (TSS) of mBD-1 was amplified using the upstream primer 5’- TCCATATTTACGGACCGAC-3’ and the downstream primer 5’- GAACAGGCATTTCCAGTCTT-3’. PCR products were cloned directly into the TOPO-TA vector pCR™2.1 Vector according to manufacturer's instructions. KPNI and XhoI digested products containing the BD1 promoter regions from TOPO-TA vector were cloned into pGL3- basic vector, which was double-digested with KPNI and XhoI. This construct was named as pGL3BD1 or pGL3-DRE1+DRE2. pGL3-ΔDRE1, pGL3-ΔDRE2, and pGL3-ΔDRE1+ΔDRE2 were generated using the below primers:

BD1-ΔDRE1-F-5’- TATTCTACCGGAGACACGCCCACCCTCGGCT-3’

BD1-ΔDRE1-R-5’- AGCCGAGGGTGGGCGTGTCTCCGGTAGAATA-3’

BD1-ΔDRE2-F-5’- TATTCTACCGGAGCGTGGACCACCCTCGGC-3’

BD1-ΔDRE2-R-5’- GCCGAGGGTGGTCCACGCTCCGGTAGAATA-3’

BD1-ΔDRE1+ΔDRE2-F-5’-AAATATTCTACCGGACCACCCTCGGCTCCTTCAAAGA-3’

BD1-ΔDRE1+ΔDRE2-R-5’-TCTTTGAAGGAGCCGAGGGTGGTCCGGTAGAATATTT-3’

Each pair of oligos were annealed and cloned directly into pCR™2.1 Vector. KPNI and XhoI digested products containing the BD1 mutant promoter regions from pCR™2.1 Vector were cloned into pGL3- basic vector, which was double-digested with KPNI and XhoI. These constructs were named as pGL3-ΔDRE1, pGL3-ΔDRE2, and pGL3-ΔDRE1+ΔDRE2, respectively. These constructs were confirmed both by restriction digestion and DNA sequencing.

**Transfections and generation of stable cell lines**

Transient transfections of plasmids and siRNAs were performed using Lipfectamine 3000 and RNAiMAX respectively according to the procedures provided by the manufacturer. shRNA stable cell lines were generated by infection of MC38 cells with AhR shRNA Lentiviral Particles and control shRNA Lentiviral Particles-A, followed by selection with puromycin (10 μg/mL).

**I3C treatment of cells**. Briefly, CECs were pretreated with I3C at 0.1, 1 and 10µM for 2hrs and then treated with DSS (0.03%) for an additional 16 hours. Cells were then collected after 16hrs and analyzed for mRNA and protein expression of AhR and BD-1 by RT-PCR and Western blotting respectively.

**Reporter assays**

MC38 cells were transiently transfected with pGL-3 reporter plasmids containing AhR binding DRE region derived from the mBD1 promoter region together with the renilla reporter vector. Renilla and firefly luciferase activities were measured using the Dual-Luciferase kit (Promega, USA) according to the manufacturer’s instructions. All luciferase assays were normalized for transfection efficiency with a renilla reporter vector. The results shown are representative of three independent experiments performed each time in triplicate.

**Immunofluorescence**

MC38 cells growing on chamber slides were treated with I3C/DSS and then fixed with methanol and acetone mixture at a 1:1 ratio (v/v) for 5 mins. These cells were permeabilized with 0.1% Triton X-100 and then blocked with 10% normal goat serum (Life Technologies, Carlsbad, CA). These fixed cells were incubated with the primary AhR, and BD-1 (1:200) antibodies in a humidified chamber overnight at 4°C and the secondary antibody conjugated with FITC and Alexa Fluor™ 568 (Invitrogen, Carlsbad, CA) respectively for 1 hour at room temperature. After washing with PBS, the cells were mounted using DAPI-containing media (ThermoFisher Scientific, Waltham, MA) and examined under a fluorescence microscope. The quantitation of fluorescence intensity was performed with ImageJ.

**Western Blotting**

Western blotting was performed as described previously [1a]. Briefly, RIPA buffer with protease inhibitors was used to lyse the cells or tissues. The protein concentration was measured using the Bio-Rad protein assay (Bio-Rad Laboratories, Hercules, CA). An equal number of proteins were run on sodium dodecyl sulfate–polyacrylamide gel electrophoresis (SDS-PAGE) and transferred to nitrocellulose membrane (Bio-Rad Laboratories, Hercules, CA). The membrane was incubated with 5% fetal BSA for 2hrs to block non-specific binding, followed by incubation with a specific primary antibody at 4°C overnight. After washing with TBST, the membrane was incubated with anti-rabbit or anti-mouse secondary antibodies for 2 hours at room temperature and then washed 3 times with TBST for 30 minutes. Chemiluminescent reagents (Millipore) were used to detect protein bands.

**RNA extraction and Quantitative Reverse-Transcriptase PCR**

Cellular RNA was isolated using the Qiagen RNeasy Kit (Valencia, CA) and the Applied Biosystems High-Capacity cDNA Reverse Transcription Kit (Carlsbad, CA) was used for complementary DNA synthesis according to the manufacturers’ protocols. mRNA expression of AhR, BD-1, EPCAM and GAPDH from mouse and human were assessed by qPCR using the following primers:

mAhR-F:5’-CTTCTAAGCGACACAGAGAC-3’A

mAhR-R:5’-AATAACATCTTGCGGGAAGG-3’A

mBD1-F:5’-CACAGGCTTCCTGGGATATAA-3’A

mBD1-R:5’- CGCTCTGGTTGGACAACTTA -3’A

mGAPDH-F:5’- AACTTTGGCATTGTGGAAGG -3’A

mGAPDH-R:5’- CAGGGATGATGTTCTGGGCA-3’

mEpCAM-F-5’-TTGCTCCAAACTGGCGTCTA-3’

mEpCAM-R-5’-ACGTGATCTCCGTGTCCTTGT-3’

hAhR-F:5’- CCATCCCCATACCCCACTAC -3’A

hAhR-R:5’- TTCTGGCTGGCACTGATACA -3’A

hBD1-F:5’- CCTTCTGCTGTTTACTCTCTGC -3’A

hBD1-R:5’- TGGCCTTCCCTCTGTAACAG -3’A

hGAPDH-F:5’- GTCTCCTCTGACTTCAACAGCG -3’A

hGAPDH-R:5’- ACCACCCTGTTGCTGTAGCCAA-3’

**Chromatin Immunoprecipitation**

Chromatin immunoprecipitation (ChIP) analysis was performed using Pierce Magnetic ChIP kit (ThermoFisher Scientific, Waltham, MA) according to the manufacturer’s instructions with mouse nonspecific IgG as a control. Briefly, 24 hours after I3C treatment, MC38 cells were fixed with 1% formaldehyde (Sigma-Aldrich) for 10 minutes at room temperature, and the fixation reaction was stopped by adding Glycine Stop-Fix Solution. The cells were sonicated on ice to produce approximately 100 to 250-base pair DNA fragments. The chromatins were then immunoprecipitated with AhR antibody. The antibody-bound complexes were isolated using beads, followed by eluting the protein/ DNA complex and purifying the DNA. Quantitative reverse transcriptase PCR was used to analyze the binding of AhR to the promoter of BD1. Primers for ChIP quantitative polymerase chain reaction (qPCR) was as follows:

mBD1-ΔDRE-F-5’-TCCATATTTACGGACCGAC-3’

mBD1--ΔDRE-R-5’-TCCGGTAGAATATTTACAA-3

mBD1-DRE-F-5’-TCCATATTTACGGACCGAC-3’

mBD1-DRE-R-5’- TCTTTGAAGGAGCCGAGGGT-3

**Microbial 16S rRNA gene analysis**

To perform bacterial phylogenetic analysis, we performed microbial 16S rRNA sequencing analysis using genomic DNA isolated from colonic flushes from control and colitis mice as previously described [1c, 5]. The sequenced data collected on the Illumina Miseq were analyzed using the Nephele platform from the National Institute of Allergy and Infectious Diseases (NIAID) Office of Cyber Infrastructure and Computational Biology (OCICB) in Bethesda, Maryland, USA [6]. Output files were analyzed using the LefSe Galaxy web application tool developed by the Huttenhower group to determine gut microbial composition [7].

**Isolation of CECs**

CECs were isolated from control and experimental mice as described previously [8]. Briefly, colons were longitudinally excised after the intestinal contents were removed, and mucus was then gently scraped away in sterile 1X PBS. After being cut into 0.5-cm pieces, the tissue was incubated for 30 min at 37°C with shaking in sterile 1X HBSS [without Ca2+ and mg2+] containing 3% FBS, 10 mM EDTA, and 5 mM DL-dithiotreitol [DTT]. After incubation, these solutions with the colon pieces were filtered through a 100-μM filter. To allow for the sedimentation of debris, the supernatant containing the intra-epithelial cells fraction was incubated on ice for at least 10 min. The intra-epithelial cells fraction extracted from the upper portion of the supernatant was washed with PBS and used for flow cytometric, mRNA and protein analysis.

**Whole-Transcriptome Sequencing**

A total RNA was purified from CECs using the Qiagen RNA easy kit. As per manufacturer instructions, A total of 100 ng RNA from each sample (control and experimental mice) was analyzed in Illumina HiSeq 2000 using NextSeq 500/550 High Output Kit v2.5 (75 Cycles) as described previously [9]. The Illumina Tru-seq RNA sample prep kit was used for library preparation. Raw sequencing reads (50 bp single-end) were mapped to mouse genome and the accepted hits were used for assembling transcripts and estimating their abundance using PARTEK. The differentially expressed genes were determined by PARTEK workflow. The heat maps were generated using “ggplot” in R environment. The raw data were normalized to GAPDH using average expression intensity. Our data are available in NCBI’s GEO database.

**REFERENCES AND NOTES**

[1] a) U. P. Singh, N. P. Singh, B. Singh, L. J. Hofseth, R. L. Price, M. Nagarkatti, P. S. Nagarkatti, *J Pharmacol Exp Ther* **2010**, *332* (3), 829, <https://doi.org/10.1124/jpet.109.160838>; b) H. H. Uhlig, B. S. McKenzie, S. Hue, C. Thompson, B. Joyce-Shaikh, R. Stepankova, N. Robinson, S. Buonocore, H. Tlaskalova-Hogenova, D. J. Cua, F. Powrie, *Immunity* **2006**, *25* (2), 309, <https://doi.org/10.1016/j.immuni.2006.05.017>; c) P. B. Busbee, L. Menzel, H. R. Alrafas, N. Dopkins, W. Becker, K. Miranda, C. Tang, S. Chatterjee, U. Singh, M. Nagarkatti, P. S. Nagarkatti, *JCI Insight* **2020**, *5* (1), <https://doi.org/10.1172/jci.insight.127551>.

[2] N. P. Singh, U. P. Singh, B. Singh, R. L. Price, M. Nagarkatti, P. S. Nagarkatti, *PLoS One* **2011**, *6* (8), e23522, <https://doi.org/10.1371/journal.pone.0023522>.

[3] a) M. A. Engel, C. A. Kellermann, G. Burnat, E. G. Hahn, T. Rau, P. C. Konturek, *J Physiol Pharmacol* **2010**, *61* (1), 89; b) G. An, B. Wei, B. Xia, J. M. McDaniel, T. Ju, R. D. Cummings, J. Braun, L. Xia, *J Exp Med* **2007**, *204* (6), 1417, <https://doi.org/10.1084/jem.20061929>.

[4] T. Kodani, A. Rodriguez-Palacios, D. Corridoni, L. Lopetuso, L. Di Martino, B. Marks, J. Pizarro, T. Pizarro, A. Chak, F. Cominelli, *J Vis Exp* **2013**, (80), e50843, <https://doi.org/10.3791/50843>.

[5] K. N. Chitrala, H. Guan, N. P. Singh, B. Busbee, A. Gandy, P. Mehrpouya-Bahrami, M. S. Ganewatta, C. Tang, S. Chatterjee, P. Nagarkatti, M. Nagarkatti, *Eur J Immunol* **2017**, *47* (7), 1188, <https://doi.org/10.1002/eji.201646792>.

[6] N. Weber, D. Liou, J. Dommer, P. MacMenamin, M. Quinones, I. Misner, A. J. Oler, J. Wan, L. Kim, M. Coakley McCarthy, S. Ezeji, K. Noble, D. E. Hurt, *Bioinformatics* **2018**, *34* (8), 1411, <https://doi.org/10.1093/bioinformatics/btx617>.

[7] N. Segata, J. Izard, L. Waldron, D. Gevers, L. Miropolsky, W. S. Garrett, C. Huttenhower, *Genome Biol* **2011**, *12* (6), R60, <https://doi.org/10.1186/gb-2011-12-6-r60>.

[8] W. Becker, H. R. Alrafas, P. B. Busbee, M. D. Walla, K. Wilson, K. Miranda, G. Cai, V. Putluri, N. Putluri, M. Nagarkatti, P. S. Nagarkatti, *J Crohns Colitis* **2021**, *15* (6), 1032, <https://doi.org/10.1093/ecco-jcc/jjaa253>.

[9] M. Bam, X. Yang, E. E. Zumbrun, J. P. Ginsberg, Q. Leyden, J. Zhang, P. S. Nagarkatti, M. Nagarkatti, *Transl Psychiatry* **2017**, *7* (8), e1222, <https://doi.org/10.1038/tp.2017.185>.


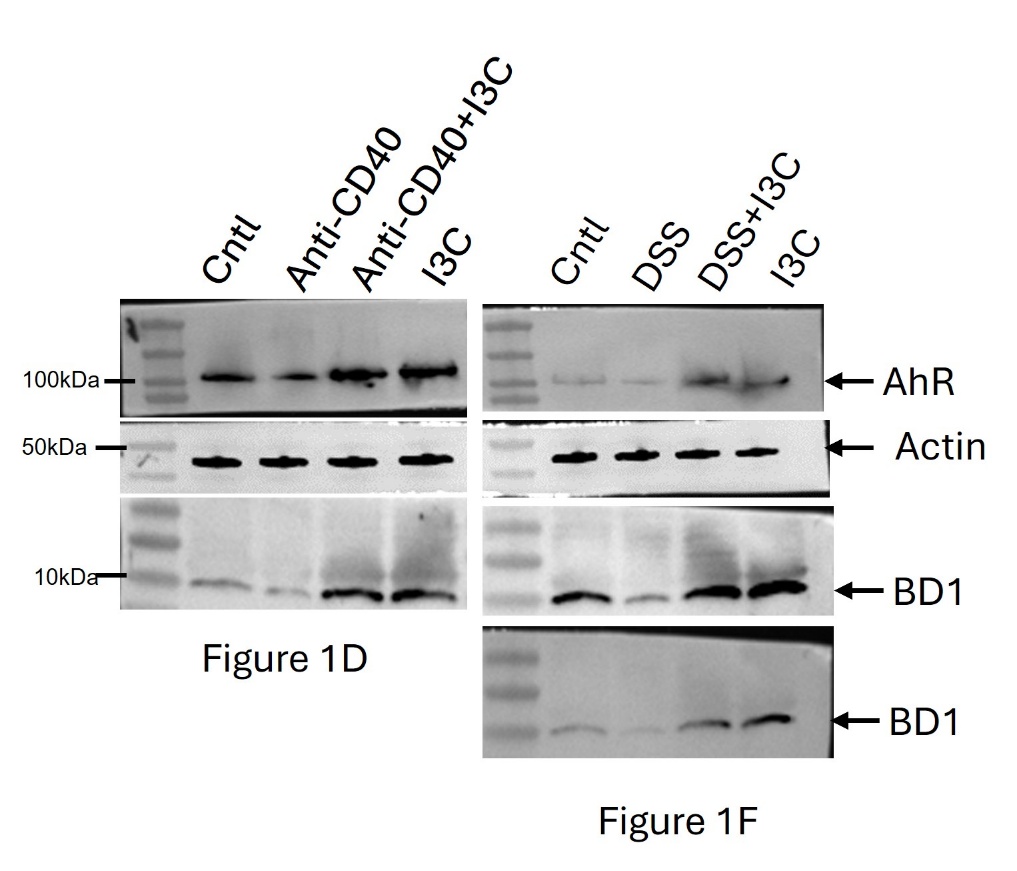


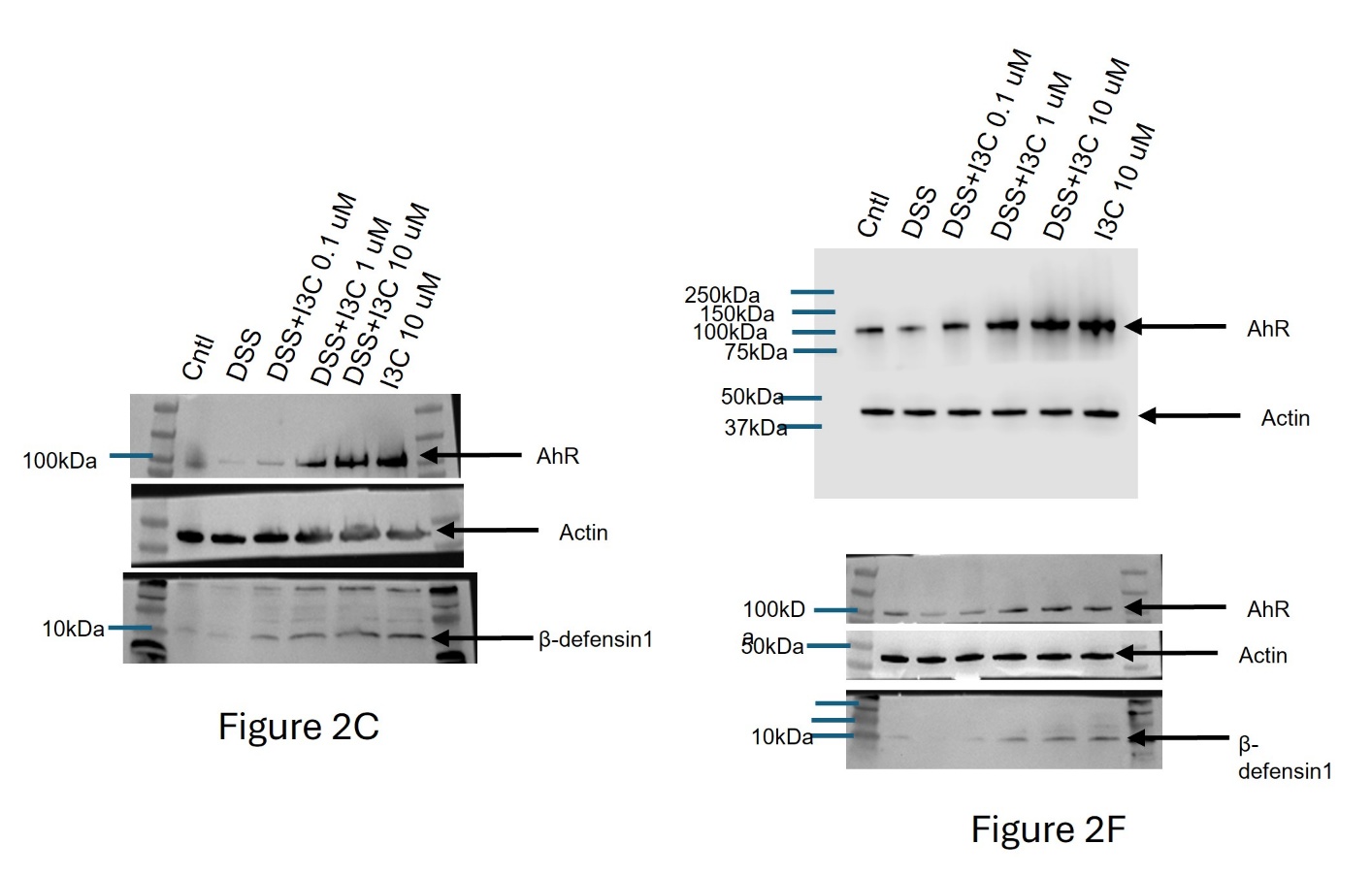


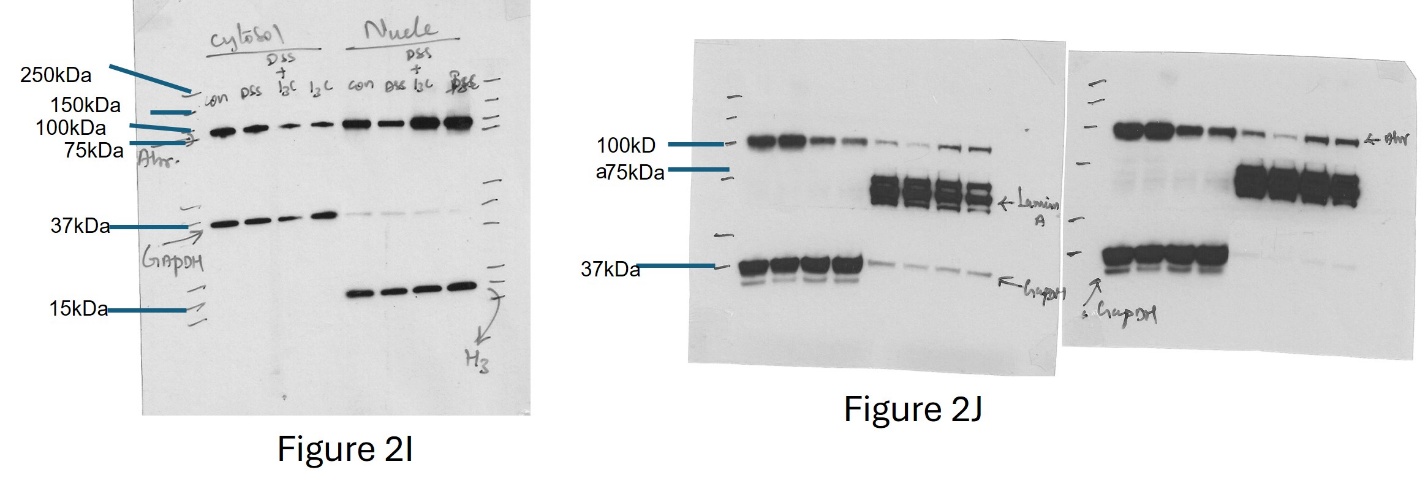


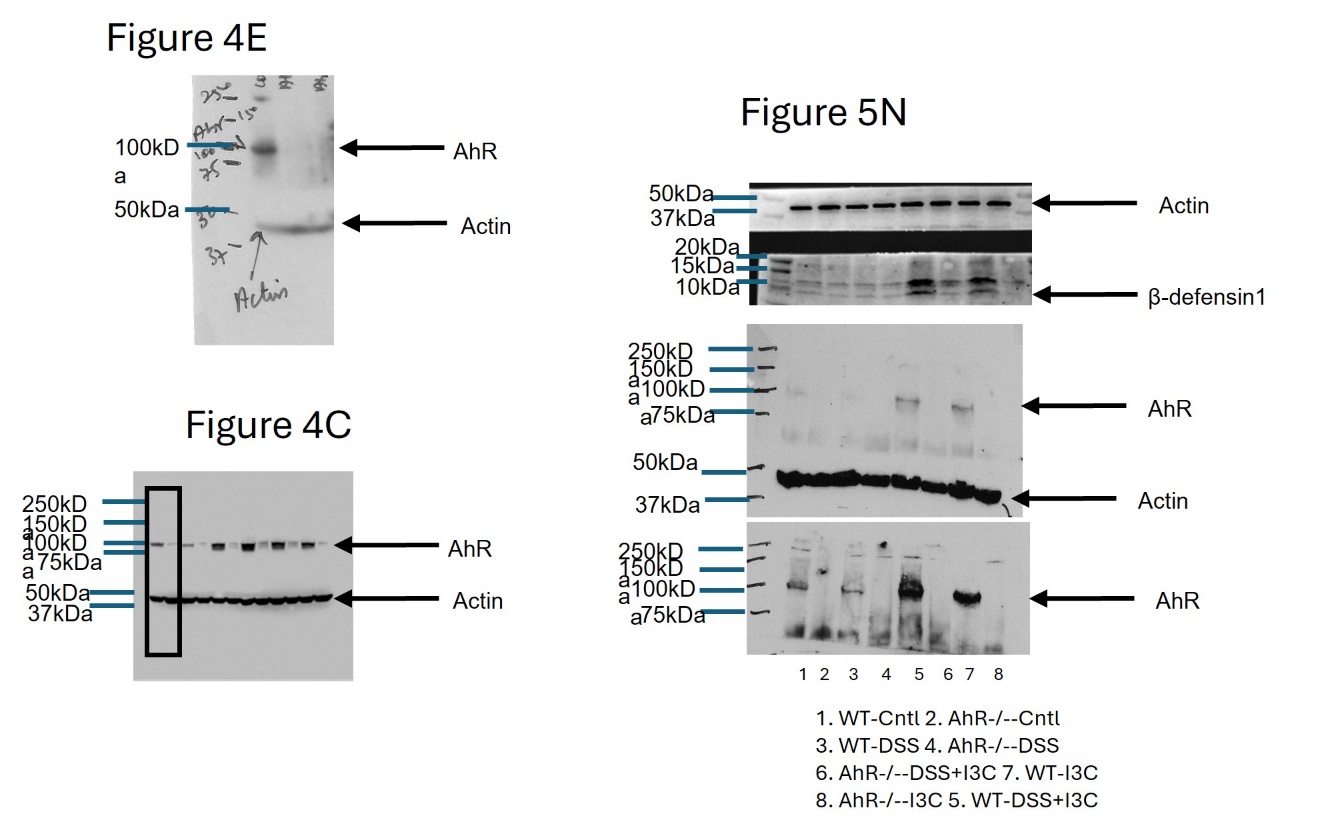


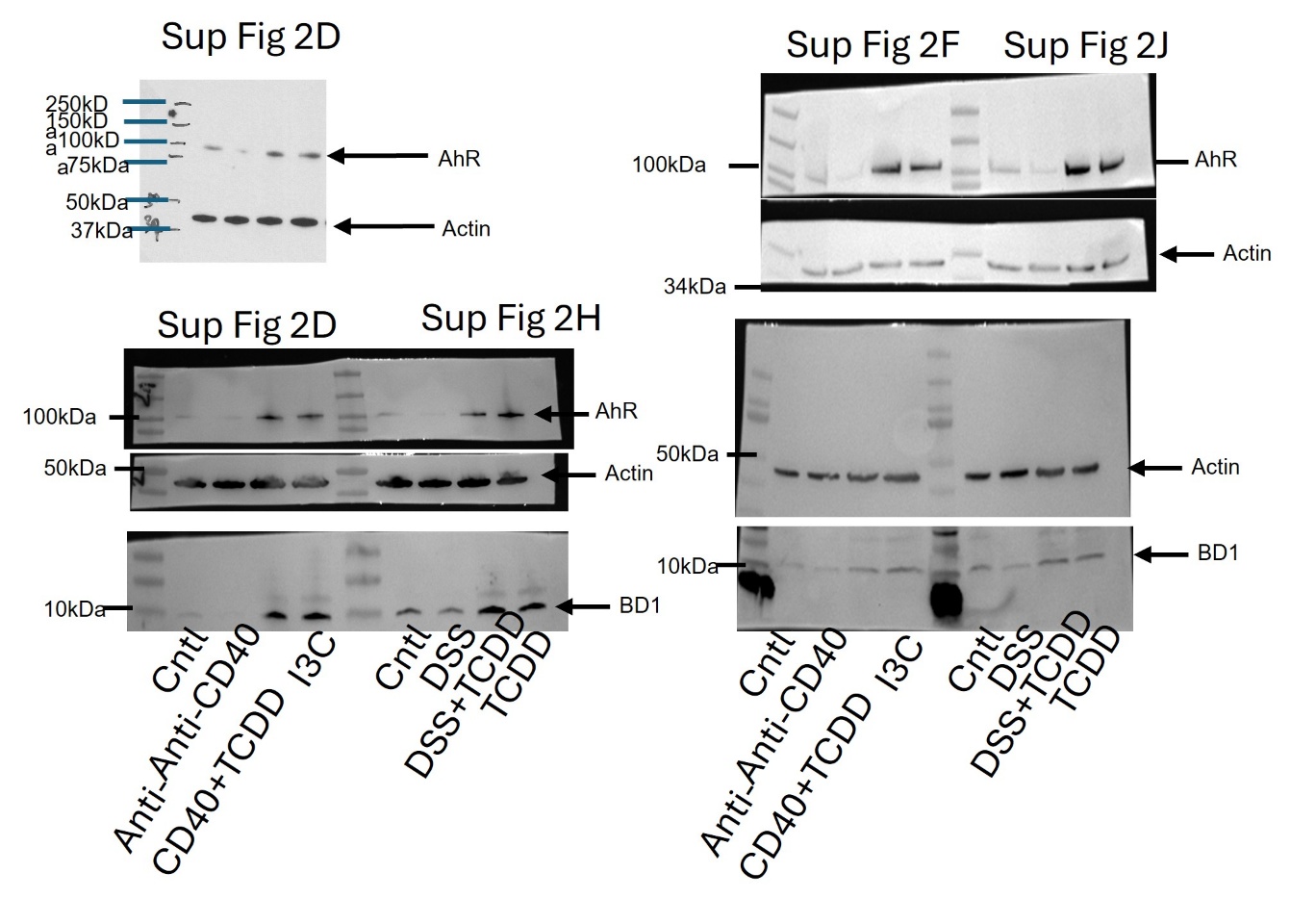


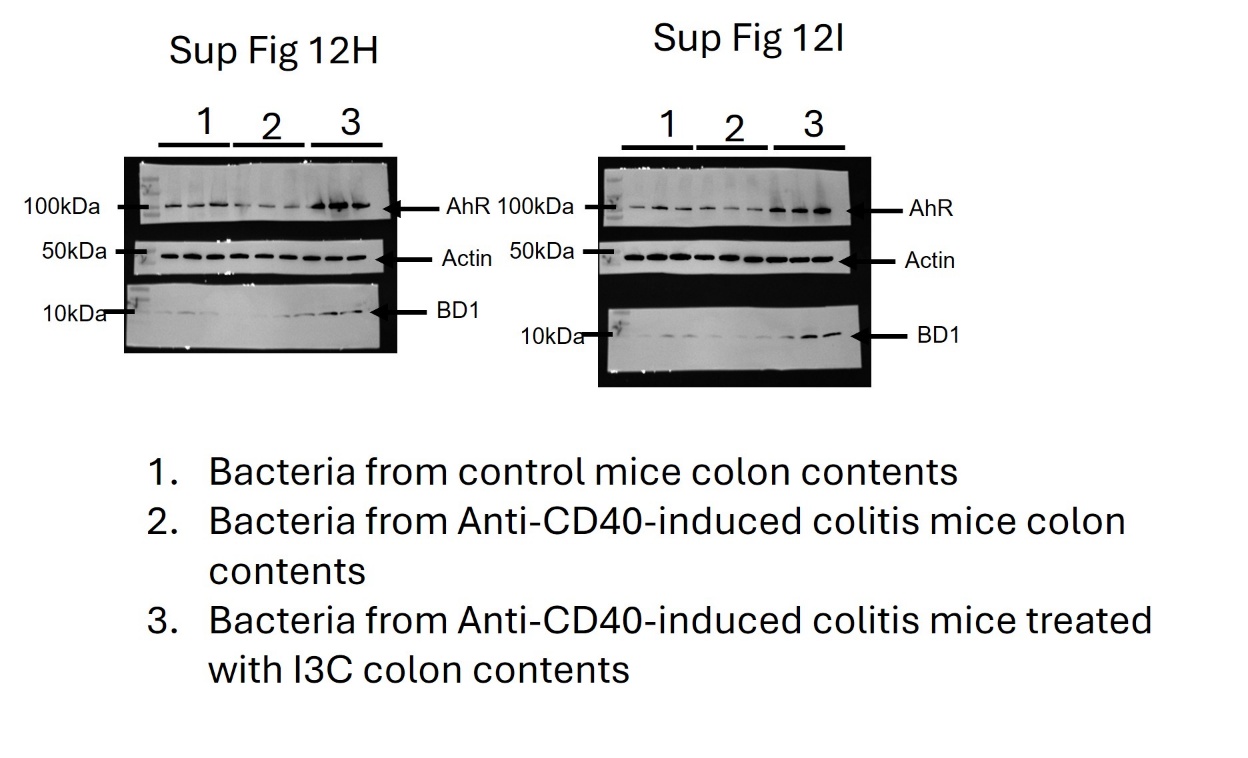

Supplement: Supplementary file 1 — Supporting Information [file ADVS-12-2416324-s001.docx]
